# Molecular Epidemiology of Penicillin-Susceptible *Staphylococcus aureus* Bacteremia in Australia and Reliability of Diagnostic Phenotypic Susceptibility Methods to Detect Penicillin Susceptibility

**DOI:** 10.3390/microorganisms10081650

**Published:** 2022-08-15

**Authors:** Geoffrey W. Coombs, Nicholas W. T. Yee, Denise Daley, Catherine M. Bennett, James O. Robinson, Marc Stegger, Princy Shoby, Shakeel Mowlaboccus

**Affiliations:** 1 Antimicrobial Resistance and Infectious Diseases (AMRID) Research Laboratory, Murdoch University, Murdoch, WA 6150, Australia; 2 Department of Microbiology, PathWest Laboratory Medicine-WA, Fiona Stanley Hospital, Murdoch, WA 6150, Australia; 3 Institute of Health Transformation, Deakin University, Melbourne, VIC 3125, Australia; 4 Department of Infectious Diseases, Fiona Stanley Hospital, Murdoch, WA 6150, Australia; 5 Department of Infectious Diseases, Royal Perth Hospital, Perth, WA 6003, Australia; 6 Department of Bacteria, Parasites and Fungi, Statens Serum Institute, 2300 Copenhagen, Denmark; 7 School of Biomedical Sciences, University of Western Australia, Crawley, WA 6009, Australia

**Keywords:** *Staphylococcus aureus*, penicillin-susceptible, bacteremia, whole genome sequencing, bioinformatics analysis, molecular epidemiology

## Abstract

Background: Defined by the emergence of antibiotic resistant strains, *Staphylococcus aureus* is a priority bacterial species with high antibiotic resistance. However, a rise in the prevalence of penicillin-susceptible *S. aureus* (PSSA) bloodstream infections has recently been observed worldwide, including in Australia, where the proportion of methicillin-susceptible *S. aureus* causing bacteremia identified phenotypically as penicillin-susceptible has increased by over 35%, from 17.5% in 2013 to 23.7% in 2020. Objectives: To determine the population structure of PSSA causing community- and hospital-onset bacteremia in Australia and to evaluate routine phenotypic antimicrobial susceptibility methods to reliably confirm penicillin resistance on *blaZ*-positive *S. aureus* initially classified as penicillin-susceptible by the Vitek^®^ 2 automated microbiology system. Results: Whole genome sequencing on 470 PSSA collected in the 2020 Australian Group on Antimicrobial Resistance Australian *Staphylococcus aureus* Sepsis Outcome Programme identified 84 multilocus sequence types (STs), of which 79 (463 isolates) were grouped into 22 clonal complexes (CCs). The dominant CCs included CC5 (31.9%), CC97 (10.2%), CC45 (10.0%), CC15 (8.7%), and CC188 (4.9%). Many of the CCs had multiple STs and *spa* types and, based on the immune evasion cluster type, isolates within a CC could be classified into different strains harboring a range of virulence and resistance genes. Phylogenetic analyses of the isolates showed most CCs were represented by one clade. The *blaZ* gene was identified in 45 (9.6%) PSSA. Although multiclonal, approximately 50% of *blaZ*-positive PSSA were from CC15 and were found to be genetically distant from the *blaZ*-negative CC15 PSSA. The broth microdilution, Etest^®^ and cefinase, performed poorly; however, when the appearance of the zone edge was considered; as per the EUCAST and CLSI criteria, disc diffusion detected 100% of *blaZ*-positive PSSA. Conclusions: In Australia, PSSA bacteremia is not caused by the expansion of a single clone. Approximately 10% of *S. aureus* classified as penicillin-susceptible by the Vitek^®^ 2 harbored *blaZ*. Consequently, we recommend that confirmation of Vitek^®^ 2 PSSA be performed using an alternative method, such as disc diffusion with careful interpretation of the zone edge.

## 1. Introduction

*Staphylococcus aureus* is an important human pathogen that causes a wide variety of infections, ranging from relatively minor (such as boils, impetigo, and wound infections) to moderate (such as cellulitis) to severe (such as bone and joint infections, pneumonia, endocarditis, and septicaemia) [1]. Prior to the antibiotic era, the outcome of severe staphylococcal infections was poor, and consequently the introduction of penicillin in the 1940s for the treatment of *S. aureus* infections is considered one of the most important medical achievements of the twentieth century.

The penicillins, also known as the β-lactam antibiotics, produce a bactericidal effect by inhibiting the membrane-bound enzymes responsible for catalysing vital stages in the biosynthesis of the bacterial cell wall [2]. Such inhibition is due to the covalent binding of the antibiotic to one or more penicillin-sensitive enzymes known as the penicillin-binding proteins. Penicillin-resistant *S. aureus* (PRSA) produces an inducible extracellular β-lactamase (penicillinase) which inactivates the antibiotic by hydrolysing the β-lactam ring. Although the β-lactamase-encoding structural gene, *blaZ*, is typically carried on plasmids, it can also be found on the chromosome.

β-lactamase-producing *S. aureus* was first described in 1944 by Kirby [3], followed by a rapid increase in PRSA prevalence, reaching almost 60% in some hospitals by the late 1940s [4]. During subsequent decades, the prevalence of penicillin resistance peaked worldwide at over 95% in *S. aureus*, causing community- and hospital-acquired infections [5]. Consequently, in some countries, routine testing for penicillin susceptibility was discontinued [6]. However, penicillin-susceptible *S. aureus* (PSSA) may be in a period of revival. Despite the use of non-penicillin β-lactams, the proportion of *S. aureus* invasive infections reported as penicillin-susceptible has increased worldwide [6,7,8,9,10]. In Australia, the Australian Group on Antimicrobial Resistance (AGAR) reported that approximately one in five methicillin-sensitive *S. aureus* bacteremia (SAB) episodes in Australia in 2020 were phenotypically penicillin-susceptible [11].

The optimal treatment for PSSA bacteremia remains unknown, and clinical practice guidelines that assume high levels of penicillin resistance have not been updated since the increase in *S. aureus* penicillin susceptibility. Most clinicians prescribe flucloxacillin for the treatment of PSSA. However, clinical outcomes may be better with benzylpenicillin, as it has a lower minimum inhibitory concentration (MIC) distribution, prolonged antibiotic concentration levels above the MIC, and higher levels of non-protein-bound drug in plasma [6]. Benzylpenicillin may also have a better adverse event profile than flucloxacillin, including less phlebitis, hepatotoxicity, and/or renal toxicity. In a large retrospective study comparing 915 patients with PSSA, the investigators found significantly higher 30-day mortality with flucloxacillin treatment compared to penicillin (OR 1.06, 95% CI 1.01 to 1.1; *p* = 0.03) [12].

Despite these advantages, the use of benzylpenicillin for the treatment of serious PSSA infections remains limited owing to skepticism of the clinical laboratory’s ability to reliably detect penicillinase-producing strains by traditional phenotypic methods [8]. In 2015 the Infectious Diseases Society of America guidelines on infective endocarditis did not recommend penicillin as a treatment for PSSA endocarditis [13]. The authors cited unreliable laboratory screening procedures for detecting true penicillin susceptibility, as certain disc diffusion and MIC detection methods coupled with a negative cefinase reaction were found to misclassify approximately 2% of *S. aureus* isolates as penicillin-susceptible [14].

A major concern when testing for penicillin susceptibility in *S. aureus* by phenotypic methods is that the penicillinase conferring resistance is not constitutively expressed. Consequently, different studies have questioned the reliability of phenotypic susceptibility testing [15,16]. For example, the chromogenic β-lactamase test, a rapid phenotypic test to detect *blaZ*-expressing *S. aureus*, has poor sensitivity compared to molecular methods such as PCR [14]. While imperfect, the penicillin disc diffusion test is recommended by CLSI (10 U penicillin disc) and EUCAST (1 U penicillin disc) [17,18]. Although the penicillin disc diffusion test seems to predict true penicillin susceptibility well [16], it includes a step of subjective determination of zone edge appearance, which potentially makes the test less reproducible [19].

In addition to having a reliable penicillin susceptibility method, it is important to understand why and how PSSA has re-emerged. Although many population structure studies have been performed on methicillin-resistant *S. aureus*, there is a paucity of information on PSSA. In the peer-reviewed literature, to the best of our knowledge, only three studies on PSSA bacteremia can be cited [7,10,20].

The aims of our study are, first, to use whole genome sequencing (WGS) to identify the population structure and clonal distribution of PSSA causing community- and hospital-onset bacteremia across Australia in 2020, and second, to evaluate routine phenotypic antimicrobial susceptibility methods in order to reliably confirm penicillin resistance on *blaZ*-positive *S. aureus* classified as penicillin-susceptible by the Vitek^®^ 2 automated microbiology system.

## 2. Materials and Methods

### 2.1. Isolate Collection

The 2020 AGAR Australian Staphylococcus aureus Sepsis Outcome Programme (ASSOP) included 30 laboratories servicing 49 institutions from all Australian states and mainland territories. From 1 January to 31 December 2020, the AGAR participating laboratories collected all *S. aureus* isolated from blood cultures. When isolated from a patient’s blood culture within 14 days of the first positive culture, *S. aureus* isolates with the same antimicrobial susceptibility profiles were excluded. A new *S. aureus* bacteremia (SAB) episode in the same patient was recorded if it was identified by a culture of blood collected more than 14 days after a previous positive culture. An SAB episode was designated ‘healthcare-onset’ when the first positive blood culture(s) in an episode were collected more than 48 h after admission.

The AGAR participating laboratories performed antimicrobial susceptibility testing using the Vitek^®^ 2 (bioMérieux, Paris, France) or BD Phoenix™ (Becton Dickinson, Franklin Lanes, NJ, USA) automated microbiology systems according to the manufacturer’s instructions. Identification of *S. aureus* was achieved by matrix-assisted laser desorption ionization (MALDI) using either the Vitek MS^®^ (bioMérieux, France) or MALDI Biotyper (Bruker Daltonics, Bremen, Germany). Minimum inhibitory concentration (MIC) data and isolates were referred to the Antimicrobial Resistance and Infectious Diseases (AMRID) Research Laboratory at Murdoch University. All isolates were stored as frozen glycerol stocks at −80 °C. Vitek^®^ 2 antimicrobial susceptibility testing was performed on isolates previously classified as penicillin-susceptible by the BD Phoenix™ automated microbiology system. *S. aureus* isolates identified as penicillin-susceptible by the Vitek^®^ 2, either by a participating laboratory or by AMRID, were included in the study.

### 2.2. Whole Genome Sequencing

Genomic DNA was extracted using the MagMAX^TM^-96 DNA Multi-Sample Kit (Life Technologies, Carlsbad, CA, USA, 4413021) according to manufacturers’ instructions. DNA quantification was performed using the Qubit^TM^ 1X dsDNA HS Assay Kit (Thermo Fisher Scientific, Scoresby, VIC, Australia), Q33232). Sequencing libraries were prepared using the Illumina Nextera^®^ XT DNA Library Preparation Kit (Illumina, San Diego, CA, USA, FC-131-1096) and sequenced on the NextSeq 500 platform (Illumina, San Diego, CA, USA) with 150 bp paired-end chemistry, as previously described [21].

### 2.3. Genomic Assembly and Phylogenetic Reconstruction

Adapters were trimmed using Trimmomatic and the cleaned data were de novo assembled using SPAdes v3.15.4 [22]. The contiguous sequences were annotated using Prokka v1.14 [23]. The Roary v3.11.2 pipeline was used to perform pan-genome analyses on the PSSA genomes [24]. A phylogenetic tree was constructed based on the single-nucleotide polymorphism alignment of the core-genome alignment (from Roary output) using the neighbour-joining algorithm with 200 bootstrap replicates in MEGA v11 [25]. The phylogenetic tree was annotated and visualized on the integrative Tree of Life (iTOL) website [26].

### 2.4. Bioinformatics Analyses

In silico multilocus sequence typing (MLST) was performed on de novo assemblies using mlst v2.19.0 [27,28] to assign a sequence type (ST) to each isolate. Undefined STs were submitted to the PubMLST database for ST assignment [28]. An MLST minimal spanning tree was constructed using Grapetree [29]. SpaTyper v0.3.3 (https://github.com/HCGB-IGTP/spaTyper) (accessed on 1 March 2022) was used to determine the *spa* types and Resfinder v4.0 was used to detect antimicrobial resistance (AMR) genes [30]. Mutations associated with AMR was identified using PointFinder v3.2 [31]. The genomes were screened for Panton-Valentine leucocidin (PVL)-encoding genes, immune evasion cluster (IEC) genes (*sea*, *sep*, *chp*, *sak*, *scn*), enterotoxin (-like) genes, toxin genes (*eta*, *etb*, *etd*, *edinA*, *edinB*, *edinC*, *tst*), capsule genes, and *agr* genes using BLAST [32]. Mutations in amino acid sequences encoded by *blaZ*, *blaR1* and *blaI* were identified by comparing them to the sequences of *S. aureus* ATCC^®^ 29213. BlaZ types (A-D) were assigned based on the amino acid residue at positions 119 and 207 of the translated sequence of *blaZ* [33].

### 2.5. β-Lactamase Detection

β-lactamase activity was detected using BD BBLTM Cefinase^TM^ nitrocefin paper discs (Becton Dickinson, Franklin Lakes, NJ, USA, 231650) according to the manufacturer’s instructions. A change in colour from yellow to red after one hour incubation at room temperature was recorded as a positive reaction [34]. *S. aureus* ATCC^®^ 29213 and ATCC^®^ 25923 were used as quality control isolates for positive and negative β-lactamase activity, respectively [17].

### 2.6. Penicillin Susceptibility Testing

Disc diffusion assays were performed according to CLSI [17] and EUCAST guidelines [18] using a penicillin 10U (P1) and a penicillin 1U (P10) antibiotic disc, respectively. Penicillin broth microdilution (BMD) was performed according to CLSI guidelines. The penicillin Etest^®^ (bioMérieux, 412262) was used as per the manufacturer’s recommendations. Interpretation of the penicillin MIC was determined using the EUCAST and CLSI breakpoints [17,18]. *S. aureus* ATCC^®^ 29213 and ATCC^®^ 25923 were used as quality control isolates in the BMD and disc diffusion assays, respectively [17].

### 2.7. Comparison of Proportions

Comparison of proportions were calculated using MedCalc for Windows (MedCalc Software, version 19.7.4, Ostend, Belgium) (sourced from https://www.medcalc.org, accessed on 1 March 2022).

## 3. Results

Of the 2734 SAB episodes reported in the AGAR 2020 ASSOP, 530 (19.4%) isolates were classified as PSSA (MIC ≤ 0.12 mg/L) by the Vitek^®^ 2. Of these, 479 (90.4%) were referred to the AMRID Research Laboratory. By WGS, nine isolates were identified as Staphylococcus argenteus and were excluded from the study. The 470 Vitek^®^ 2 penicillin-susceptible SAB episodes occurred across Australia, and 392 (83.4%) were classified as community-onset (Supplementary Table S1).

### 3.1. Population Structure of PSSA

**Figure 1:**
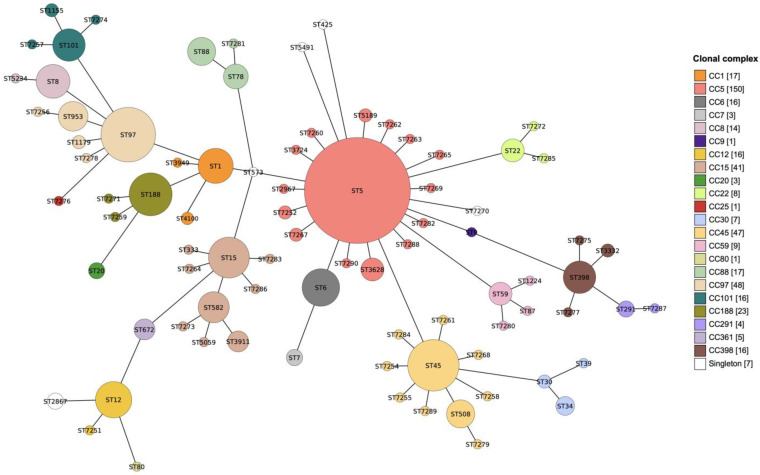
Minimal spanning tree showing distribution of MLST sequence type (ST) and clonal complex (CC). Each circle represents an ST and the size of each circle is proportional to the number of isolates described by the ST. The length of the line connecting two circles is proportional to the number of MLST loci by which the two STs differ. Each circle is colored by CC and the number of isolates in each CC is given in the color legend.

The 470 isolates consisted of 84 STs, of which 79 STs (463 isolates) were grouped into 22 CCs (Figure 1). The dominant CCs (representing > 20 isolates) included CC5 (31.9%), CC97 (10.2%), CC45 (10.0%), CC15 (8.7%), and CC188 (4.9%). Five STs (ST425, ST573, ST2867, ST5491, ST7270) consisting of seven isolates were considered singletons (Supplementary Tables S1–S3).

### 3.2. Distribution of Virulence Genes

The virulence genes identified in each CC are shown in Table 1.

The *lukS/F-PV* PVL-associated genes were detected in only three isolates: ISTOP-308 (ST88), ISTOP-311 (ST30) and ISTOP-409 (ST1).

The toxic shock syndrome toxin-1 gene, *tst*, was identified in 4.9% (*n* = 23) of isolates. Primarily associated with CC45 (*n* = 10) and CC30 (*n* = 6), *tst* was also identified in ST5 (*n* = 3), ST22 (*n* = 2) and ST97 (*n* = 2).

The epidermal cell differentiation inhibitor gene *edinB* was identified in nine isolates and was mostly associated with CC291 (*n* = 4) and ST2867 (*n* = 3). Single isolates of CC25 and CC80 also harbored *edinB*.

The exfoliative toxin gene *eta* was identified in four isolates: two CC15 isolates, and single isolates of CC1 and CC9.

Most isolates harbored staphylococcal enterotoxins (se) or staphylococcal enterotoxin-like (*sel*) genes. The *selx* gene was identified in 94.0% (*n* = 442) of isolates. The *egc*-cluster (*seg*, *sei*, *sem*, *sen*, *seo*, *seu*, *sev*, *selw*), identified in 47.0% (*n* = 221) of isolates, was restricted to eight CCs, including all CC5, CC9, CC20, CC22, CC25, CC30, and CC361 isolates and in ST573. For CC45, 45 of the 46 isolates harbored the *egc*-cluster. The *sea* gene was identified in 7.9% (*n* = 37) of isolates. Although identified in six CCs, *sea* was predominately associated with CC6 (93.8% of CC6 isolates) and CC1 (52.9% of CC1 isolates) isolates. The *sep* gene was identified in 13.8% (*n* = 65) of isolates including four CCs and in ST7270. The dominant CCs harboring *sep* included CC12 (62.5% of CC12 isolates) and CC5 (32.7% of CC5 isolates). The *seb* gene, identified in 6.2% (*n* = 29) of isolates, was predominantly associated with CC12 (62.5% of CC12 isolates) and CC59 (55.5% of CC59 isolates).

The *sec* + *sel* positive isolates (*n* = 43) included one *sec1* + *sel*, 31 *sec2* + *sel*, and 11 *sec3* + *sel* isolates, and were identified in ten CCs and in ST573. The predominant CC harboring *sec* + *sel* was CC45 (59.6% of CC45 isolates). The *sek* + *seq* genes were identified in 5.1% (*n* = 24) of isolates belonging to four CCs. The dominant CCs were CC1 (82.3% of CC1 isolates) and CC59 (55.5% of CC59 isolates). The *sed* + *selj* + *ser* genes were identified in 3.8% (*n* = 18) of isolates, most of which were CC5 (*n* = 17). The *seh* gene was identified in 4.3% (*n* = 20) of isolates predominantly in CC1 (88.2% of CC1) and CC30 (57.1% of CC30). Other enterotoxin genes identified included *selz* (*n* = 17, of which 16 belonged to CC12) *sely* (*n* = 14, of which nine belonged to CC59), *sel28* (*n* = 2) and single isolates with *sel31*, *sel32* or *see*.

### 3.3. Distribution of AMR Genes

A breakdown of the AMR genes identified in each CC is given in Table 2. The *blaZ* gene was identified in 9.6% (*n* = 45) of the 470 Vitek^®^ 2 PSSA. Although identified in 13 CCs, most *blaZ*-positive isolates were characterised as CC15 (*n* = 20, 48.8% of CC15) and CC5 (*n* = 10, 6.7% of CC5).

Most isolates did not harbor AMR genes, and 77.2% (*n* = 363) were pan-susceptible. Overall, AMR genes associated with aminoglycoside [*ant(4′)-la*, *aadD*, *ant(9)-la*, and *aph(3′)-IIIa*], trimethoprim [*dfrG*], macrolide [*ermA*, *ermB*, *ermC*, *ermT* and *mphC*], chloramphenicol [*fexA*], fusidic acid [*fusC*], lincosamide [*lnuA*], tetracycline [*tet*(K) and *tet*(M)] resistance, and the multidrug resistance *mdfA* gene were identified. In addition, mutations in *grlA* and *gyrA* (quinolone), *rpoB* (rifampicin), and *fusA* (fusidic acid) were identified. Of the 13 isolates harboring the fusidic acid resistance gene, *fusC*, 12 were CC1 isolates (70.6% of CC1). The macrolide resistance gene *ermT* was restricted to CC398 isolates (*n* = 12, 75.0% of CC398). For the nine ciprofloxacin-resistant isolates, GrlA S80F and GyrA S84L mutations were identified.

### 3.4. Clonal Complexes

#### 3.4.1. Clonal Complex 1

The 17 *agr* group III/capsule type 8 CC1 isolates included three STs (ST1 [*n* = 14 isolates], ST4100 [*n* = 2] and ST3949 [*n* = 1]). Six closely related *spa* types were identified, with t127 (*n* = 10) the most dominant. The *spa* type could not be determined for one isolate. Based on their IEC type, the 17 isolates could be classified into two closely related strains.

The nine type D IEC (*sea*, *sak*, *scn*) isolates included eight ST1 isolates, of which one was PVL-positive and one, ST3949, was a single-locus variant (slv) of ST1. All CC1 type D IEC isolates harbored the *sea*, *sek* + *seq*, *seh*, and *selx* enterotoxin genes. Single isolates also harbored *sec2* + *sel* or *seb*. Eight isolates harbored *fusC*.

The seven type E IEC (*sak*, *scn*) isolates included five ST1 isolates and two ST4100, a double locus variant (dlv) of ST1. All seven isolates harbored *selx*. Five isolates also harbored *seh* and *sek* + *seq*, and four isolates harbored *fusC*. A single isolate harbored the exfoliative toxin eta gene.

For one CC1 isolate, ST1 t127 (ISTOP152), the IEC was not detected.

#### 3.4.2. Clonal Complex 5

CC5, the predominant CC in our study, contained 150 isolates, of which 149 were *agr* group II/capsule type 5. For one capsule type 5 isolate, the *agr* could not be determined. Fifteen STs were identified, including: ST5 (*n* = 128), ST3628 (*n* = 6), ST5189 (*n* = 2), ST7252 (*n* = 2), ST7267 (*n* = 2), and single isolates of ST2967, ST3724, ST7260, ST7262, ST7263, ST7265, ST7269, ST7282, ST7288, and ST7290. Forty-four closely related *spa* types were identified, with t002 (*n* = 55) the most dominant. The *spa* type could not be determined for one isolate. Based on the IEC type, the 150 isolates could be classified into six closely related strains.

The three type A IEC (*sea*, *sak*, *chp*, *scn*) ST5 isolates, harbored the *sea*, *selx*, and *egc*-cluster enterotoxin genes. Single isolates harbored either *ermC* or *tet*(M) and *fexA*.

Seventy (46.7% of CC5) isolates harbored a type B IEC (*sak*, *chp*, *scn*), of which 57 were ST5 and six were ST3628, a ST5slv. The remaining seven isolates were also ST5slvs, and included ST7267 (*n* = 2) and single isolates of ST2967, ST5189, ST7260, ST7269, and ST7288. All isolates harbored *selx* and the *egc*-cluster. Ten isolates harbored *sed* + *selj* + *ser* enterotoxin genes. Six isolates harbored *blaZ*, three isolates *ermC* (of which two harbored *blaZ*). and one isolate *drfG*. One of the *blaZ*-positive isolates harbored multiple AMR genes, including *ant(a)-1a*, *ermA*, *ermC*, and *tet*(K).

Of the five CC5 isolates that harbored a type D IEC, three were ST5 and the remaining two were ST5slvs (ST3723 and ST7290). These five isolates harbored *selx* and the *egc*-cluster, and one harbored *sed* + *selj* + *ser*. A single isolate harbored *tet*(M).

Sixteen (10.7% of CC5) of the CC5 isolates harbored a type E IEC, of which 13 were ST5. Two isolates were ST5slvs: ST5189 and ST7282. The remaining isolate was ST7265, a ST5dlv. These 16 isolates harbored *selx* and the *egc*-cluster. A single isolate harbored *sed* + *selj* + *ser*. Two isolates harbored *dfrG*, of which one also harbored *ermC*.

Thirty-nine (26.0% of CC5) isolates harbored a type F IEC (*sep*, *sak*, *chp*, *scn*), of which 35 were ST5 and two were ST7252, a ST5slv. The remaining two isolates were also ST5slvs (ST7262 and ST7263). All isolates harbored *selx* and the *egc*-cluster. Five isolates harbored *sed* + *selj* + *ser*, and two isolates harbored *sec2* + *sel*. Three isolates harbored *tst*. Four isolates harbored *blaZ*, one isolate *ermB* and *tet*(M), and one isolate *ermC*.

All ten type G IEC (*sep*, *sak*, *scn*) CC5 isolates were ST5 and harbored *sep*, *selx*, and the *egc*-cluster. Single isolates also harbored *ermC* and *tet*(M), *dfrG*, or *fexA*.

In seven ST5 isolates the IEC was not detected. All harbored *selx* and the *egc*-cluster, and a single isolate harbored *ermC* and *tet*(M).

#### 3.4.3. Clonal Complex 6

The 16 *agr* group I/capsule type 8 CC6 isolates were all ST6 with four closely related *spa* types, predominantly t701 (*n* = 9) and t304 (*n* = 5). Fifteen of the isolates had a type D IEC. The remaining isolate had a type E IEC. All isolates harbored *selx*. Three of the type D IEC isolates harbored the *seb* enterotoxin gene, of which one also harbored *blaZ*.

#### 3.4.4. Clonal Complex 7

The three *agr* group I/capsule type 8 CC7 isolates were all ST7 with two closely related *spa* types, t7234 and t091. The two t7234 isolates had a type B IEC and the t091 isolate a type G IEC. The three isolates harbored *selx*.

#### 3.4.5. Clonal Complex 8

Thirteen of the 14 *agr* group I/capsule type 5 CC8 isolates were ST8. The remaining isolate was ST5234, an ST8slv. Seven closely related *spa* types were identified, with t008 (seven isolates) the most dominant. Based on the IEC type, the 14 isolates could be classified into three closely related strains.

The seven type B IEC isolates were all ST8 and harbored *selx*. Two isolates harbored *sek* + *seq*, one of which also harbored *blaZ*.

The two type D IEC isolates were ST8 and harbored *sea* and *selx*. One isolate harbored *sec2* + *sel*, *sek* + *seq*, and the other harbored *ermC*.

Of the five type E IEC, four were ST8 and one was ST5234. All five isolates harbored *selx*. One isolate harbored *sed* + *selj* + *ser*, and another harbored *seb*.

#### 3.4.6. Clonal Complex 9

The single *agr* group II/capsule type 5 CC9 isolate was ST9 t4812, with a type B IEC. The isolate harbored *selx*, *sel27*, *sel28*, the *egc*-cluster, *eta*, and *blaZ*.

#### 3.4.7. Clonal Complex 12

Fifteen of the 16 *agr* group II/capsule type 8 CC12 isolates were ST12. The ST12slv ST7251 was also identified. Five closely related *spa* types were identified, with t160 (*n* = 8) the most dominant. Based on the IEC, the 16 isolates could be classified into two closely related strains.

The six type B IEC ST12 isolates harbored *seb*, *selx* and *selz*.

The ten type G IEC isolates were ST12 (*n* = 9) and ST7251. All isolates harbored *sep*, *selx* and *selz*. Four isolates harbored *seb*, and one isolate *sec2* + *sel*. In addition to *seb*, one isolate also harbored *blaZ*.

#### 3.4.8. Clonal Complex 15

CC15 contained 41 *agr* group II/capsule type 8 isolates, and included nine STs: ST5 (*n* = 19), ST582 (*n* = 11), ST3911 (*n* = 5), and single isolates of ST333, ST5059, ST7264, ST7273, ST7283, and ST7286. Nineteen closely related *spa* types were identified, with t084 (*n* = 14) the most dominant. All isolates harbored a type C IEC (*chp*, *scn*), of which 40 harbored *selx*. Two isolates harbored *seb*, and another two isolates harbored eta. Single isolates harbored *dfrG*, *ermC* or *fusC*. Twenty of the 41 isolates harbored *blaZ*.

#### 3.4.9. Clonal Complex 20

The three *agr* group I/capsule type 5 CC20 were ST20. Three closely related *spa* types were identified. Two isolates harbored a type B IEC, and for one isolate the IEC was not detected. The *egc*-cluster and *selx* were detected in all three isolates. One isolate also harbored *seb* and *blaZ*.

#### 3.4.10. Clonal Complex 22

The eight *agr* group I/capsule type 5 CC22 isolates harbored a type B IEC, and consisted of three STs and eight closely related *spa* types. Six isolates were ST22, and two isolates were ST22slvs: ST7272 and ST7285. The *egc*-cluster and *selx* were detected in all eight isolates. One isolate also harbored *sec1* + *sel* and *blaZ*. Two isolates harbored *tst*.

#### 3.4.11. Clonal Complex 25

The single *agr* group I/capsule type 5 CC25 isolate was ST7276 t258, with a type B IEC. The isolate harbored the *egc*-cluster, *selx*, *edinB*, and *blaZ*.

#### 3.4.12. Clonal Complex 30

Six of the seven CC30 isolates were *agr* group III/capsule type 8 and consisted of three STs (ST30 [*n* = 2] and the ST30slvs ST34 [*n* = 3] and ST39 [*n* = 1]). Five closely related *spa* types were identified. Based on the IEC, five of the six *agr* group III/capsule type 8 isolates could be classified into two closely related strains.

The four type B IEC isolates harbored the *egc*-cluster. The PVL-positive ST30 also harbored *dfrG*, and the three ST34 isolates harbored *seh*, *tst*, and *blaZ*.

The type E IEC ST39 isolate harbored *sec3* + *sel*, *tst*, *seo*, *ant(9)-la*, and *ermA*.

For one *agr* group III/capsule type 8 isolate, ST30 t012 (ISTOP319), the IEC was not detected. The isolate harbored the *egc* cluster, *tst*, and *blaZ*.

The remaining CC30 isolates was *agr* group III/capsule type 5, and harbored the *egc*-cluster, *seb*, *sel*, *seh*, and *tst*.

#### 3.4.13. Clonal Complex 45

CC45 contained 47 *agr* group I/capsule type 8 isolates and included 10 STs: ST45 (*n* = 30); the ST45slvs ST508 (*n* = 5) and single isolates of ST7254, ST7255, ST7261, ST7268, ST7284, and ST7289; and two ST45dlvs, ST7258 and ST7279. Twenty-five closely related *spa* types were identified, with t015 (*n* = 11) the most dominant. Based on the IEC type, the 47 isolates could be classified into three closely related strains.

Of the 45 type B CC45 isolates, all harbored *selx* and 44 harbored the *egc*-cluster. Twenty-seven isolates harbored *sec* + *sel*, (19 *sec2* + *sel* and 8 *sec3* + *sel*), of which ten harbored *tst*. One isolate harbored *seb*. A single isolate harbored multiple AMR genes, including *ant(4′)-Ia*, *aadD*, *ermC*, and *mphC*. Two isolates harbored *blaZ*.

The type C IEC ST45 isolate harbored *selx* and the *egc*-cluster.

The type E IEC ST45 isolate harbored *selx*, the *egc*-cluster, and *sec2* + *sel*.

#### 3.4.14. Clonal Complex 59

CC59 contained nine *agr* group I/capsule type 8 isolates, and included four STs: ST59 (*n* = 6) and single isolates of the ST59slvs ST87, ST1224, and ST7280. Five closely related *spa* types were identified. Based on the IEC type, the isolates could be classified into four closely related strains.

The type A IEC ST7280 isolate harbored *sea*, *seb*, *sek* + *seq*, *selx*, and *sely*.

The three type B IEC CC59 isolates consisted of two ST59 isolates and the ST87 isolate. The three isolates harbored *selx* and *sely*. Two isolates also harbored *seb*, *sek* + *seq*, and the third isolate harbored *ant(9)-Ia* and *ermA*.

The four type C IEC CC59 isolates consisted of three ST59 isolates and the ST1224 isolate. The four isolates harbored *selx* and *sely*. One isolate also harbored *seb* and *sek* + *seq*.

The type D IEC ST59 isolate harbored *selx*, *sely*, *seb*, and *sek* + *seq*.

#### 3.4.15. Clonal Complex 80

The single *agr* group III/capsule type 8 ST80 t042 had a type B IEC. The isolate harbored *seb*, *seh*, *sek* + *seq*, *selx*, *sely*, and *edinB*.

#### 3.4.16. Clonal Complex 88

Of the 17 CC88 isolates, 16 were *agr* group III/capsule type 8 isolates with a type E IEC. The 16 isolates included 3 STs: ST88 (*n* = 9) and the ST88slvs ST78 (*n* = 6) and a single isolate of ST7254. Thirteen closely related *spa* types were identified. All isolates harbored *selx*. One isolate harbored *lukS/F-PV*. Five isolates harbored *ant(9)-Ia* and *ermA*, of which two harbored *sec2* + *sel*.

The IEC was not detected in one *agr* group III/capsule type 8 ST78 isolate. The isolate harbored *selx* and *sec2* + *sel*.

#### 3.4.17. Clonal Complex 97

The 48 type E *agr* group I/capsule type 5 CC97 isolates consisted of five STs: ST97 (*n* = 34), the ST97slvs ST953 (*n* = 10), ST1179 (*n* = 2), and ST7256 (*n* = 1), and the ST97dlv ST7278. Nineteen closely related *spa* types were identified, with t267 (*n* = 21) the most dominant. All isolates harbored *selx*. Two isolates harbored *sec3* + *sel* and *tst*. Single isolates harbored *ant(9)-Ia* and *ermA*, or *tet*(K), or *lnuA*.

#### 3.4.18. Clonal Complex 101

The 16 type E IEC *agr* group I/capsule type 8 CC101 isolates consisted of four STs: ST101 (*n* = 12), the ST101slvs ST1155 (*n* = 2) and ST7257 (*n* = 1), and ST7274 (*n* = 1). Seven *spa* types were identified, with t528 (*n* = 9) the most dominant. All isolates harbored *selx*. Two isolates harbored *sec2* + *sel*, and one isolate harbored *blaZ*.

#### 3.4.19. Clonal Complex 188

CC188, contained 23 *agr* group I/capsule type 8 isolates, and included three STs: ST188 (*n* = 21), and single isolates of the ST188slvs ST7259 and ST7271. Four closely related *spa* types were identified, with t189 (*n* = 19) the most dominant. Based on the IEC type, the isolates could be classified into three closely related strains.

The 11 type B IEC CC188 isolates consisted of ten ST188 isolates and the ST7259 isolate. All isolates harbored *selx*.

The eight type E IEC CC188 isolates were all ST188 and harbored *selx*. Individual isolates harbored either *seb*, *aph(3′)-IIIa*, or *blaZ*.

The four type G IEC CC188 isolates consisted of three ST188 isolates and the ST7271 isolate. All isolates harbored *sep* and *selx.*

#### 3.4.20. Clonal Complex 291

The four *agr* group I/capsule type 5 CC291 isolates were identified as ST291 (*n* = 3) and as ST7287, an ST291slv. Three closely related *spa* types were identified. Three isolates had a type B IEC, with the remaining isolate having a type A IEC. The four isolates harbored *edinB*.

#### 3.4.21. Clonal Complex 361

The five *agr* group I/capsule type 8 ST672 isolates had either a type B (*n* = 1) or type E (*n* = 4) IEC. Four closely related *spa* types were identified. The five isolates harbored *selx* and the *egc*-cluster. One of the type E isolates also harbored *see*.

#### 3.4.22. Clonal Complex 398

CC398 contained 16 *agr* group I/capsule type 5 isolates and included four STs: ST398 (*n* = 12), and the ST398slvs ST3332 (*n* = 2), ST7275 (*n* = 1), and ST7277 (*n* = 1). Six closely related *spa* types were identified, with t1451 (*n* = 19) the most dominant. The *spa* type could not be determined for one isolate. Three IEC types were identified, including types C (*n* = 13), B (*n* = 2), and E (*n* = 1). All type C IEC isolates harbored either *ermT* (*n* = 12) or *ermC* (*n* = 1).

#### 3.4.23. Singletons

The seven isolates with STs not able to be grouped into a CC included:

- Three *agr* group II/capsule type 5 ST2867 isolates with three closely related *spa* types. Two of the three isolates harbored a type E IEC, while in one isolate the IEC was not detected. The three isolates harbored *selx* and *edinB*, and one type E IEC isolate harbored *ant(4’)-Ia* and *aadD*.
- One *agr* group II/capsule type 5 ST425 isolate with a type B IEC harboring *selx*.
- One *agr* group II/capsule type 5 ST573 t1839 isolate with an unidentified IEC (only *scn* identified) harboring *selx*, *selz*, *sel27*, *sel28*, the *egc*-cluster, and *sec2* + *sel*.
- One *agr* group III/capsule type 8 ST5491 t5925 isolate with a type E IEC harboring *selx*.
- One *agr* group I/capsule type 5 ST7270 isolate with a type G IEC harboring *selx* and *seb*.

### 3.5. Phylogenetic Analyses of PSSA Isolates

**Figure 2:**
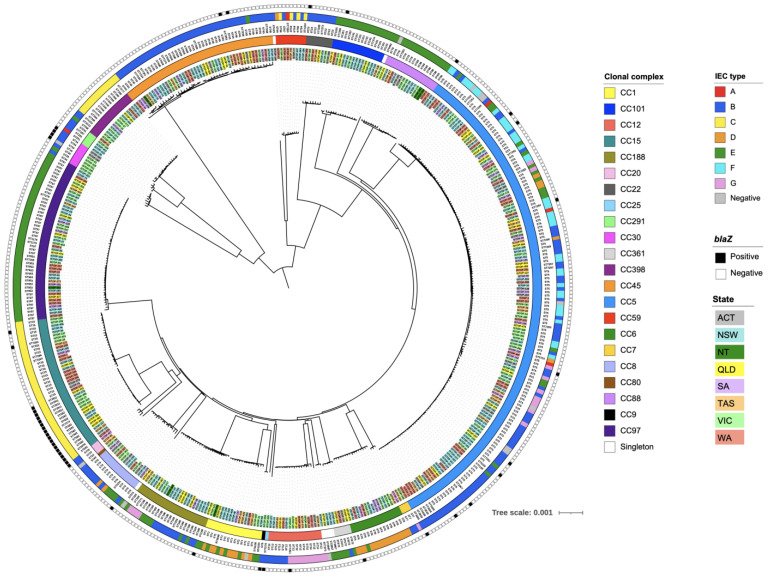
Phylogenetic tree of 470 MSSA isolates causing bacteremia in Australia during 2020 classified as penicillin-susceptible by Vitek^®^ 2. The branch tips are labelled with the sample ID. The branch labels are highlighted in different colors, each representing the state or territory from which the isolates were collected. Data on the clonal complex (inner circle), sequence type (labels), IEC type (outer circle), and presence of the *blaZ* gene (square symbols) are overlaid on the tree. CC, clonal complex; ST, sequence type; IEC, immune evasion cluster; ACT, Australian Capital Territory; NSW, New South Wales; NT, Northern Territory; QLD, Queensland; SA, South Australia; TAS, Tasmania; VIC, Victoria; WA, Western Australia. The tree was constructed using single nucleotide polymorphisms from the core genome alignment of the PSSA genomes.

A phylogenetic tree was constructed to explore the relationship among the *blaZ*-positive and *blaZ*-negative PSSA isolates (Figure 2). The PSSA clones identified were collected in more than one region (state or territory). Most CCs were predominantly represented by one clade, except CC15 and CC88, which were each represented by two phylogenetically distinct clades. For CC15, one clade contained ST15, ST33, ST7264, ST7283, and ST7286 isolates, while the other contained ST582, ST3911, ST5059, and ST7273 isolates. For CC88, one clade contained ST88 isolates, while the other contained ST78 and ST7281 isolates.

The immune evasion cluster (IEC) was detected in 457 (97.2%) isolates. More than one IEC type was identified in many CCs. Type B (*sak*, *chp* and *scn*) and type E (*sak* and *scn*) were the predominant IEC types, identified in 36.0% (*n* = 169) and 27.0% (*n* = 127) of isolates, respectively. IEC type C (*chp*, and *scn*), F (*sep*, *sak*, *chp* and *scn*), D (*sea*, *sak*, and *scn*), G (*sep*, *sak*, and *scn*), and A (*sea*, *sak*, *chp* and *scn*) were identified in 12.6% (*n* = 59), 8.3% (*n* = 39), 6.8% (*n* = 32), 5.5% (*n* = 26), and 1.1% (*n* = 5) of isolates, respectively. One isolate, ISTOP-483 (ST573), harbored a single IEC-associated gene, *scn*, and was not able to be designated with an IEC type. IEC-associated genes were not detected in 12 isolates, including seven ST5 isolates and single isolates of ST1, ST20, ST30, ST78 and ST2867.

Type A IEC was identified in five isolates which belonged to CC5, CC59, and CC291. Type B IEC was identified in all CCs except CC1, CC6, CC15, CC88, CC97, and CC101. All isolates from CC9, CC22, CC25, and CC80 harbored a type B IEC. Type C IEC was identified in CC15, CC45, CC59, and CC398. All isolates from CC15 harbored a type C IEC. Type D IEC was identified in CC1, CC5, CC6, CC8, and CC59. Type E IEC was identified in CC1, CC5, CC6, CC8, CC30, CC45, CC88, CC97, CC101, CC188, CC361, and CC398. All isolates from CC97 and CC101 harbored a type E IEC. Type G IEC was identified in CC12, CC5, CC7, and CC188. In this study, eight CCs harbored only one IEC type: CC9 (type B), CC15 (type C), CC22 (type B), CC25 (type B), CC80 (type B), CC88 (type E), CC97 (type E), and CC101 (type E).

### 3.6. Phenotypic Antimicrobial Susceptibility Testing of blaZ-Positive Isolates Classified as Penicillin-Susceptible by Vitek^®^ 2

Using WGS, the *blaZ* gene was detected in 9.6% (*n* = 45) of the 470 isolates identified as penicillin-susceptible by Vitek^®^ 2. Penicillin susceptibility tests (disc diffusion, broth microdilution (BMD), Etest^®^, and nitrocefin) were performed on the *blaZ*-positive isolates; the results are summarized in Table 3.

The BMD MICs ranged from 0.03 mg/L to 16 mg/L, with only 13 isolates classified as penicillin-resistant (MIC > 0.12 mg/L). The MICs of the 32 isolates classified as penicillin-susceptible were close to the resistance, breakpoint including 14 and 10 isolates with an MIC of 0.125 mg/L and 0.06 mg/L, respectively. Eight isolates had an MIC of 0.03 mg/L.

The performance of the Etest^®^ was similar to the BMD, with only 12 isolates classified as penicillin-resistant. The MICs of the 33 isolates classified as penicillin-susceptible ranged from 0.023 mg/L to 0.125 mg/L, with 25 isolates having an MIC close to the resistance breakpoint.

Although the EUCAST disc diffusion method identified 82.2% of the *blaZ*-positive resistant isolates whilst the CLSI disc diffusion method only identified 57.8%, both methods detected 100% of the isolates when the zone edge appearance was considered in determining penicillin resistance.

Approximately 50% of *blaZ*-positive isolates were nitrocefin positive.

### 3.7. Genotypic Characterisation of blaZ-Positive Isolates Classified as Penicillin-Susceptible by Vitek^®^ 2

The 45 *blaZ*-positive isolates were from 13 different clonal lineages. We identified at least two isolates from CC15 (*n* = 20), CC5 (*n* = 10), CC30 (*n* = 4), and CC45 (*n* = 2), and one isolate from each of the following CCs: CC6, CC8, CC9, CC12, CC20, CC22, CC25, CC101, and CC188 (Figure 2). The single *blaZ*-positive isolates from CC9 and from CC25 were the only representatives of these CCs in our study. The CC15 *blaZ*-positive isolates represented by ST582, ST3911, ST5059, and ST7273 formed a distinct cluster, and were genetically distant from other CC15 isolates. The CC30 *blaZ*-positive isolates also formed a cluster, and were genetically distant from the CC30 *blaZ*-negative isolates. The distribution of the remaining *blaZ*-positive isolates was scattered, and clonal expansion of *blaZ*-positive isolates was not identified in the other clonal lineages.

**Figure 3:**
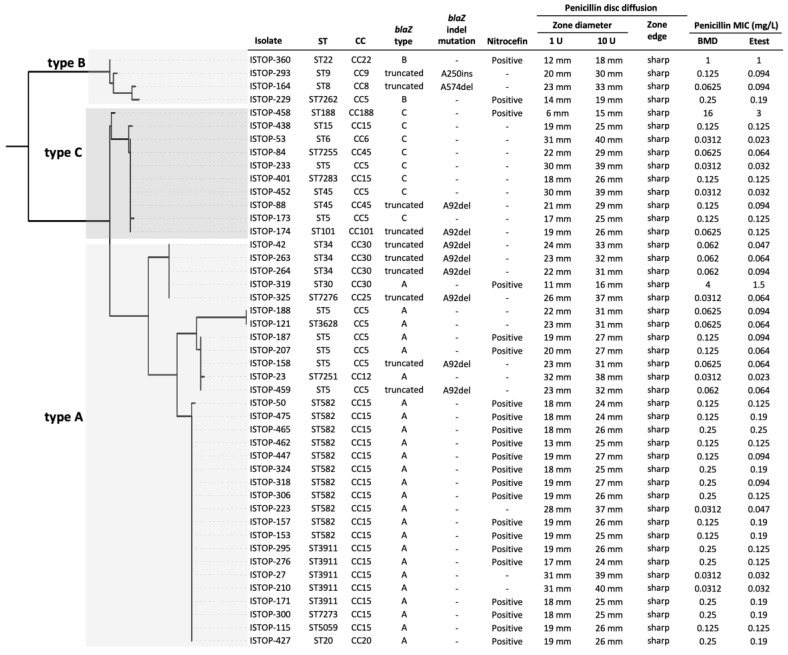
Phylogeny of *blaZ* nucleotide sequences and characteristics of the *blaZ*-positive isolates. The BlaZ type (A, B, C) was defined by amino acids encoded at positions 119 and 207. A truncated *blaZ* type was defined by the presence of a premature stop codon. ST, sequence type; CC, clonal complex; BMD, broth microdilution; A92del, deletion of an adenine at nucleotide position 92; A574del, deletion of an adenine at nucleotide position 574; A250ins, insertion of an adenine at nucleotide position 250.

The *blaZ*, *blaR1*, and *blaI* genes were intact in 78%, 58%, and 100% of *blaZ*-positive isolates, respectively. The *blaZ* gene was truncated in ten isolates and harbored a frameshift indel mutation, resulting in premature termination of the encoded protein. The indel mutations included the deletion of an adenine at nucleotide position 92 (from a string of nine adenines) in eight isolates or at nucleotide position 574 (from a string of eight adenine) in one isolate or the insertion of an adenine at nucleotide position 250 (from a string of six adenines) in the remaining isolate. All *blaZ*-positive isolates from CC8, CC9, CC25, and CC101 harbored a truncated *blaZ*. All isolates harboring a truncated *blaZ* were categorized as susceptible by BMD (0.031–0.125 mg/L) and Etest^®^ (0.047–0.125 mg/L). Type A *blaZ* was identified in 71% (*n* = 25) of isolates harboring an intact *blaZ*, while the remainder harbored type C *blaZ* (*n* = 8) or type B *blaZ* (*n* = 2) (Figure 3).

A truncated *blaR1* was identified in 25 isolates harboring an intact *blaZ*. The deletion of an adenine at nucleotide position 466 (from a string of 8 adenines) was identified in 23 isolates and the insertion of an adenine at nucleotide position 221 was identified in one isolate. The remaining isolate harbored a stop codon polymorphism at codon position 244. Of the isolates harboring a truncated blaR1, 64% (*n* = 16) and 80% (*n* = 20) were penicillin-susceptible per the BMD and Etest^®^, respectively. However, the remaining isolates had an MIC close to the breakpoint (0.19–0.25 mg/L).

Ten *blaZ*-positive isolates harbored an intact *blaZ*, *blaR1*, and *blaI*, of which six were penicillin-susceptible and four were penicillin-resistant per BMD and Etest^®^. The penicillin-susceptible isolates had no changes in the deduced amino acid sequence of BlaR1 and BlaI when compared to the sequence of *S. aureus* ATCC^®^ 29213. However, at least two amino acid changes were identified in the *blaZ* sequence from a total of 14 amino acid changes (A9V, I10M, P22S, E26K, V77I, I86V, G145E, E193K, K196N, M203F, S207N, S217P, C220Y, and G245V. The four penicillin-resistant isolates harbored at least six amino acid changes in *blaZ* from a total of 30 amino acid changes (F6L, A9V, N20T, P22S, D29N, H37N, L44N, S55A, I86V, T109A, K112A, T119K, V139I, Q141K, G145E/K/*n*, E193K, K196N, M203F, S207N, S217P, C220Y, V241I, G245N, V251I, S276N, M278I, K279N, and E280K), and three isolates harbored at least one amino acid change in *blaI* from a total of six amino acid changes (D21G/*n*, Y37N, S63Y, E64N, and N72I) (Supplementary Table S4).

## 4. Discussion

*S. aureus*, which historically has been defined by the emergence of antibiotic-resistant strains, is included in the WHO’s list of twelve global priority bacterial species with critical, high, and medium antibiotic resistance. However, recent peer-reviewed scientific publications have reported a global rise in the prevalence of PSSA bloodstream infections [7,9,20,35]. The prevalence of PSSA bloodstream infections has also increased in Australia, where the proportion of methicillin-susceptible *S. aureus* causing bacteremia identified phenotypically as penicillin-susceptible has increased by over 35%, from 17.5% in 2013 to 23.7% in 2020 [11,36]. Despite the increase in susceptibility, benzylpenicillin is still not routinely used for treating PSSA bacteremia. The optimal treatment for PSSA bacteremia remains unknown, and concerns about the clinical laboratory’s ability to reliably detect penicillinase-producing strains by traditional phenotypic methods means benzylpenicillin is not recommended, despite evidence of better patient outcomes in PSSA infections. As clinical outcomes may be better with benzylpenicillin due to its better pharmacokinetic and pharmacodynamic properties and better adverse event profile when compared to flucloxacillin, the re-emergence of PSSA warrants investigation to determine whether it offers a novel antimicrobial stewardship possibility.

Using WGS to determine the genetic lineages, we have shown that the emergence of penicillin-susceptible SAB in Australia is not due to the expansion of a single clone. Using a collection of 470 Vitek^®^ 2 PSSA from the 2020 AGAR ASSOP, we identified 84 STs, of which 79 could be grouped into 22 CCs. Although polyclonal, 65.7% of isolates could be grouped into five CCs, (CC5, CC15 CC45, CC97, CC188), with 150 (31.9%) isolates classified as CC5. Many of the CCs had multiple STs and *spa* types, and based on the IEC type, isolates within a CC could be classified into different strains harboring a range of virulence and resistance genes. However phylogenetic analyses of the isolates showed that most CCs were represented by one clade, with only CC15 and CC88 having two phylogenetically distinct clades.

Although a variety of antimicrobial resistance genes and mutations associated with quinolone, rifampicin, and fusidic acid resistance were detected, 77.2% (363 isolates) of PSSA were pan-susceptible. The *blaZ* gene was the most frequently identified resistance gene, detected in 9.6% of PSSA. Isolated across Australia, the 45 *blaZ*-positive PSSA were identified in 13 different CCs. Almost half of the isolates were from CC15, and were found to be genetically distant from the *blaZ*-negative CC15 PSSA.

Overall, 83.4% (392 isolates) and 16.6% (78 isolates) of the 470 PSSA episodes of bacteremia were classified as community-onset and hospital-onset, respectively. Fifteen (33.3%) of the 45 *blaZ*-positive PSSA episodes were hospital-onset compared, to 63 (14.8%) of the 425 *blaZ*-negative PSSA episodes. The proportion of hospital-onset *blaZ*-positive PSSA episodes was significantly higher than the proportion of hospital-onset *blaZ*-negative PSSA episodes (*p* < 0.01), suggesting that *blaZ*-positive PSSA is more likely to be linked with hospital-associated *S. aureus* clones than with community-associated clones.

Very few studies have investigated the population structure of PSSA, and as far as we are aware, only one other study has used WGS to investigate the genomic epidemiology and characterisation of PSSA [10]. In 2014, Resman et al. investigated the prevalence and population structure of PSSA bloodstream isolates in Malmö, Sweden [7]. The study was performed over a 12-month period in a geographical area of limited size (population of approximately 500,000), and characterisation of isolates was performed solely by single gene (*spa*) typing. Consequently, detailed interpretation of the PSSA population structure and its clonal distribution was not possible. Of the 257 *S. aureus* included in the study, 85 were PSSA (33.1%), of which five (5.9%) were *blaZ*-positive. Although the *blaZ*-negative PSSA isolates were scattered across several CCs, approximately 50% of the isolates were CC5 and CC45. Resistance to non-beta lactam antibiotics was rare among the isolates.

In 2021, Mama et al. investigated PSSA isolated from blood cultures collected over a period of 6–12 months in sixteen Spanish hospitals [20]. Of the 754 methicillin-susceptible *S. aureus* included in the study, 156 were penicillin-susceptible (20.7%), of which five (3.2%) were *blaZ*-positive. Similar to the Resman et al. study, characterisation of isolates was primarily performed by *spa* typing, with multilocus sequencing typing performed on selected isolates. Different clonal lineages were identified amongst the *blaZ*-negative PSSA, with the most prevalent being CC5 (23.2%), CC398 (16.6%), and CC45 (15.9%). Overall, 77.5% of PSSA were pan-susceptible, and only four isolates were PVL-positive.

In 2022, Jin et al. investigated PSSA bloodstream isolates collected over six years in 55 hospitals across 37 cities in 17 Chinese provinces and reported that PSSA had increased significantly, from 3.5% in 2014 to 22.1% in 2019 [10]. Of the 1952 methicillin-susceptible *S. aureus* included in the study, 140 were penicillin-susceptible (7.2%), of which 11 (7.9%) were *blaZ*-positive. WGS was performed on the PSSA, and phylogenetic analysis showed high strain diversity, with the most frequent CCs identified being CC188 (17.1%), CC398 (15.7%), CC5 (15.7%), CC8 (9.3%), and CC1 (7.1%). Most PSSA (58.1%) were pan-susceptible, and only a small number of isolates PVL-positive.

These four studies have reported the population structure of PSSA to be diverse and, though some of the predominant CCs are shared between countries (China, Europe and Australia (CC5), Europe and Australia (CC45), and China and Australia (CC188)), CC15 and CC97 were only dominant in Australia. In the Chinese study, approximately 50% of *blaZ*-positive PSSA were CC188, while in Australia CC15 dominated. Across all four studies, PSSA was typically pan-susceptible and PVL-negative. However, as demonstrated by Jin et al., it should not be assumed that PSSA is less virulent that PRSA in vivo.

Similar to the Spanish and Chinese PSSA studies, our *blaZ*-positive PSSA harbored *blaZ* types A, B, or C. Identification of the same *blaZ* sequences in different STs (and CCs) among our *blaZ*-positive isolates suggests exchange of *blaZ* via horizontal gene transfer. The most common *blaZ* indel mutation (A92del), identified in eight *blaZ*-positive PSSA, was also detected in three isolates from the Jin et al. study. Similarly, the most common blaR1 indel mutation (A466del), identified in 24 of our *blaZ*-positive PSSA, was also detected in seven isolates from the Jin et al. study. These observations confirm A92del and A466del as mutation hotspots for *blaZ* and blaR1, respectively. As both mutations exist within a short 6–8 adenine stretch, they likely arose from slipped strand mispairing [37]. Further work is required to investigate whether these mutations are reversible, which would result in the expression of a functional BlaZ. Non-synonymous mutations were observed in *blaZ* as well as *blaI*-, and these mutations should be investigated further.

Different studies have questioned the reliability of phenotypic susceptibility testing to detect PRSA. In a recent study, Skov et al. demonstrated that although phenotypic testing could reliably detect *blaZ*-negative PSSA, detection of *blaZ*-positive PSSA proved troublesome [38]. BMD, CLSI disc diffusion, and cefinase testing could only identify 49%, 51%, and 78% of *blaZ*-positive PSSA, respectively. Although 93% of *blaZ*-positive PSSA were detected by EUCAST disc diffusion, when the interpretation of the zone edge appearance was used both the EUCAST and CLSI disc diffusion methods detected 96% of isolates. In our study, we found that the Etest^®^ and BMD performed poorly, with only 26.7% and 28.8% of *blaZ*-positive isolates classified as penicillin-resistant, respectively. Similarly, the nitrocefin test correctly classified less than half of the isolates. As in the Skov et al. study, we found that the EUCAST disc diffusion method was superior to the CLSI disc diffusion method. However, when the zone edge appearance was taken into consideration, both methods detected 100% of the *blaZ*-positive PSSA.

## 5. Conclusions

We have shown that PSSA bacteremia in Australia is due to multiple lineages. Although diverse, the dominant PSSA CCs were CC5, CC15, CC45, CC97, and CC188. Approximately 10% of *S. aureus* classified as penicillin-susceptible by the Vitek^®^ 2 automicrobic system harbored *blaZ*, with approximately 50% of the isolates belonging to CC15. Consequently, we recommend *S. aureus* classified as penicillin-susceptible by Vitek^®^ 2 should be confirmed by an alternative method. Although we found that the disc diffusion method detected all *blaZ*-positive PSSA in our study, the interpretation of the zone edge appearance is subjective, and therefore a *blaZ* PCR assay may be a better alternative. A reliable early screening method for the detection of PSSA infections should enable the tailoring of antibiotic regimens to ensure better patient outcomes.

## Figures and Tables

**Table 1:** The prevalence of virulence genes detected by whole genome sequencing in 470 penicillin-susceptible *Staphylococcus aureus* with different clonal clusters.

Clone	N	PVL	*tst*	*edinB*	*eta*	*sea*	*seb*	*sec + sel*	*sed + selj + ser*	*sek + seq*	*sep*	*see*	*seh*	*egc*- cluster	*selx*	*sely*	*selz*	*sel28*	*sel31*	*sel32*
CC1	17	1			1	9	1	1		14			15		17					
CC5	150		3			8	1	2	17		49			150	150				1	1
CC6	16					15	3								16					
CC7	3										1				3					
CC8	14					2	1	1	1	4					14					
CC9	1				1									1	1	1		1		
CC12	16						10	1			10				16		16			
CC15	41				2		2								40					
CC20	3						1							3	3	3				
CC22	8		2					1						8	8					
CC25	1			1										1	1					
CC30	7	1	6				1	2					4	6						
CC45	47		10				1	28						46	47					
CC59	9					2	5			5					9	9				
CC80	1			1			1			1			1		1	1				
CC88	17	1						3							17					
CC97	48		2					2							48					
CC101	16							2							16					
CC188	23						1				4				23					
CC291	4			4		1														
CC361	5											1		5	5					
CC398	16																			
ST425	1														1					
ST573	1							1						1	1		1	1		
ST2867	3			3											3					
ST5491	1														1					
ST7270	1						1				1				1					
Total	470	3	23	9	4	37	29	44	18	24	65	1	20	221	442	14	17	2	1	1

**Table 2:** The prevalence of antimicrobial resistance genes detected by whole genome sequencing in 470 penicillin-susceptible *Staphylococcus aureus* with different clonal clusters.

Clone	N	*blaZ*	*ant(4′)-Ia*	*aadD*	*ant(9)-Ia*	*aph(3′)-IIIa*	*dfrG*	*ermA*	*ermB*	*ermC*	*ermT*	*fexA*	*fusC*	*lnuA*	*mphC*	*tet*(K)	*tet*(M)	*mdfA*
CC1	17												12					
CC5	150	10			1		4	1	1	8		2				1	6	1
CC6	16	1																
CC7	3																	
CC8	14	1								1								
CC9	1	1																
CC12	16	1																
CC15	41	20					1			1			1					
CC20	3	1																
CC22	8	1																
CC25	1	1																
CC30	7	4			1		1	1		1								
CC45	47	2	1	1						1					1			
CC59	9				1			1										
CC80	1																	
CC88	17				5			5										
CC97	48				1			1						1		1		
CC101	16	1																
CC188	23	1				1												
CC291	4																	
CC361	5																	
CC398	16									1	12							
ST425	1																	
ST573	1																	
ST2867	3		1	1														
ST5491	1																	
ST7270	1																	
Total	470	45	2	2	9	1	6	9	1	13	12	2	13	1	1	2	6	1

**Table 3:** Penicillin susceptibility and nitrocefin results of 45 *blaZ*-positive Vitek^®^ 2 penicillin-susceptible *S. aureus*.

Method	Criteria	Interpretive Guidelines	Resistance	Resistant *n* (%)	Sensitive *n* (%)
Disc Diffusion	Zone Diameter	EUCAST P1	<26 mm	37 (82.2)	8 (17.8)
		CLSI P10	<29 mm	26 (57.8)	19 (42.2)
Disc Diffusion	Zone Edge	EUCAST P1	Sharp Zone Edge	45 (100)	0 (0)
		CLSI P10	Sharp Zone Edge	45 (100)	0 (0)
Broth Microdilution	Breakpoint	EUCAST	>0.125 mg/L	13 (28.8)	32 (71.1)
		CLSI	>0.125 mg/L		
Etest^®^	Breakpoint	EUCAST	>0.125 mg/L	12 (26.7)	33 (73.3)
		CLSI	>0.125 mg/L		
				**Positive**	**Negative**
Nitrocefin Test	Colour Change		Red	22 (48.9)	23 (51.1)

## Data Availability

Not applicable.

## Supplementary Materials

The following supporting information can be downloaded at: https://www.mdpi.com/article/10.3390/microorganisms10081650/s1, Table S1: Origin, onset, multi-locus sequence type, clonal cluster, *spa* type, Vitek^®^ 2 penicillin minimum inhibitory concentration and detection of *blaZ* in 470 penicillin-susceptible *Staphylococcus aureus* identified in the Australian Group for Antimicrobial Resistance’s 2020 Australian *Staphylococcus aureus* Sepsis Outcome Program; Table S2: Multilocus sequence type, origin, Vitek^®^ 2 penicillin minimum inhibitory concentration, detection of *blaZ*, antibiogram and resistance gene profile of 470 penicillin-susceptible *Staphylococcus aureus* identified in the Australian Group for Antimicrobial Resistance’s 2020 Australian *Staphylococcus aureus* Sepsis Outcome Program; Table S3: Multilocus sequence type, origin, Vitek^®^ 2 penicillin minimum inhibitory concentration, detection of *blaZ*, *agr* type, capsule type, *spa* type and virulence genes in 470 penicillin-susceptible *Staphylococcus aureus* identified in the Australian Group for Antimicrobial Resistance’s 2020 Australian *Staphylococcus aureus* Sepsis Outcome Program; Table S4: BlaZ allotypes and BlaZ, BlaR1 and BlaI mutations identified in 45 *blaZ*- positive PSSA.
