# Supplementary material for: Molecular Epidemiology of Penicillin-Susceptible Staphylococcus aureus Bacteremia in Australia and Reliability of Diagnostic Phenotypic Susceptibility Methods to Detect Penicillin Susceptibility
Source: Microorganisms. 2022 Aug 15;10(8):1650. doi: 10.3390/microorganisms10081650 (PMC9413241; doi:10.3390/microorganisms10081650)
Supplement: Supplementary file 1 [file microorganisms-10-01650-s001.zip › Supplementary Table 1.pdf]

Table S1: Origin, onset, multilocus sequence type, clonal cluster, *spa* type, Vitek® 2 penicillin minimum inhibitory concentration and detection of blaZ on 470 penicillin-susceptible *Staphylococcus aureus* identified in the Australian Group for Antimicrobial Resistance's 2020 Australian *Staphylococcus aureus* Sepsis Outcome Program

Isolate	STATE	ONSET	MLST	ST	CC	spa	Spa Type	Vitek® 2 Pen mg/L	blaZ
ISTOP-1	NSW	Community	12-4-1-4-12-1-3	ST6	6	11-10-21-17-34-24-34-22-25	t304	0.06	ND
ISTOP-2	NSW	Hospital	3-1-1-8-1-1-1	ST188	188	07-23-12-21-17-34	t189	0.06	ND
ISTOP-3	WA	Community	1-4-1-4-12-1-10	ST5	5	26-23-17-34-17-20-17-12-17-16	t002	0.06	ND
ISTOP-4	WA	Hospital	1-4-374-4-12-1-10	ST2967	5	26-23-17-12-12-16	t1345	≤0.03	ND
ISTOP-5	WA	Community	144-1-1-1-1-5-3	ST1179	97	07-34-34-33-34	t237	0.12	ND
ISTOP-6	WA	Community	3-779-1-1-4-4-3	ST5234	8	11-19-12-21-17-34-24-34-22-33-25	t955	0.06	ND
ISTOP-7	WA	Hospital	13-13-1-1-12-11-13	ST15	15	07-23	t605	0.06	ND
ISTOP-8	WA	Community	22-1-14-23-12-4-31	ST88	88	26-12-21-17-21-17-34-34-34-33-34	UD	0.12	ND
ISTOP-9	WA	Community	1-4-1-4-12-1-10	ST5	5	26-17-20-17-12-17-17	t1227	≤0.03	ND
ISTOP-10	WA	Community	3-1-122-1-1-5-3	ST953	97	07-23-12-21-17-34-34-34-33-34	t267	0.12	ND
ISTOP-11	WA	Community	3-35-19-2-20-26-29	ST398	398	08-12-16-34-24-25	t2928	0.06	ND
ISTOP-12	WA	Community	1-3-1-8-11-5-11	ST12	12	07-23-24-33-22-22-17	UD	≤0.03	ND
ISTOP-13	WA	Community	1-4-463-4-12-1-10	ST3628	5	26-23-17-34-17-12-16	t5259	0.06	ND
ISTOP-14	WA	Community	1-3-1-8-11-5-11	ST12	12	07-23-21-24-33-22-17	t160	0.06	ND
ISTOP-15	VIC	Community	1-4-1-4-12-1-10	ST5	5	26-17-34-17-20-17-17-16	t1560	0.06	ND
ISTOP-16	VIC	Community	3-35-19-2-20-26-29	ST398	398	08-16-02-25-34-25	t1451	0.06	ND
ISTOP-17	VIC	Community	3-3-1-1-4-4-3	ST8	8	11-12-21-17-34-24-34-22-25	t024	0.12	ND
ISTOP-18	VIC	Hospital	1-4-1-4-12-1-10	ST5	5	26-23-17-34-17-20-17-12-17-16	t002	0.06	ND
ISTOP-19	VIC	Community	1-4-1-4-12-1-10	ST5	5	26-23-17-34-17-20-17-12-17-17-16	t306	0.12	ND
ISTOP-20	VIC	Community	44-13-1-1-12-11-13	ST333	15	07-23-13-34-12-12-23-02-12-23	UD	0.06	ND
ISTOP-21	VIC	Community	100-1-1-8-1-1-1	ST4100	1	07-23-12-21-17-34-16-23-23	UD	≤0.03	ND
ISTOP-22	VIC	Community	1-4-1-4-12-1-10	ST5	5	26-23-17-34-17-20-17-12-12-12-16	t1265	≤0.03	ND
ISTOP-23	VIC	Hospital	1-3-1-8-859-5-11	ST7251	12	07-23-21-24-33-22-17	t160	0.12	**DETECTED**
ISTOP-24	VIC	Community	3-1-1-8-1-1-1	ST188	188	07-23-12-21-17-34	t189	0.12	ND

Isolate	STATE	ONSET	MLST	ST	CC	spa	Spa Type	Vitek® 2 Pen mg/L	blaZ
ISTOP-25	NSW	Hospital	3-1-1-8-1-1-1	ST188	188	07-23-12-21-17-34	t189	≤0.03	ND
ISTOP-26	NSW	Community	10-40-8-6-10-3-2	ST508	45	08-16-xx-16-34-76	UD	≤0.03	ND
ISTOP-27	VIC	Community	13-13-1-1-12-10-530	ST3911	15	07-23-12-34-34-12-12-16-23-02-12-23	t2859	0.06	**DETECTED**
ISTOP-28	VIC	Community	1-4-1-4-12-1-10	ST5	5	26-23-17-34-17-20-17-12-17-16	t002	0.06	ND
ISTOP-29	QLD	Hospital	1-3-1-8-11-5-11	ST12	12	15-22-17	t336	≤0.03	ND
ISTOP-30	QLD	Community	1-3-1-8-11-5-11	ST12	12	07-23-24-33-22-22-17	UD	0.06	ND
ISTOP-31	QLD	Hospital	10-14-8-6-10-3-2	ST45	45	08-16-34	t026	0.06	ND
ISTOP-32	QLD	Hospital	1-4-1-4-12-1-10	ST5	5	26-23-17-34-17-20-17-12-17-16	t002	0.12	ND
ISTOP-33	NSW	Community	1-4-1-4-12-1-10	ST5	5	26-23-17-34-17-20-17-12-17-16	t002	0.06	ND
ISTOP-34	NSW	Community	1-4-463-4-12-1-10	ST3628	5	26-23-17-34-17-20-17-12-12-12-16	t1265	≤0.03	ND
ISTOP-35	NSW	Community	10-40-8-6-10-3-2	ST508	45	08-16-02-16-34-13-17-34-16-34	t015	0.12	ND
ISTOP-36	NSW	Community	1-4-1-4-12-1-10	ST5	5	26-23-17-17-16	t2049	0.06	ND
ISTOP-37	NSW	Community	3-1-1-1-1-5-3	ST97	97	26-23-21-17-34-34-34-33-34	t9432	0.06	ND
ISTOP-38	TAS	Community	1-4-1-4-12-1-10	ST5	5	26-23-17-34-17-20-17-12-17-16	t002	0.12	ND
ISTOP-39	TAS	Community	1-4-463-4-12-1-10	ST3628	5	26-23-17-34-17-20-17-12-12-12-16	t1265	0.12	ND
ISTOP-40	TAS	Community	1-3-1-8-11-5-11	ST12	12	07-23-12-33-17	t909	0.12	ND
ISTOP-41	VIC	Community	1-311-1-4-12-1-10	ST7252	5	26-23-17-34-17-20-17-12-17-17	t686	0.12	ND
ISTOP-42	VIC	Hospital	8-2-2-2-6-3-2	ST34	30	04-24-33-31-12-16-12-33-34	UD	0.06	**DETECTED**
ISTOP-44	NSW	Community	3-1-1-8-1-1-1	ST188	188	07-23-12-21-17-34	t189	0.12	ND
ISTOP-45	NSW	Hospital	3-1-122-1-1-5-3	ST953	97	07-23-12-21-17-34-34-33-34	t359	0.06	ND
ISTOP-46	NSW	Hospital	13-13-1-1-12-11-13	ST15	15	07-23-02-12-23	t803	0.12	ND
ISTOP-47	NSW	Community	22-1-14-23-12-53-31	ST78	88	07-12-21-17-34-34-34-33-34	t1814	0.12	ND
ISTOP-48	NSW	Community	1-1-1-1-1-1-1	ST1	1	07-23-21-16-34-33-13	t127	0.12	ND
ISTOP-49	NSW	Community	1-4-1-4-12-1-10	ST5	5	26-23-17-34-17-20-17-12-16	t548	≤0.03	ND
ISTOP-50	NSW	Community	13-13-1-1-12-10-13	ST582	15	07-23-12-34-34-12-23-02-12-23	t085	0.12	**DETECTED**
ISTOP-51	NSW	Hospital	1-3-1-8-11-5-11	ST12	12	07-23-24-33-22-22-17	UD	0.06	ND
ISTOP-52	NSW	Community	3-1-1-8-1-1-1	ST188	188	07-23-21-17-34	t2883	0.12	ND
ISTOP-53	NSW	Community	12-4-1-4-12-1-3	ST6	6	11-10-21-17-34-24-34-22-25-25	t701	0.12	**DETECTED**

Isolate	STATE	ONSET	MLST	ST	CC	spa	Spa Type	Vitek® 2 Pen mg/L	blaZ
ISTOP-54	NSW	Community	3-1-1-1-1-5-3	ST97	97	07-23-12-21-17-34-34-34-33-34	t267	0.12	ND
ISTOP-55	NSW	Community	3-37-19-2-20-26-32	ST291	291	08-16-34-24-34-17-17-17	t3096	0.12	ND
ISTOP-56	NSW	Community	3-1-1-8-1-1-1	ST188	1	07-23-12-21-17-34	t189	0.06	ND
ISTOP-57	NSW	Community	3-1-1-1-1-5-3	ST97	97	07-23-12-21-17-34-34-33-34	t359	0.06	ND
ISTOP-58	NSW	Community	3-1-122-1-1-5-3	ST953	97	07-33-34	t1109	0.12	ND
ISTOP-59	NSW	Community	13-13-1-1-12-11-13	ST15	15	07-23-12-34-34-12-23-02-12-23	t085	0.06	ND
ISTOP-60	VIC	Community	1-4-1-4-12-1-10	ST5	5	26-23-17-34-17-20-17-12-17-16	t002	0.06	ND
ISTOP-61	VIC	Community	1-311-1-4-12-1-10	ST7252	5	26-23-17-34-17-20-17-12-17-17	t686	0.06	ND
ISTOP-62	VIC	Hospital	1-4-1-4-12-577-10	ST5189	5	26-23-17-34-17-20-17-12-12-12-16	t1265	0.06	ND
ISTOP-63	VIC	Hospital	1-4-1-4-12-1-10	ST5	5	26-23-17-34-17-20-17-12-17-16	t002	0.06	ND
ISTOP-64	NSW	Community	1-4-1-4-12-1-10	ST5	5	26-23-17-34-17-20-17-12-17-16	t002	0.12	ND
ISTOP-65	NSW	Community	3-1-1-1-1-5-3	ST97	97	07-23-12-21-17-13-34-33-34	t14122	0.12	ND
ISTOP-66	NSW	Community	1-4-1-4-12-1-10	ST5	5	26-23-17-34-17-20-17-12-12-12-16	t1265	0.06	ND
ISTOP-67	WA	Hospital	19-23-15-2-19-20-15	ST59	59	04-20-17-20-17-25-34	t437	0.12	ND
ISTOP-68	WA	Community	10-14-8-6-10-3-2	ST45	45	08-16-02-16-34	t230	0.06	ND
ISTOP-69	NSW	Community	100-1-1-8-1-1-1	ST4100	1	Not Determined	-	0.06	ND
ISTOP-70	NSW	Community	10-14-8-6-10-3-2	ST45	45	09-34-34-17-34-16-34	t563	0.06	ND
ISTOP-71	NSW	Community	22-1-14-23-12-4-31	ST88	88	26-12-21-17-13-34-34-33-34	t4013	0.06	ND
ISTOP-72	QLD	Community	69-1-14-15-11-19-3	ST1155	101	04-13-21-12-17-17	t4171	0.12	ND
ISTOP-73	NSW	Hospital	3-1-1-1-1-5-3	ST97	97	07-23-12-21-17-34-34-34-33-34	t267	0.12	ND
ISTOP-74	NSW	Community	1-3-1-8-11-5-11	ST12	12	07-23-21-24-33-22-22-17	t771	0.06	ND
ISTOP-75	NSW	Community	10-14-8-6-10-3-964	ST7254	45	09-34-16-34	t132	≤0.03	ND
ISTOP-76	ACT	Community	22-1-14-23-12-53-31	ST78	88	08-21-17-13-34-34	UD	0.12	ND
ISTOP-77	ACT	Community	19-23-15-2-19-20-15	ST59	59	04-20-17-20-17-31-16-34	t216	0.06	ND
ISTOP-78	ACT	Community	3-3-1-1-4-4-3	ST8	8	11-19-12-21-17-34-24-34-22-25	t008	0.06	ND
ISTOP-79	ACT	Community	13-13-1-1-12-11-13	ST15	15	07-23-12-34-34-12-12-23-02-12-23	t084	0.06	ND
ISTOP-80	NSW	Hospital	3-1-1-1-1-5-3	ST97	97	07-23-12-21-17-34-34-33-34	t359	0.12	ND
ISTOP-81	NSW	Hospital	1-4-1-4-12-1-10	ST5	5	26-23-17-34-17-xx-17-12-17-16	UD	0.12	ND

Isolate	STATE	ONSET	MLST	ST	CC	spa	Spa Type	Vitek® 2 Pen mg/L	blaZ
ISTOP-82	TAS	Community	1-4-1-4-12-1-10	ST5	5	26-23-17-34-17-20-17-12-17-16	t002	≤0.03	ND
ISTOP-83	TAS	Community	1-4-1-4-12-1-10	ST5	5	26-23-17-34-17-20-17-12-12-12-16	t1265	0.06	ND
ISTOP-84	TAS	Community	10-14-8-6-10-3-26	ST7255	45	08-16-02-16-34	t230	0.12	**DETECTED**
ISTOP-85	TAS	Community	4-9-1-8-1-10-8	ST20	20	07-17-21-34-34-22-34	t3277	0.12	ND
ISTOP-86	TAS	Community	1-3-1-8-11-5-11	ST12	12	07-23-21-24-33-22-22-17	t771	0.06	ND
ISTOP-87	TAS	Community	12-4-1-4-12-1-3	ST6	6	11-10-21-17-34-24-34-22-25-25	t701	≤0.03	ND
ISTOP-88	TAS	Community	10-14-8-6-10-3-2	ST45	45	08-16-02-16-34-13-17-34-16-34	t015	0.12	**DETECTED**
ISTOP-89	TAS	Community	1-4-1-4-12-1-10	ST5	5	26-23-17-34-17-20-17-12-12-12-16	t1265	0.06	ND
ISTOP-90	SA	Community	1-4-1-4-12-1-10	ST5	5	26-23-17-34-17-20-17-12-17-16-16	t214	0.12	ND
ISTOP-91	SA	Community	12-4-1-4-12-1-3	ST6	6	11-10-21-17-34-24-34-22-25	t304	0.12	ND
ISTOP-92	SA	Community	3-1-1-8-1-1-1	ST188	188	07-23-12-21-17-34	t189	0.06	ND
ISTOP-93	SA	Community	1-4-1-4-12-1-10	ST5	5	26-23-17-34-17-20-17-12-17-16	t002	0.12	ND
ISTOP-94	SA	Community	10-14-8-6-10-3-2	ST45	45	09-02-16-34-16-34	t371	0.12	ND
ISTOP-95	SA	Community	1-4-1-4-12-1-10	ST5	5	26-23-17-34-17-20-17-12-17-16	t002	0.06	ND
ISTOP-96	VIC	Community	3-1-1-1-1-5-3	ST97	97	07-23-12-21-17-34-34-34-33-34	t267	≤0.03	ND
ISTOP-97	VIC	Community	3-35-19-2-20-26-29	ST398	398	08-16-02-25-34-25	t1451	≤0.03	ND
ISTOP-98	VIC	Community	3-35-19-2-402-26-29	ST3332	398	08-16-02-25-34-24-25	t011	≤0.03	ND
ISTOP-99	VIC	Community	3-1-122-1-1-5-925	ST7256	97	07-23-12-21-17-34-34-33-34	t359	0.06	ND
ISTOP-100	VIC	Community	3-35-19-2-402-26-29	ST3332	398	08-16-02-25-34-24-25	t011	≤0.03	ND
ISTOP-101	VIC	Community	8-2-2-2-6-3-2	ST34	30	04-44-33-31-12-16-34-12-33-34	t1670	≤0.03	ND
ISTOP-102	VIC	Community	1-4-1-4-12-1-10	ST5	5	26-23-17-34-12-12-17-16	t3597	≤0.03	ND
ISTOP-103	SA	Community	1-4-1-4-12-1-10	ST5	5	26-23-17-34-17-13-16	t9394	0.06	ND
ISTOP-104	SA	Community	1-4-166-4-12-1-10	ST3724	5	26-23-17-34-17-16	t688	0.06	ND
ISTOP-105	NSW	Community	12-4-1-4-12-1-3	ST6	6	11-10-21-17-34-24-34-22-25-25	t701	0.12	ND
ISTOP-106	NSW	Community	1-4-1-4-12-1-10	ST5	5	35-17-34-17-20-17-12-16	t2958	0.06	ND
ISTOP-107	NSW	Hospital	1-4-1-4-12-1-10	ST5	5	26-23-17-34-17-20-17-12-17-16	t002	0.12	ND
ISTOP-108	QLD	Community	4-3-1-1-11-72-11	ST672	361	26-22-17-20-17-12-17-16-16	t3841	≤0.03	ND
ISTOP-109	QLD	Community	13-13-1-1-12-11-13	ST15	15	07-23-02-12-23	t803	0.12	ND

Isolate	STATE	ONSET	MLST	ST	CC	spa	Spa Type	Vitek® 2 Pen mg/L	blaZ
ISTOP-110	QLD	Community	1-4-1-4-12-1-10	ST5	5	26-23-17-34-17-20-17-12-17-16	t002	≤0.03	ND
ISTOP-111	QLD	Community	1-4-1-4-12-1-10	ST5	5	26-23-17-16	t111	0.06	ND
ISTOP-112	NSW	Community	3-1-1-8-1-1-1	ST188	188	07-23-12-21-17-34	t189	0.06	ND
ISTOP-113	NSW	Community	1-4-1-4-12-1-10	ST5	5	26-23-17-34-17-20-17-12-12-12-16	t1265	≤0.03	ND
ISTOP-114	NSW	Hospital	12-4-1-4-12-1-3	ST6	6	11-10-21-17-34-24-34-22-25-25	t701	0.12	ND
ISTOP-115	NSW	Hospital	13-13-1-212-12-10-13	ST5059	15	07-23-12-34-23-02-12-23	t4714	0.12	**DETECTED**
ISTOP-116	SA	Community	1-4-1-4-12-1-10	ST5	5	26-23-17-34-17-20-17-12-17-16	t002	0.12	ND
ISTOP-117	WA	Community	1-3-1-8-11-5-11	ST12	12	07-23-21-24-33-22-17	t160	0.06	ND
ISTOP-118	WA	Community	1-4-1-4-12-1-10	ST5	5	26-23-17-34-17-20-17-12-17-16	t002	0.12	ND
ISTOP-119	WA	Community	13-13-1-1-12-11-13	ST15	15	07-23-12-34-34-12-12-23-02-12-23	t084	0.12	ND
ISTOP-120	WA	Community	22-1-14-23-12-4-31	ST88	88	26-12-21-17-21-17-34-34-34-33-34	UD	0.12	ND
ISTOP-121	WA	Community	1-4-463-4-12-1-10	ST3628	5	26-23-17-34-17-20-17-12-12-16	t179	0.12	**DETECTED**
ISTOP-122	WA	Community	10-14-8-6-10-3-2	ST45	45	09-34-13-17-34-16-34	t130	0.06	ND
ISTOP-123	SA	Hospital	10-14-8-6-10-3-2	ST45	45	08-16-02-16-34-13-17-13-16-34	t302	0.12	ND
ISTOP-124	SA	Hospital	1-4-1-4-12-1-10	ST5	5	26-23-17-34-17-20-17-12-17-16	t002	0.12	ND
ISTOP-125	SA	Community	5-4-1-4-4-6-3	ST7	7	07-23-21-17-34-12-23-02-20	t7234	0.12	ND
ISTOP-126	SA	Community	3-1-122-1-1-5-3	ST953	97	07-23-12-21-17-34-34-34-33-34	t267	0.12	ND
ISTOP-127	SA	Hospital	1-1-1-1-1-1-1	ST1	1	26-23-21-16-34-33-13	t177	0.12	ND
ISTOP-128	SA	Community	1-4-1-4-12-1-10	ST5	5	26-23-17-34-17-20-17-12-17-16	t002	0.12	ND
ISTOP-129	NSW	Hospital	13-13-1-1-12-11-13	ST15	15	07-23-34-34-12-23-02-12-23	t2216	0.06	ND
ISTOP-130	NSW	Hospital	1-606-1-1-1-1-1	ST3949	1	07-33-13	t2207	0.12	ND
ISTOP-131	NSW	Community	4-3-1-1-11-72-11	ST672	361	26-17	t2379	0.06	ND
ISTOP-132	NSW	Community	3-35-19-2-20-26-29	ST398	398	08-16-02-25-02-25-25	t1170	0.12	ND
ISTOP-133	NSW	Community	3-1-14-15-860-19-3	ST7257	101	04-13-21-12-297-20-17-12-17-17	UD	0.06	ND
ISTOP-134	NSW	Hospital	13-13-1-1-12-11-13	ST15	15	08-34-34-12-34-12-12-23-02-12-23	t14014	0.12	ND
ISTOP-135	NSW	Community	22-1-14-23-12-4-31	ST88	88	07-12-21-17-13-13-13-34-34-33-34	t2649	0.12	ND
ISTOP-136	TAS	Community	3-1-14-15-11-19-3	ST101	101	4	t528	0.06	ND

Isolate	STATE	ONSET	MLST	ST	CC	spa	Spa Type	Vitek® 2 Pen mg/L	blaZ
ISTOP-137	VIC	Community	13-13-1-1-12-11-13	ST15	15	07-23-12-34-12-12-23	t1877	0.06	ND
ISTOP-138	VIC	Community	3-1-1-1-1-5-3	ST97	97	07-23-12-21-17-34-34-34-33-34	t267	0.06	ND
ISTOP-139	VIC	Community	10-14-8-6-10-3-2	ST45	45	08-16-02-02-16-34	t10588	≤0.03	ND
ISTOP-140	VIC	Community	1-3-1-8-11-5-11	ST12	12	07-23-21-24-33-22-17	t160	0.12	ND
ISTOP-141	VIC	Community	1-1-1-1-1-1-1	ST1	1	07-33-13	t2207	≤0.03	ND
ISTOP-142	SA	Community	1-4-1-4-12-1-10	ST5	5	26-23-17-34-17-16	t688	0.06	ND
ISTOP-143	SA	Community	3-3-1-1-4-4-3	ST8	8	11-12-21-17-34-24-34-22-25	t024	≤0.03	ND
ISTOP-144	ACT	Community	3-3-1-1-4-4-3	ST8	8	563-19-12-21-17-34-24-34-22-25	ND	0.06	ND
ISTOP-145	ACT	Community	1-4-1-4-12-1-10	ST5	5	26-17-34-17-20-17-17-16	t17058	0.06	ND
ISTOP-146	ACT	Community	18-33-6-20-7-50-48	ST425	Singleton	14-44-12-17-23	UD	0.06	ND
ISTOP-147	ACT	Community	850-14-8-6-10-3-965	ST7258	45	08-16-02-16-34-34-17-13-13-17-34-16-13	UD	0.06	ND
ISTOP-148	ACT	Community	1-4-1-4-12-1-10	ST5	5	26-23-17-16-17-20-17-12-17-16	t8241	0.06	ND
ISTOP-149	ACT	Community	10-14-8-6-10-3-2	ST45	45	08-16-02-16-34-13-17-34-16-34	t015	0.06	ND
ISTOP-150	ACT	Community	1-3-1-8-11-5-11	ST12	12	07-23-21-24-33-22-17	t160	0.06	ND
ISTOP-151	ACT	Community	4-3-1-1-11-72-11	ST672	361	26-22-17-20-17-17-16-16	t14090	0.12	ND
ISTOP-152	TAS	Community	1-1-1-1-1-1-1	ST1	1	07-23-21-16-34-33-13	t127	≤0.03	ND
ISTOP-153	TAS	Community	13-13-1-1-12-10-13	ST582	15	07-23-12-34-34-12-12-23-02-12-23	t084	0.12	**DETECTED**
ISTOP-154	TAS	Community	10-40-8-6-10-3-2	ST508	45	08-16-02-16-13-17-34-16-34	t073	≤0.03	ND
ISTOP-155	TAS	Community	3-1-1-8-1-1-1	ST188	188	07-23-12-21-17-34	t189	0.12	ND
ISTOP-156	NSW	Community	3-1-1-8-1-1-1	ST188	188	07-23-12-21-17-34	t189	0.06	ND
ISTOP-157	NSW	Community	13-13-1-1-12-10-13	ST582	15	07-23-12-34-12-12-12-23-02-12-23	t393	0.12	**DETECTED**
ISTOP-158	NSW	Hospital	1-4-1-4-12-1-10	ST5	5	26-23-17-34-17-20-17-12-12-12-16	t1265	≤0.03	**DETECTED**
ISTOP-159	NSW	Hospital	1-4-1-4-12-1-10	ST5	5	26-17-34-17-20-17-12-12-12-16	t7186	0.06	ND
ISTOP-160	NSW	Community	10-14-8-6-10-3-2	ST45	45	08-16-34-16-34	t728	0.06	ND
ISTOP-161	NSW	Hospital	1-4-1-4-12-1-10	ST5	5	26-23-17-34-17-20-17-12-17-16	t002	0.12	ND
ISTOP-162	NSW	Community	1-152-1-8-1-5-11	ST2867	Singleton	07-23-12-21-12-41-20-17-12-12-17	t2016	0.12	ND
ISTOP-163	NSW	Community	10-40-8-6-10-3-2	ST508	45	08-16-02-16-34-13-17-34-16-34	t015	0.06	ND

Isolate	STATE	ONSET	MLST	ST	CC	spa	Spa Type	Vitek® 2 Pen mg/L	blaZ
ISTOP-164	NSW	Community	3-3-1-1-4-4-3	ST8	8	11-19-12-21-17-34-24-34-22-25	t008	0.06	**DETECTED**
ISTOP-165	NSW	Community	3-1-1-8-1-1-1	ST188	188	07-23-12-21-17-34	t189	0.12	ND
ISTOP-166	NSW	Community	1-4-1-4-12-1-10	ST5	5	26-23-17-34-17-20	t2595	0.12	ND
ISTOP-167	NSW	Community	1-4-1-4-12-1-10	ST5	5	26-23-17-34-17-20-17-12-17-16	t002	0.12	ND
ISTOP-168	WA	Community	69-1-14-15-11-19-3	ST1155	101	04-21-12-17-17	UD	0.06	ND
ISTOP-169	WA	Community	10-40-8-6-10-3-2	ST508	45	08-16-02-16-34-13-17-34-16-34	t015	0.06	ND
ISTOP-170	WA	Community	10-14-8-6-10-3-2	ST45	45	08-16-02-16-34-34-13-17-34-16	t8453	≤0.03	ND
ISTOP-171	WA	Community	13-13-1-1-12-10-530	ST3911	15	07-23-12-34-34-12-12-23-02-12-23	t084	0.12	**DETECTED**
ISTOP-172	VIC	Community	3-1-1-1-1-5-3	ST97	97	07-34-33-34	t1028	0.06	ND
ISTOP-173	VIC	Hospital	1-4-1-4-12-1-10	ST5	5	26-23-17-34-17-20-17-12-17-16	t002	0.12	**DETECTED**
ISTOP-174	VIC	Hospital	3-1-14-15-11-19-3	ST1155	101	04-13-21-12-17-20-17-12-17-17	t2078	0.12	**DETECTED**
ISTOP-175	VIC	Community	22-1-14-23-12-4-31	ST88	88	26-12-21-17	UD	0.06	ND
ISTOP-176	VIC	Community	1-4-1-4-12-1-10	ST5	5	26-23-17-34-17-20-17-12-17-16	t002	0.12	ND
ISTOP-178	VIC	Community	1-4-1-4-12-1-10	ST5	5	35-17-34-17-20-17-12-17-16	t442	0.06	ND
ISTOP-179	VIC	Hospital	1-4-1-4-12-1-10	ST5	5	26-23-17-34-17-20-17-12-17-16	t002	0.12	ND
ISTOP-180	VIC	Community	1-4-1-4-12-1-10	ST5	5	26-23-17-12-17-16	t062	0.12	ND
ISTOP-181	VIC	Community	3-3-1-1-4-4-3	ST8	8	11-19-12-21-17-34-24-34-22-25	t008	0.06	ND
ISTOP-182	VIC	Community	22-1-14-23-12-53-31	ST78	88	07-12-21-17-13-34-34-33-34	t786	0.12	ND
ISTOP-184	NSW	Community	1-4-1-4-12-1-10	ST5	5	26-23-17-34-17-20-17-12-12-12-16	t1265	≤0.03	ND
ISTOP-185	NSW	Community	3-1-1-8-1-803-1	ST7259	188	07-23-12-21-17-34	t189	0.06	ND
ISTOP-186	NSW	Community	19-23-15-2-19-20-136	ST1224	59	04-02-17-20-17-31-16-34	t471	≤0.03	ND
ISTOP-187	NSW	Community	1-4-1-4-12-1-10	ST5	5	26-17-34-17-20-17-12-17-16	t010	0.12	**DETECTED**
ISTOP-188	NSW	Community	1-4-1-4-12-1-10	ST5	5	26-23-17-34-17-20-17-12-12-12-16	t1265	0.06	**DETECTED**
ISTOP-189	NSW	Community	10-14-8-6-10-3-2	ST45	45	08-16-02-16-34-13-13-17-13-16-34	t10421	0.06	ND
ISTOP-190	VIC	Community	1-49-60-15-28-38-145	ST5491	Singleton	15-34-16-17-17-23-75	t5925	≤0.03	ND
ISTOP-191	VIC	Community	1-4-1-4-12-1-10	ST5	5	26-23-17-34-17-20-17-12-17-16	t002	0.12	ND
ISTOP-192	VIC	Community	3-1-14-15-11-19-3	ST101	101	04-21-12-17	t643	0.12	ND

Isolate	STATE	ONSET	MLST	ST	CC	spa	Spa Type	Vitek® 2 Pen mg/L	blaZ
ISTOP-193	SA	Community	1-4-1-4-12-1-10	ST5	5	26-23-17-34-17-20-17-12-17-16	t002	0.06	ND
ISTOP-195	SA	Community	851-4-1-4-12-1-10	ST7260	5	26-23-17-34-17-20-17-12-17-16	t002	≤0.03	ND
ISTOP-198	SA	Community	3-1-1-1-1-5-3	ST97	97	26-23-12-21-17-34-34-34-33-34	t1236	0.12	ND
ISTOP-199	VIC	Community	1-3-1-14-11-51-10	ST80	80	26-23-12-34-34-33-34	t042	0.12	ND
ISTOP-200	VIC	Hospital	1-4-1-4-12-1-10	ST5	5	26-23-17-34-17-20-17-12-17-16	t002	0.12	ND
ISTOP-201	VIC	Community	1-4-1-4-12-1-10	ST5	5	35-17-34-17-20-17-12-17-16	t442	0.06	ND
ISTOP-203	SA	Community	852-14-8-6-10-3-2	ST7261	45	164-34-16-34	t2726	≤0.03	ND
ISTOP-204	SA	Hospital	12-4-1-4-12-1-3	ST6	6	11-10-21-17-34-24-34-22-25-25	t701	0.12	ND
ISTOP-205	NSW	Community	3-1-1-8-1-1-1	ST188	188	07-23-12-21-17-34	t189	0.12	ND
ISTOP-206	NSW	Community	1-4-1-4-12-1-10	ST5	5	26-23-17-34-17-20-17-12-17-16	t002	0.06	ND
ISTOP-207	QLD	Community	1-4-1-4-12-1-10	ST5	5	26-273-17-34-17-20-17-12-12-12-16	UD	0.12	**DETECTED**
ISTOP-208	QLD	Community	1-152-1-8-1-5-11	ST2867	Singleton	07-23-12-21-12-41-20-17-12-12-12-17	UD	0.12	ND
ISTOP-209	QLD	Community	1-4-1-4-12-1-10	ST5	5	26-23-17-34-17-20-17-12-17-16	t002	0.06	ND
ISTOP-210	QLD	Community	13-13-1-1-12-10-530	ST3911	15	07-23-12-34-34-12-12-23-02-12-23	t084	0.06	**DETECTED**
ISTOP-211	QLD	Community	3-1-1-1-1-5-3	ST97	97	07-23-12-21-17-02-34	t231	0.06	ND
ISTOP-212	QLD	Community	1-1-1-1-1-1-1	ST1	1	07-23-21-16-34-33-13	t127	0.06	ND
ISTOP-213	VIC	Community	3-35-19-2-20-26-29	ST398	398	08-16-02-25-34-25	t1451	0.12	ND
ISTOP-214	VIC	Community	3-35-19-2-20-26-29	ST398	398	Not Determined	-	0.06	ND
ISTOP-215	VIC	Community	3-3-1-1-4-4-3	ST8	8	11-10-21-21-17-34-24-34-22-25	t10888	0.12	ND
ISTOP-216	TAS	Community	10-14-8-6-10-3-2	ST45	45	09-34	t362	0.06	ND
ISTOP-217	TAS	Community	22-1-14-23-12-53-31	ST78	88	07-12-12-21-17-13-13-34-34-33-34	t2311	0.06	ND
ISTOP-218	TAS	Community	1-4-1-4-12-1-10	ST5	5	26-17-34-17-20-17-12-17-16	t010	0.12	ND
ISTOP-219	TAS	Community	1-1-1-1-1-1-1	ST1	1	07-23-21-16-34-33-13	t127	0.06	ND
ISTOP-220	TAS	Community	5-4-1-4-4-6-3	ST7	7	07-23-21-17-34-12-23-02-20	t7234	0.06	ND
ISTOP-221	TAS	Community	13-13-1-1-12-11-13	ST15	15	07-23-12-34-12-12-23-02-12-23	t346	≤0.03	ND
ISTOP-222	WA	Community	4-3-1-1-11-72-11	ST672	361	26-22-17-20-17-12-17-17-16-16	t1309	0.06	ND
ISTOP-223	WA	Community	13-13-1-1-12-10-13	ST582	15	07-23-12-34-34-12-23-02-12-23	t085	0.06	**DETECTED**

Isolate	STATE	ONSET	MLST	ST	CC	spa	Spa Type	Vitek® 2 Pen mg/L	blaZ
ISTOP-224	WA	Community	1-4-1-4-12-1-10	ST5	5	26-23-17-34-17-20-17-12-17-16	t002	0.12	ND
ISTOP-225	WA	Community	13-13-1-1-12-11-13	ST15	15	07-23-12-34-34-12-12-23-02-12-23	t084	0.12	ND
ISTOP-226	WA	Community	3-35-19-2-20-26-29	ST398	398	08-16-02-25-34-25	t1451	0.06	ND
ISTOP-227	WA	Community	1-4-1-4-12-1-10	ST5	5	26-23-17-34-17-20-17-12-17-16	t002	0.06	ND
ISTOP-228	WA	Community	1-4-1-4-12-577-10	ST5189	5	26-23-17-34-17-20-17-12-12-12-16	t1265	0.06	ND
ISTOP-229	WA	Community	1-4-1-4-12-1-966	ST7262	5	26-23-17-34-17-20-17-12-17-16	t002	0.12	**DETECTED**
ISTOP-230	WA	Community	10-14-8-6-10-3-2	ST45	45	08-16-02-16-34	t230	0.06	ND
ISTOP-231	WA	Community	1-4-1-4-12-1-10	ST5	5	26-23-17-34-17-20-17-12-17-16	t002	0.12	ND
ISTOP-232	WA	Community	1-4-1-4-12-1-10	ST5	5	26-23-17-34-12-17-16	t5081	0.12	ND
ISTOP-233	WA	Hospital	1-4-1-4-12-1-10	ST5	5	26-23-17-34-17-20-17-12-12-16	t179	0.12	**DETECTED**
ISTOP-234	WA	Community	3-1-122-1-1-5-3	ST953	97	07-23-12-21-17-34-34-34-33-34	t267	≤0.03	ND
ISTOP-235	WA	Hospital	13-13-1-1-12-11-13	ST15	15	07-23-12-34-12-12-23-02-12-23	t346	0.06	ND
ISTOP-236	WA	Community	13-13-1-1-12-11-13	ST15	15	07-23-12-34-34-12-23-02-12-23	t085	0.12	ND
ISTOP-237	WA	Community	3-1-122-1-1-5-3	ST953	97	07-23-21-17-34-34-33-34	t2802	0.12	ND
ISTOP-238	WA	Community	1-4-1-4-861-1-10	ST7263	5	26-17-20-17-12-17-16	t045	0.12	ND
ISTOP-239	SA	Community	3-3-1-1-4-4-3	ST8	8	11-10-12-21-17-21-17-34-24-34-22-16	UD	0.06	ND
ISTOP-240	SA	Community	3-35-19-2-20-26-29	ST398	398	08-16-02-25-02-02-25-34-25	t6605	0.06	ND
ISTOP-241	SA	Community	1-4-1-4-12-1-10	ST5	5	26-23-17-34-17-20-17-12-17-16	t002	≤0.03	ND
ISTOP-242	SA	Community	3-1-1-1-1-5-3	ST97	97	07-23-12-21-17-34-34-34-33-34	t267	0.12	ND
ISTOP-243	SA	Community	853-13-1-1-12-11-13	ST7264	15	07-23-12-34-34-12-23-02-12-23	t085	0.06	ND
ISTOP-244	SA	Hospital	12-4-1-4-12-1-3	ST6	6	11-10-21-17-34-24-34-22-25-25	t701	0.06	ND
ISTOP-245	WA	Community	3-1-1-1-1-5-3	ST97	97	07-23-12-21-17-34-34-33-34	t359	0.12	ND
ISTOP-246	WA	Hospital	1-4-1-566-12-1-967	ST7265	5	26-23-17-34-17-20-17-12-17-16	t002	≤0.03	ND
ISTOP-248	WA	Community	12-4-1-4-12-1-3	ST6	6	11-10-21-17-34-24-34-22-25	t304	0.12	ND
ISTOP-249	WA	Community	1-4-1-4-12-1-10	ST5	5	26-23-17-34-17-20-17-12-12-17-16	t088	0.06	ND
ISTOP-250	WA	Community	3-1-1-1-1-5-3	ST97	97	07-23-21-17-34-34-33-34	t3380	0.12	ND
ISTOP-251	WA	Community	1-4-1-4-12-1-968	ST7267	5	26-23-17-34-17-20-17-12-17-16	t002	0.12	ND

Isolate	STATE	ONSET	MLST	ST	CC	spa	Spa Type	Vitek® 2 Pen mg/L	blaZ
ISTOP-252	WA	Community	1-4-1-4-12-1-10	ST5	5	26-17-20-17-12-17-16	t045	0.12	ND
ISTOP-253	WA	Hospital	10-40-8-6-10-3-2	ST508	45	08-16-02-16-34-13-17-34-16-34	t015	0.12	ND
ISTOP-254	WA	Community	1-3-1-8-11-5-11	ST12	12	07-23-21-24-33-22-17	t160	0.06	ND
ISTOP-255	NSW	Community	3-1-122-1-1-5-3	ST953	97	07-23-12-21-17-34-34-33-34	t359	0.06	ND
ISTOP-256	WA	Community	19-23-15-2-41-20-15	ST87	59	04-20-17-31-16-34	t316	0.12	ND
ISTOP-257	WA	Community	12-4-1-4-12-1-3	ST6	6	11-10-21-17-34-24-34-22-25	t304	0.06	ND
ISTOP-259	ACT	Community	3-3-1-1-4-4-3	ST8	8	11-19-12-21-17-34-24-34-22-25	t008	≤0.03	ND
ISTOP-260	ACT	Hospital	10-14-8-6-10-3-2	ST45	45	09-02-16-34-17-34-16-34	t706	0.06	ND
ISTOP-261	ACT	Community	22-1-14-23-12-53-31	ST78	88	07-12-21-17-13-34-13-34-33-34	t2177	0.06	ND
ISTOP-262	ACT	Community	1-4-1-4-12-1-10	ST5	5	26-23-17-34-17-20-17-12-17-16	t002	≤0.03	ND
ISTOP-263	ACT	Hospital	8-2-2-2-6-3-2	ST34	30	04-33-31-12-16-34-16-12-33-34	t089	0.06	**DETECTED**
ISTOP-264	ACT	Hospital	8-2-2-2-6-3-2	ST34	30	04-33-31-12-16-34-16-12-33-34	t089	0.06	**DETECTED**
ISTOP-265	ACT	Community	1-4-1-4-12-1-10	ST5	5	07-23-17-34-17-20-17-12-17-16	t570	0.12	ND
ISTOP-266	ACT	Community	7-6-1-5-8-8-6	ST22	22	26-23-13-23-31-05-17-25-16-28	t474	0.06	ND
ISTOP-267	ACT	Community	1-4-1-4-12-1-10	ST5	5	35-17-12-16	t3660	0.06	ND
ISTOP-268	NSW	Community	854-14-8-6-10-3-2	ST7268	45	08-16-02-16-34-13-17-34-16-34	t015	0.06	ND
ISTOP-269	NSW	Community	10-40-8-6-10-3-2	ST508	45	08-16-34	t026	0.06	ND
ISTOP-270	NSW	Community	1-4-1-4-12-1-10	ST5	5	26-23-17-34-17-20-17-12-12-12-16	t1265	0.06	ND
ISTOP-271	NSW	Hospital	1-4-903-4-12-1-10	ST7269	5	26-23-17-34-17-20-17-12-17-16	t002	0.06	ND
ISTOP-272	TAS	Community	1-4-1-4-12-1-10	ST5	5	26-23-17-34-17-20-17-12-17-16	t002	≤0.03	ND
ISTOP-273	TAS	Community	3-1-122-1-1-5-3	ST953	97	07-23-12-21-17-34-34-34-33-34	t267	0.12	ND
ISTOP-274	TAS	Community	1-3-1-8-11-5-11	ST12	12	07-23-21-24-33-22-17	t160	0.06	ND
ISTOP-275	TAS	Community	1-4-1-4-12-1-10	ST5	5	26-23-17-34-17-20-17-12-17-17-16	t306	≤0.03	ND
ISTOP-276	NSW	Community	13-13-1-1-12-10-530	ST3911	15	07-23-12-34-34-34-12-12-23-02-12-23	t279	0.12	**DETECTED**
ISTOP-277	NSW	Community	1-61-904-8-12-4-20	ST7270	Singleton	11-19-17-20-17-12-17-17-16	UD	0.06	ND
ISTOP-278	NSW	Community	7-6-1-5-8-8-6	ST22	22	07-23-13-23-31-05-17-25-17-25-16-28	t852	0.12	ND
ISTOP-279	NSW	Community	10-14-8-6-10-3-2	ST45	45	08-16-02-16-34-13-17-34-16-34	t015	0.12	ND

Isolate	STATE	ONSET	MLST	ST	CC	spa	Spa Type	Vitek® 2 Pen mg/L	blaZ
ISTOP-280	NSW	Hospital	3-1-1-1-1-5-3	ST97	97	26-23-12-21-17-34-34	t7753	0.12	ND
ISTOP-281	NSW	Community	1-1-1-1-1-1-1	ST1	1	07-23-21-16-34-33-13	t127	0.06	ND
ISTOP-282	NSW	Hospital	1-4-1-4-12-1-10	ST5	5	26-23-17-34-17-20-17-12-12-12-16	t1265	0.12	ND
ISYOP-283	NSW	Community	10-14-8-6-10-3-2	ST45	45	09-34-34-17-16-34	t10771	0.06	ND
ISTOP-284	NSW	Hospital	3-1-14-15-11-19-3	ST101	101	4	t528	0.12	ND
ISTOP-285	NSW	Hospital	12-4-1-4-12-1-3	ST6	6	11-10-21-17-34-22-25	t4407	0.12	ND
ISTOP-286	NSW	Hospital	3-3-1-1-4-4-3	ST8	8	11-19-12-21-17-34-24-34-22-25	t008	0.06	ND
ISTOP-287	NSW	Community	3-1033-1-8-1-1-1	ST7271	188	07-23-12-21-17-34	t189	0.06	ND
ISTOP-288	NSW	Community	1-152-1-8-1-5-11	ST2867	Singleton	07-23-12-21-12-17-20-17-12-12-17	t148	0.06	ND
ISTOP-289	NSW	Hospital	3-1-14-15-11-19-3	ST101	101	4	t528	0.06	ND
ISTOP-290	NSW	Community	1-4-1-4-12-1-10	ST5	5	26-23-17-34-17-20-17-12-17-16	t002	0.06	ND
ISTOP-291	QLD	Community	3-1-1-1-1-5-3	ST97	97	07-23-12-21-17-34-34-34-33-34	t267	0.06	ND
ISTOP-292	QLD	Community	7-1034-1-5-8-8-6	ST7272	22	26-23-13-23-31-05-17-25-17-25-16-28	t005	≤0.03	ND
ISTOP-293	VIC	Hospital	3-3-1-1-1-1-10	ST9	9	07-16-12-23-02-34	t4812	0.12	**DETECTED**
ISTOP-294	VIC	Community	3-1-14-15-11-19-3	ST101	101	4	t528	0.06	ND
ISTOP-295	VIC	Community	13-13-1-1-12-10-530	ST3911	15	07-23-12-34-34-12-12-23-02-12-23	t084	0.12	**DETECTED**
ISTOP-296	VIC	Community	2-2-2-2-2-2-2	ST39	30	15-12-16-16-02-25-17-24	t1504	≤0.03	ND
ISTOP-298	VIC	Community	3-1-1-1-1-5-3	ST97	97	07-23-12-21-17-13-34-33-34	t224	0.12	ND
ISTOP-299	VIC	Community	13-13-1-1-12-11-13	ST15	15	07-23-12-34-34-12-12-23-02-12-23	t084	0.12	ND
ISTOP-300	QLD	Community	13-13-91-1-12-10-13	ST7273	15	07-23-12-34-34-12-12-23-02-12-23	t084	0.12	**DETECTED**
ISTOP-301	QLD	Hospital	1-4-1-4-12-1-10	ST5	5	26-17-34-17-20-17-12-17-16	t010	0.06	ND
ISTOP-302	QLD	Community	4-3-1-1-11-72-11	ST672	361	26-22-17-20-17-12-17-17-16-16	t1309	0.06	ND
ISTOP-303	QLD	Hospital	1-4-1-4-12-1-10	ST5	5	26-23-17-34-17-20-17-12-17-16	t002	0.06	ND
ISTOP-304	QLD	Community	3-35-19-2-20-26-29	ST398	398	08-16-02-25-34-25	t1451	0.12	ND
ISTOP-305	QLD	Community	3-1-14-15-11-19-53	ST7274	101	4	t528	0.06	ND
ISTOP-306	QLD	Community	13-13-1-1-12-10-13	ST582	15	07-23-12-34-34-12-12-23-02-12-23	t084	0.12	**DETECTED**

Isolate	STATE	ONSET	MLST	ST	CC	spa	Spa Type	Vitek® 2 Pen mg/L	blaZ
ISTOP-307	VIC	Community	3-1-1-1-1-5-3	ST97	97	07-23-12-21-17-13-34-33-34	t224	≤0.03	ND
ISTOP-308	VIC	Community	22-1-14-23-12-4-31	ST88	88	26-12-21-17-13-34-34-34-33-34	t3341	0.12	ND
ISTOP-309	VIC	Community	13-13-1-1-12-11-13	ST15	15	07-23-12-34-34-12-12-23-02-12-23	t084	0.12	ND
ISTOP-310	VIC	Community	1-4-1-4-12-1-10	ST5	5	26-23-17-34-17-20-17-12-12-12-16	t1265	0.06	ND
ISTOP-311	VIC	Community	2-2-2-2-6-3-2	ST30	30	15-12-12-16-02-16-02-25-17-24	t3037	0.06	ND
ISTOP-312	NSW	Community	22-1-14-23-12-53-31	ST78	88	07-12-34-33-34	t2191	0.06	ND
ISTOP-313	NSW	Hospital	1-4-1-4-12-1-10	ST5	5	26-23-17-34-17-20-17-12-12-16	t179	0.06	ND
ISTOP-315	VIC	Community	1-4-1-4-12-1-10	ST5	5	26-17-20-17-12-17-16	t045	0.06	ND
ISTOP-316	VIC	Hospital	1-4-1-4-12-1-10	ST5	5	26-23-17-34-17-20-17-12-12-16	t179	0.12	ND
ISTOP-317	VIC	Hospital	3-1-1-8-1-1-1	ST188	188	07-23-12-21-17-34	t189	0.12	ND
ISTOP-318	VIC	Hospital	13-13-1-1-12-10-13	ST582	15	07-23-12-34-34-12-12-23-02-12-23	t084	0.12	**DETECTED**
ISTOP-319	VIC	Community	2-2-2-2-6-3-2	ST30	30	15-12-16-02-16-02-25-17-24-24	t012	0.12	**DETECTED**
ISTOP-320	VIC	Community	3-1-1-1-1-5-3	ST97	97	07-23-21-17-34-34-33-34	t2734	0.12	ND
ISTOP-321	NSW	Community	1-4-1-4-12-1-10	ST5	5	26-23-17-34-17-20-17-12-12-12-16	t1265	0.06	ND
ISTOP-322	NSW	Community	1-4-1-4-12-1-10	ST5	5	26-23-17-34-17-20-17-12-12-12-16	t1265	0.06	ND
ISTOP-323	NSW	Community	3-1035-19-2-20-26-39	ST7275	398	08-16-02-25-02-02-31-25-34-25	UD	0.12	ND
ISTOP-324	VIC	Community	13-13-1-1-12-10-13	ST582	15	07-23-12-34-34-12-12-23-02-12-23	t084	0.06	**DETECTED**
ISTOP-325	VIC	Community	855-1-4-1-5-5-4	ST7276	25	04-21-12-41-20-17-12-12-12-17	t258	≤0.03	**DETECTED**
ISTOP-326	VIC	Community	3-37-19-2-20-26-32	ST291	291	08-16-34-24-34-34-17-17	t937	≤0.03	ND
ISTOP-327	VIC	Community	1-4-1-4-12-1-10	ST5	5	26-17-34-17-20-17-12-17-16	t010	≤0.03	ND
ISTOP-328	NSW	Community	3-1036-19-2-20-26-39	ST7277	398	08-16-02-25-34-25	t1451	0.06	ND
ISTOP-329	NSW	Community	1-4-1-4-12-1-10	ST5	5	26-23-17-34-17-20-17-12-12-12-16	t1265	0.06	ND
ISTOP-330	NSW	Community	1-1-1-1-1-1-1	ST1	1	07-23-21-16-34-33-13	t127	≤0.03	ND
ISTOP-331	NSW	Hospital	3-3-1-1-4-4-3	ST8	8	11-19-12-17-34-24-34-22-25	t1171	0.06	ND
ISTOP-332	NSW	Community	3-1-14-15-11-19-3	ST101	101	4	t528	0.06	ND

Isolate	STATE	ONSET	MLST	ST	CC	spa	Spa Type	Vitek® 2 Pen mg/L	blaZ
ISTOP-333	NSW	Community	22-1-14-23-12-4-31	ST88	88	26-12-21-17-21-17-13-34-34-33-34	t6928	0.12	ND
ISTOP-334	SA	Community	1-4-1-4-12-1-10	ST5	5	26-23-17-34-17-20-17-12-12-12-16	t1265	0.06	ND
ISTOP-335	SA	Community	3-1-1-1-1-5-3	ST97	97	07-23-12-21-17-34-34-34-33-34	t267	≤0.03	ND
ISTOP-336	SA	Community	10-40-8-6-10-3-2	ST508	45	08-16-02-16-34-34-17-34-16-34	t050	0.06	ND
ISTOP-337	WA	Community	10-14-8-6-10-3-2	ST45	45	08-23-16-34-13-293-34-16-34	UD	0.06	ND
ISTOP-338	WA	Community	1-4-463-4-12-1-10	ST3628	5	26-23-17-34-17-20-17-12-12-12-16	t1265	0.06	ND
ISTOP-339	NSW	Community	1-4-1-4-12-1-10	ST5	5	26-23-17-34-17-20-17-12-12-12-16	t1265	0.06	ND
ISTOP-340	NSW	Community	3-1-14-15-11-19-3	ST101	101	4	t528	0.12	ND
ISTOP-342	TAS	Community	4-9-1-8-1-10-8	ST20	20	26-06-17-21-34-34-34-22-34	t2919	0.12	ND
ISTOP-343	TAS	Community	19-23-25-2-19-20-15	ST59	59	07-23-21-17-34-12-23-02-12-23	t471	0.06	ND
ISTOP-344	TAS	Community	3-1037-1-1-1-5-3	ST7278	97	07-23-12-21-17-34-34-33-34	t359	0.12	ND
ISTOP-345	VIC	Community	5-4-1-4-4-6-3	ST7	7	07-23-21-17-34-12-23-02-12-23	t091	0.06	ND
ISTOP-346	VIC	Community	10-40-8-19-10-3-2	ST7279	45	08-16-02-16-34-34-17-34-16-34	t050	0.06	ND
ISTOP-347	WA	Community	7-6-1-5-8-8-6	ST22	22	26-23-13-23-05-17-25-16-28	t3243	0.12	ND
ISTOP-348	WA	Community	13-13-1-1-12-11-13	ST15	15	26-23-12-34-34-12-12-23-02-12-23	t491	0.06	ND
ISTOP-349	WA	Community	1-4-463-4-12-1-10	ST3628	5	26-17-20-17-12-12-12-16	t7026	0.12	ND
ISTOP-350	WA	Community	1-4-1-4-12-1-10	ST5	5	26-23-17-34-17-20-17-12-17-16	t002	0.12	ND
ISTOP-351	WA	Community	1-4-1-4-12-1-10	ST5	5	26-23-17-34-17-20-17-12-12-16	t179	0.12	ND
ISTOP-352	WA	Community	3-1-1-1-1-5-3	ST97	97	7	t693	0.12	ND
ISTOP-353	WA	Community	1-4-1-4-12-1-10	ST5	5	26-23-17-34-17-20-17-12-17-16	t002	≤0.03	ND
ISTOP-354	WA	Community	19-23-15-2-19-20-969	ST7280	59	08-16-02-25-34-25	t216	0.06	ND
ISTOP-355	WA	Community	22-1-14-567-12-53-31	ST7281	88	07-34-34-34-33-34	t730	≤0.03	ND
ISTOP-356	WA	Community	13-13-1-1-12-11-13	ST15	15	07-23	t605	0.06	ND
ISTOP-357	WA	Community	1-4-1-4-12-1-10	ST5	5	26-23-17-34-17-20-17-12-12-12-16-16	t10218	0.06	ND
ISTOP-358	WA	Community	3-1-1-1-1-5-3	ST97	97	07-23-20-12-21-17-34-34-34-33-34	t10212	0.06	ND
ISTOP-359	WA	Hospital	3-1-1-1-1-5-3	ST97	97	23-34-33-34	UD	≤0.03	ND

Isolate	STATE	ONSET	MLST	ST	CC	spa	Spa Type	Vitek® 2 Pen mg/L	blaZ
ISTOP-360	WA	Hospital	7-6-1-5-8-8-6	ST22	22	26-23-13-23-31-29-17-31-12-25-17-25-16-28	UD	0.06	**DETECTED**
ISTOP-361	NSW	Community	3-1-14-15-11-19-3	ST101	101	4	t528	0.12	ND
ISTOP-362	NSW	Hospital	3-35-19-2-20-26-29	ST398	398	08-16-02-25-34-25	t1451	≤0.03	ND
ISTOP-363	NSW	Community	3-35-19-2-20-26-29	ST398	398	08-16-02-25-34-25	t1451	0.06	ND
ISTOP-364	NSW	Community	1-1-1-1-1-1-1	ST1	1	07-23-21-16-34-33-13	t127	0.12	ND
ISTOP-365	NSW	Community	1-4-1-4-12-1-10	ST5	5	26-17-34-17-20-17-17-16	t17484	0.12	ND
ISTOP-366	NSW	Community	10-14-8-6-10-3-2	ST45	45	09-34	t362	0.06	ND
ISTOP-367	NSW	Community	3-1-1-1-1-5-3	ST97	97	07-23-12-21-17-34-34-34-33-34	t267	0.12	ND
ISTOP-368	NSW	Community	19-23-15-2-19-20-15	ST59	59	04-20-17-31-16-34	t316	0.12	ND
ISTOP-369	NSW	Community	1-4-1-4-12-1-10	ST5	5	26-23-17-34-17-20-17-12-12-17-16	t088	0.12	ND
ISTOP-370	NSW	Community	1-1038-1-4-12-1-10	ST7282	5	26-23-17-34-17-20-17-12-12-12-16	t1265	0.06	ND
ISTOP-372	QLD	Community	1-4-1-4-12-1-10	ST5	5	26-17-34-17-20-17-12-12-12-16	t7186	0.06	ND
ISTOP-373	VIC	Community	10-14-8-6-10-3-2	ST45	45	08-16-02-16-34-13-17-34-16-34	t015	0.06	ND
ISTOP-374	VIC	Community	1-4-1-4-12-1-10	ST5	5	26-23-17-34-17-20-17-12-17-16	t002	0.06	ND
ISTOP-375	VIC	Community	12-4-1-4-12-1-3	ST6	6	11-10-21-17-34-24-34-22-25	t304	0.06	ND
ISTOP-376	WA	Hospital	10-14-8-6-10-3-2	ST45	45	08-16-02-16-34-13-17-34-16-34	t015	0.06	ND
ISTOP-377	NSW	Community	1-3-1-8-11-5-11	ST12	12	07-23-21-24-33-22-22-17	t771	0.06	ND
ISTOP-378	ACT	Community	1-4-1-4-12-1-10	ST5	5	26-23-17-34-17-20-17-12-17-16-17-16	t494	0.12	ND
ISTOP-379	ACT	Community	13-13-1-1-12-11-13	ST15	15	07-23-12-34-34-23	t5393	0.06	ND
ISTOP-380	ACT	Community	1-4-1-4-12-1-10	ST5	5	26-23-17-34-17-20-17-12-12-16	t179	0.06	ND
ISTOP-381	ACT	Community	1-4-1-4-12-1-10	ST5	5	26-23-17-34-17-20-17-12-17-16	t002	0.06	ND
ISTOP-382	VIC	Community	1-4-1-4-12-1-10	ST5	5	26-23-17-34-17-20-17-12-17-16	t002	≤0.03	ND
ISYOP-383	VIC	Hospital	7-6-1-5-8-8-6	ST22	22	26-23-13-23-05-17-25-17-25-16-16-28	t2933	0.12	ND
ISTOP-384	VIC	Hospital	7-6-1-5-8-8-6	ST22	22	26-17-25-16-28	t1328	≤0.03	ND
ISTOP-385	QLD	Community	3-1-1-1-1-5-3	ST97	97	07-23-12-21-17-34-34-34-33-34	t267	0.06	ND
ISTOP-386	QLD	Community	10-14-8-6-10-3-2	ST45	45	08-16-02-16-34-13-17-13-16-34	t302	0.06	ND
ISTOP-388	VIC	Community	1-4-1-4-12-1-10	ST5	5	26-17-20-17-12-17-16	t045	0.06	ND

Isolate	STATE	ONSET	MLST	ST	CC	spa	Spa Type	Vitek® 2 Pen mg/L	blaZ
ISTOP-389	VIC	Community	1-4-1-4-12-1-10	ST5	5	26-23-17-34-17-20-17-12-17-16	t002	0.06	ND
ISTOP-390	VIC	Community	1-4-1-4-12-1-10	ST5	5	26-23-17-34-17-20-17-17-16	t105	0.06	ND
ISTOP-391	VIC	Community	1-4-1-4-12-1-10	ST5	5	26-23-17-12-17-16	t062	0.06	ND
ISTOP-392	NSW	Community	3-1-122-1-1-5-3	ST953	97	07-23-12-21-17-34-34-34-33-34	t267	≤0.03	ND
ISTOP-393	NSW	Community	3-1-14-15-11-19-3	ST101	101	4	t528	≤0.03	ND
ISTOP-394	WA	Community	1-4-1-4-12-1-10	ST5	5	26-23-17-13-17-20-17-12-17-16	t242	≤0.03	ND
ISTOP-395	WA	Hospital	1-4-1-4-12-1-10	ST5	5	26-23-17-34-17-20-17-12-17-16	t002	0.06	ND
ISTOP-396	WA	Community	1-1-1-1-1-1-1	ST1	1	07-23-21-16-34-33-13	t127	0.12	ND
ISTOP-397	WA	Community	3-3-1-1-4-4-3	ST8	8	11-19-12-21-17-34-24-34-22-25	t008	0.06	ND
ISTOP-398	WA	Hospital	1-4-1-4-12-1-10	ST5	5	26-23-17-34-17-20-17-12-12-12-16	t1265	0.06	ND
ISTOP-399	WA	Community	1-4-1-4-12-1-10	ST5	5	26-23-17-34-17-20-17-12-12-12-16	t1265	0.12	ND
ISTOP-400	WA	Community	1-4-1-4-12-1-10	ST5	5	26-23-17-34-16-16	t6071	0.12	ND
ISTOP-401	VIC	Community	13-13-1-1-12-11-970	ST7283	15	07-23-12-23-02-12-23	t547	0.12	**DETECTED**
ISTOP-402	VIC	Community	12-4-1-4-12-1-3	ST6	6	11-10-21-17-34-24-34-22-25-25	t701	0.06	ND
ISTOP-403	VIC	Community	19-23-15-2-19-20-15	ST59	59	07-06-17-21-34-34-22-34	t437	0.12	ND
ISTOP-404	VIC	Community	144-1-1-1-1-5-3	ST1179	97	07-23-12-21-17-34-34-34-33-34	t267	0.12	ND
ISTOP-405	VIC	Community	3-3-1-1-4-4-3	ST8	8	11-19-12-21-17-34-24-34-22-25	t008	0.12	ND
ISTOP-406	VIC	Community	1-3-1-8-11-5-11	ST12	12	07-23-21-24-33-22-17	t160	0.06	ND
ISTOP-407	VIC	Hospital	1-1-1-1-1-1-1	ST1	1	14-21-16-34-33-13	t174	0.12	ND
ISTOP-408	VIC	Community	10-40-8-6-10-3-2	ST508	45	08-16-02-16-34-13-17-34-16-34	t015	0.06	ND
ISTOP-409	NSW	Community	1-1-1-1-1-1-1	ST1	1	07-23-21-16-34-33-13	t127	0.06	ND
ISTOP-410	NSW	Community	1-4-1-4-12-1-10	ST5	5	26-23-17-34-17-20-17-12-12-12-16	t1265	0.06	ND
ISTOP-411	NSW	Community	3-1-14-15-11-19-3	ST101	101	04-13-21-12-17-20-17-12-17-17	t2078	0.06	ND
ISTOP-412	NSW	Community	1-4-1-4-12-1-10	ST5	5	26-23-17-34-17-20	t2595	0.06	ND
ISTOP-413	NSW	Community	1-4-1-4-12-1-10	ST5	5	26-23-17-34-17-20-17-12-12-12-16	t1265	0.06	ND
ISTOP-414	NSW	Community	3-1-1-1-1-5-3	ST97	97	07-23-12-21-17-34-34-33-34	t359	0.06	ND
ISTOP-415	NSW	Community	1-4-1-4-12-1-10	ST5	5	26-23-17-34-17-20-17-12-12-16	t179	0.06	ND

Isolate	STATE	ONSET	MLST	ST	CC	spa	Spa Type	Vitek® 2 Pen mg/L	blaZ
ISTOP-416	NSW	Community	3-1-1-1-1-5-3	ST97	97	07-34-33-34	t1028	0.12	ND
ISTOP-417	NSW	Community	3-1-1-8-1-1-1	ST188	188	07-23-12-21-17-34	t189	≤0.03	ND
ISTOP-418	NSW	Community	3-37-19-2-20-26-32	ST291	291	08-16-34-34-34-17-17	t16932	0.06	ND
ISTOP-419	NSW	Community	10-1039-8-6-10-3-2	ST7284	45	09-02-16-34-13-17-34	t715	0.12	ND
ISTOP-420	NSW	Community	3-1-1-1-1-5-3	ST97	97	07-23-12-21-17-34-34-34-33-34	t267	0.12	ND
ISTOP-421	NSW	Community	1-4-1-4-12-1-10	ST5	5	26-23-17-34-17-20-17-12-17-16	t002	0.06	ND
ISTOP-422	NSW	Community	3-1-1-1-1-5-3	ST97	97	07-23-12-21-17-34-34-34-33-34	t267	0.12	ND
ISTOP-423	NSW	Community	1-4-1-4-12-1-10	ST5	5	26-23-17-34-12-16	t5349	0.12	ND
ISTOP-424	NSW	Community	1-4-1-4-12-1-10	ST5	5	26-23-17-34-17-20-17-12-17-16	t002	0.06	ND
ISTOP-425	NSW	Community	1-4-1-4-12-1-10	ST5	5	26-23-17-34-17-20-17-12-12-12-16	t1265	0.12	ND
ISTOP-426	SA	Hospital	10-14-8-6-10-3-2	ST45	45	09-02-16-34-16-34	t371	0.06	ND
ISTOP-427	SA	Hospital	4-9-1-8-1-10-8	ST20	20	07-06-17-21-34-34-22-34	t164	0.12	**DETECTED**
ISTOP-428	VIC	Community	12-4-1-4-12-1-3	ST6	6	11-10-21-17-34-24-34-22-25-25	t701	≤0.03	ND
ISTOP-429	VIC	Community	12-4-1-4-12-1-3	ST6	6	11-10-12-34-24-34-22-25	t4298	0.06	ND
ISTOP-430	TAS	Community	1-4-1-4-12-1-10	ST5	5	26-23-17-34-17-20-17-12-12-12-16	t1265	0.06	ND
ISTOP-431	TAS	Community	1-4-1-4-12-1-10	ST5	5	26-23-17-34-17-20-17-12-16	t548	≤0.03	ND
ISTOP-432	TAS	Community	1-4-1-4-12-1-10	ST5	5	26-23-17-34-17-20-17-12-12-12	t6181	0.06	ND
ISTOP-433	TAS	Community	1-1-1-1-1-1-1	ST1	1	07-23-23-16-34-33-13	t1909	0.06	ND
ISTOP-434	TAS	Community	3-1-1-1-1-5-3	ST97	97	07-23-12-21-17-02-34	t231	0.12	ND
ISTOP-435	TAS	Community	7-6-83-5-8-8-6	ST7285	22	26-17-25-16-28	UD	0.06	ND
ISTOP-436	NSW	Community	1-4-1-4-12-1-10	ST5	5	26-23-17-34-17-20-17-12-17-16	t002	0.12	ND
ISTOP-437	NSW	Community	19-23-15-2-19-20-15	ST59	59	04-20-17-02-17-31-16-34	t1293	0.06	ND
ISTOP-438	NSW	Community	13-13-1-1-12-11-13	ST15	15	07-23-13-34-34-12-12-23-02-12-23	t11928	0.12	**DETECTED**
ISTOP-439	NT	Community	10-14-8-6-10-3-2	ST45	45	09-02-16-34-16-34	t371	0.12	ND
ISTOP-440	NT	Community	3-1-1-8-1-1-1	ST188	188	07-23-12-21-17-34	t189	0.12	ND
ISTOP-441	NT	Community	22-1-14-23-12-4-31	ST88	88	26-12-21-17-13-34-34-33-34	t4013	0.12	ND
ISTOP-442	NT	Community	1-1-1-1-1-1-1	ST1	1	07-23-21-16-34-33-13	t127	≤0.03	ND
ISTOP-443	NT	Community	3-1-122-1-1-5-3	ST953	97	07-23-12-21-17-34-34-34-33-34	t267	≤0.03	ND

Isolate	STATE	ONSET	MLST	ST	CC	spa	Spa Type	Vitek® 2 Pen mg/L	blaZ
ISTOP-444	NT	Community	22-1-14-23-12-4-31	ST88	88	26-12-21-17-21-17-34-34-34-33-34	UD	≤0.03	ND
ISTOP-445	NSW	Community	13-13-1-1-12-11-971	ST7286	15	07-23-12-34-34-12-12-23-02-02-12-23	t144	≤0.03	ND
ISTOP-446	NSW	Hospital	3-1-1-8-1-1-1	ST188	188	07-20-12-21-17-34	UD	0.06	ND
ISTOP-447	NSW	Community	13-13-1-1-12-10-13	ST582	15	07-23-12-23	t1509	0.06	**DETECTED**
ISTOP-448	NSW	Community	3-35-19-2-20-26-29	ST398	398	08-16-02-25-34-25	t1451	≤0.03	ND
ISTOP-449	NSW	Community	3-1-1-1-1-5-3	ST97	97	07-23-12-21-17-34-34-34-33-34	t267	0.12	ND
ISTOP-450	NSW	Community	1-4-1-4-12-1-10	ST5	5	26-23-17-34-17-20-17-12-17-16	t002	≤0.03	ND
ISTOP-451	NSW	Community	1-4-1-4-12-1-10	ST5	5	26-23-17-34-17-20-17-12-17-16	t002	0.12	ND
ISTOP-452	NSW	Hospital	1-4-1-4-12-1-10	ST5	5	Not Determined	-	0.06	**DETECTED**
ISTOP-453	NSW	Community	3-37-19-2-862-26-32	ST7287	291	08-16-34-24-34-34-17-17	t937	0.12	ND
ISTOP-454	NSW	Community	10-14-8-6-10-3-2	ST45	45	08-16-02-16-34-13-17-34-16-13	t1510	0.06	ND
ISTOP-455	NSW	Hospital	10-14-8-6-10-3-2	ST45	45	08-16-02-16-34-13-17-34-16-13	t1510	0.06	ND
ISTOP-456	NSW	Community	10-14-8-6-10-3-2	ST45	45	08-16-34	t026	≤0.03	ND
ISTOP-457	NSW	Community	1-1040-1-4-12-1-10	ST7288	5	26-23-17-34-17-16	t688	0.06	ND
ISTOP-458	QLD	Community	3-1-1-8-1-1-1	ST188	188	07-23-12-21-17-34	t189	0.12	**DETECTED**
ISTOP-459	QLD	Community	1-4-1-4-12-1-10	ST5	5	26-23-17-17-20-17-12-17-16	t579	0.12	**DETECTED**
ISTOP-460	QLD	Community	3-1-1-8-1-1-1	ST188	188	07-23-12-21-17-34	t189	0.06	ND
ISTOP-461	QLD	Community	1-4-1-4-12-1-10	ST5	5	26-23-17-12-12-16	t1531	0.06	ND
ISTOP-462	QLD	Community	13-13-1-1-12-10-13	ST582	15	07-23-12-34-12-12-12-23-02-12-23	t393	0.12	**DETECTED**
ISTOP-463	QLD	Community	10-14-901-6-10-3-2	ST7289	45	08-16-34	t026	0.12	ND
ISTOP-464	QLD	Community	3-1-1-8-1-1-1	ST188	188	07-23-12-34	t416	0.06	ND
ISTOP-465	QLD	Community	13-13-1-1-12-10-13	ST582	15	07-23-12-34-12-12-12-23-02-12-23	t393	0.12	**DETECTED**
ISTOP-466	QLD	Community	1-4-1-4-863-1-10	ST7290	5	26-23-17-34-17-20-17-12-17-16	t002	0.12	ND
ISTOP-467	QLD	Community	3-1-1-8-1-1-1	ST188	188	07-23-12-02-17-34	UD	0.06	ND
ISTOP-468	QLD	Community	10-14-8-6-10-3-2	ST45	45	08-16-02-16-34-34-13-17-34-16-34	t589	≤0.03	ND
ISTOP-469	QLD	Community	3-1-1-1-1-5-3	ST97	97	07-23-12-21-17-34-34-34-33-34	t267	0.06	ND
ISTOP-470	QLD	Community	10-14-8-6-10-3-2	ST45	45	08-16-02-16-34-13-17-34-34	t1078	0.06	ND

Isolate	STATE	ONSET	MLST	ST	CC	spa	Spa Type	Vitek® 2 Pen mg/L	blaZ
ISTOP-471	QLD	Community	22-1-14-23-12-53-31	ST78	88	07-12-21-17-13-13-34-34-33-34	t186	0.06	ND
ISTOP-472	QLD	Hospital	3-1-1-8-1-1-1	ST188	188	07-23-12-21-17-34	t189	0.06	ND
ISTOP-473	QLD	Hospital	10-14-8-6-10-3-2	ST45	45	08-16-34	t026	≤0.03	ND
ISTOP-474	QLD	Community	3-1-1-1-1-5-3	ST97	97	07-23-12-21-17-34-34-34-33-34	t267	0.06	ND
ISTOP-475	QLD	Hospital	13-13-1-1-12-10-13	ST582	15	07-23-12-34-34-12-12-23-02-12-23	t084	0.12	**DETECTED**
ISTOP-476	QLD	Community	1-4-1-4-12-1-10	ST5	5	26-23-17-34-17-02-17-12-12-12-16	t5150	≤0.03	ND
ISTOP-477	QLD	Community	3-1-1-1-1-5-3	ST97	97	07-16-21-17-34-34-34	UD	0.06	ND
ISTOP-478	QLD	Community	1-4-1-4-12-1-10	ST5	5	26-17-20-17-12-17-16	t045	0.06	ND
ISTOP-479	QLD	Community	12-4-1-4-12-1-3	ST6	6	11-10-21-17-34-24-34-22-25-25	t701	0.06	ND
ISTOP-480	QLD	Community	1-4-1-4-12-1-10	ST5	5	26-23-17-34-17-20-17-12-12-12-16	t1265	0.06	ND
ISTOP-481	QLD	Hospital	1-4-1-4-12-1-10	ST5	5	26-23-17-34-17-12-12-17-16	t2666	0.12	ND
ISTOP-482	QLD	Community	1-4-1-4-12-1-968	ST7267	5	07-23-17-34-17-20-17-12-17-16	t570	0.06	ND
ISYOP-483	QLD	Community	1-1-1-1-12-1-1	ST573	Singleton	26-23-13-21-17-34-34-34-33-34	t1839	0.12	ND
ISTOP-484	QLD	Hospital	3-1-14-15-11-19-3	ST101	101	04-20-12-17-20-17-12-17-17	t056	0.12	ND
