# Supplementary material for: Molecular Epidemiology of Penicillin-Susceptible Staphylococcus aureus Bacteremia in Australia and Reliability of Diagnostic Phenotypic Susceptibility Methods to Detect Penicillin Susceptibility
Source: Microorganisms. 2022 Aug 15;10(8):1650. doi: 10.3390/microorganisms10081650 (PMC9413241; doi:10.3390/microorganisms10081650)
Supplement: Supplementary file 1 [file microorganisms-10-01650-s001.zip › Supplementary Table 2.pdf]

Table S2: Multilocus sequence type, origin, Vitek® 2 penicillin minimum inhibitory concentration, detection of *blaZ*, antibiogram and resistance gene profile on 470 penicillin-susceptible *Staphylococcus aureus* identified in the Australian Group for Antimicrobial Resistance's 2020 Australian *Staphylococcus aureus* Sepsis Outcome Program

Isolate	ST	STATE	Vitek 2 Pen MIC mg/L	*blaZ*	Antibiogram	Resistance Genes
**Clonal Complex 1 (n=17)**						
ISTOP-48	ST1	NSW	0.12	-	FA^R	*fusC*
ISTOP-127	ST1	SA	0.12	-	FA^R	fusC
ISTOP-141	ST1	VIC	≤0.03	-	FA^R	*fusC*
ISTOP-152	ST1	TAS	≤0.03	-		
ISTOP-212	ST1	QLD	0.06	-		*fusC*
ISTOP-219	ST1	TAS	0.06	-		*fusC*
ISTOP-281	ST1	NSW	0.06	-	FA^R	*fusC*
ISTOP-330	ST1	NSW	≤0.03	-		
ISTOP-364	ST1	NSW	0.12	-	Cip^R, FA^R	*fusC,* GrlA S80F and GyrA S84 mutations
ISTOP-396	ST1	WA	0.12	-	Cip^R, FA^R	*fusC,* GrlA S80F and GyrA S84 mutations
ISTOP-407	ST1	VIC	0.12	-		
ISTOP-409	ST1	NSW	0.06	-	FA^R	*fusC*
ISTOP-433	ST1	TAS	0.06	-	FA^R	*fusC*
ISTOP-442	ST1	NT	≤0.03	-	FA^R	*fusC*
ISTOP-130	ST3949	NSW	0.12	-	FA^R	*fusC*
ISTOP-21	ST4100	VIC	≤0.03	-		
ISTOP-69	ST4100	NSW	0.06	-		
**Clonal Complex 5 (n=150)**						
ISTOP-3	ST5	WA	0.06	-		
ISTOP-9	ST5	WA	≤0.03	-		
ISTOP-15	ST5	VIC	0.06	-		
ISTOP-18	ST5	VIC	0.06	-		
ISTOP-19	ST5	VIC	0.12	-		
ISTOP-22	ST5	VIC	≤0.03	-		
ISTOP-28	ST5	VIC	0.06	-		
ISTOP-32	ST5	QLD	0.12	-		
ISTOP-33	ST5	NSW	0.06	-		
ISTOP-36	ST5	NSW	0.06	-		
ISTOP-38	ST5	TAS	0.12	-		
ISTOP-49	ST5	NSW	≤0.03	-		
ISTOP-60	ST5	VIC	0.06	-		
ISTOP-63	ST5	VIC	0.06	-		
ISTOP-64	ST5	NSW	0.12	-		
ISTOP-66	ST5	NSW	0.06	-		
ISTOP-81	ST5	NSW	0.12	-		
ISTOP-82	ST5	TAS	≤0.03	-		
ISTOP-83	ST5	TAS	0.06	-		
ISTOP-89	ST5	TAS	0.06	-		
ISTOP-90	ST5	SA	0.12	-		
ISTOP-93	ST5	SA	0.12	-		
ISTOP-95	ST5	SA	0.06	-		

Isolate	ST	STATE	Vitek 2 Pen MIC mg/L	*blaZ*	Antibiogram	Resistance Genes
ISTOP-102	ST5	VIC	≤0.03	-		
ISTOP-103	ST5	SA	0.06	-		
ISTOP-106	ST5	NSW	0.06	-	SXT^R	*dfrG*
ISTOP-107	ST5	NSW	0.12	-		
ISTOP-110	ST5	QLD	≤0.03	-		
ISTOP-111	ST5	QLD	0.06	-	Cli^R, Ery^R	*erm(C)*
ISTOP-113	ST5	NSW	≤0.03	-		
ISTOP-116	ST5	SA	0.12	-		
ISTOP-118	ST5	WA	0.12	-		
ISTOP-124	ST5	SA	0.12	-		
ISTOP-128	ST5	SA	0.12	-		
ISTOP-142	ST5	SA	0.06	-		*fexA*
ISTOP-145	ST5	ACT	0.06	-		
ISTOP-148	ST5	ACT	0.06	-		
ISTOP-158	ST5	NSW	≤0.03	**DETECTED**		
ISTOP-159	ST5	NSW	0.06	-		
ISTOP-161	ST5	NSW	0.12	-		
ISTOP-166	ST5	NSW	0.12	-		
ISTOP-167	ST5	NSW	0.12	-		
ISTOP-173	ST5	VIC	0.12	**DETECTED**		
ISTOP-176	ST5	VIC	0.12	-		
ISTOP-178	ST5	VIC	0.06	-	Cip^R, SXT^R	*dfrG,* GrlA S80F and GyrA S84 mutations
ISTOP-179	ST5	VIC	0.12	-		
ISTOP-180	ST5	VIC	0.12	-		
ISTOP-184	ST5	NSW	≤0.03	-		
ISTOP-187	ST5	NSW	0.12	**DETECTED**		
ISTOP-188	ST5	NSW	0.06	**DETECTED**		
ISTOP-191	ST5	VIC	0.12	-		
ISTOP-193	ST5	SA	0.06	-		
ISTOP-200	ST5	VIC	0.12	-		
ISTOP-201	ST5	VIC	0.06	-	Cip^R, SXT^R	*dfrG,* GrlA S80F and GyrA S84 mutations
ISTOP-206	ST5	NSW	0.06	-		
ISTOP-207	ST5	QLD	0.12	**DETECTED**	Cli^R, Ery^R	*erm(C)*
ISTOP-209	ST5	QLD	0.06	-	Ery^R	
ISTOP-218	ST5	TAS	0.12	-		
ISTOP-224	ST5	WA	0.12	-		
ISTOP-227	ST5	WA	0.06	-		
ISTOP-231	ST5	WA	0.12	-		
ISTOP-232	ST5	WA	0.12	-		
ISTOP-233	ST5	WA	0.12	**DETECTED**		*ant(9)-Ia_1, erm(A), erm(C), tet(K)*
ISTOP-241	ST5	SA	≤0.03	-		
ISTOP-249	ST5	WA	0.06	-		
ISTOP-252	ST5	WA	0.12	-		
ISTOP-262	ST5	ACT	≤0.03	-		
ISTOP-265	ST5	ACT	0.12	-		

Isolate	ST	STATE	Vitek 2 Pen MIC mg/L	*blaZ*	Antibiogram	Resistance Genes
ISTOP-267	ST5	ACT	0.06	-		
ISTOP-270	ST5	NSW	0.06	-		
ISTOP-272	ST5	TAS	≤0.03	-		
ISTOP-275	ST5	TAS	≤0.03	-		
ISTOP-282	ST5	NSW	0.12	-		
ISTOP-290	ST5	NSW	0.06	-		
ISTOP-301	ST5	QLD	0.06	-		*erm(B), tet(M)*
ISTOP-303	ST5	QLD	0.06	-		
ISTOP-310	ST5	VIC	0.06	-		
ISTOP-313	ST5	NSW	0.06	-		
ISTOP-315	ST5	VIC	0.06	-		
ISTOP-316	ST5	VIC	0.12	-		
ISTOP-321	ST5	NSW	0.06	-		
ISTOP-322	ST5	NSW	0.06	-		
ISTOP-327	ST5	VIC	≤0.03	-		
ISTOP-329	ST5	NSW	0.06	-		
ISTOP-334	ST5	SA	0.06	-		
ISTOP-339	ST5	NSW	0.06	-		
ISTOP-350	ST5	WA	0.12	-		
ISTOP-351	ST5	WA	0.12	-		
ISTOP-353	ST5	WA	≤0.03	-		
ISTOP-357	ST5	WA	0.06	-		
ISTOP-365	ST5	NSW	0.12	-		
ISTOP-369	ST5	NSW	0.12	-		
ISTOP-372	ST5	QLD	0.06	-		
ISTOP-374	ST5	VIC	0.06	-		
ISTOP-378	ST5	ACT	0.12	-		
ISTOP-380	ST5	ACT	0.06	-		
ISTOP-381	ST5	ACT	0.06	-		*mdf(A)_1*
ISTOP-382	ST5	VIC	≤0.03	-		
ISTOP-388	ST5	VIC	0.06	-		
ISTOP-389	ST5	VIC	0.06	-		
ISTOP-390	ST5	VIC	0.06	-		
ISTOP-391	ST5	VIC	0.06	-		
ISTOP-394	ST5	WA	≤0.03	-		
ISTOP-395	ST5	WA	0.06	-		
ISTOP-398	ST5	WA	0.06	-		
ISTOP-399	ST5	WA	0.12	-		
ISTOP-400	ST5	WA	0.12	-	Tet^R	*fexA, tet(M)*
ISTOP-410	ST5	NSW	0.06	-		
ISTOP-412	ST5	NSW	0.06	-	Cli^R, Ery^R, Tet^R	*erm(C), tet(M)*
ISTOP-413	ST5	NSW	0.06	-		*tet(M)*
ISTOP-415	ST5	NSW	0.06	-		
ISTOP-421	ST5	NSW	0.06	-		
ISTOP-423	ST5	NSW	0.12	-		
ISTOP-424	ST5	NSW	0.06	-		
ISTOP-425	ST5	NSW	0.12	-		
ISTOP-430	ST5	TAS	0.06	-		

**Isolate**	**ST**	**STATE**	**Vitek 2 Pen MIC mg/L**	***blaZ***	**Antibiogram**	**Resistance Genes**
ISTOP-431	ST5	TAS	≤0.03	-		
ISTOP-432	ST5	TAS	0.06	-		
ISTOP-436	ST5	NSW	0.12	-		
ISTOP-450	ST5	NSW	≤0.03	-		
ISTOP-451	ST5	NSW	0.12	-		
ISTOP-452	ST5	NSW	0.06	**DETECTED**		
ISTOP-459	ST5	QLD	0.12	**DETECTED**		
ISTOP-461	ST5	QLD	0.06	-	Cip^R, Cli^R, Ery^R, SXT^R	*dfrG, erm(C),* GrlA S80F and GyrA S84 mutations
ISTOP-476	ST5	QLD	≤0.03	-	Cli^R, Ery^R, Tet^R	*erm(C), tet(M)*
ISTOP-478	ST5	QLD	0.06	-		
ISTOP-480	ST5	QLD	0.06	-		
ISTOP-481	ST5	QLD	0.12	-		*erm(C)*
ISTOP-4	ST2967	WA	≤0.03	-		
ISTOP-13	ST3628	WA	0.06	-	Ery^R	*erm(C)*
ISTOP-34	ST3628	NSW	≤0.03	-		
ISTOP-39	ST3628	TAS	0.12	-		
ISTOP-121	ST3628	WA	0.12	**DETECTED**		
ISTOP-338	ST3628	WA	0.06	-		
ISTOP-349	ST3628	WA	0.12	-		
ISTOP-104	ST3724	SA	0.06	-	Tet^R	*tet(M)*
ISTOP-62	ST5189	VIC	0.06	-		
ISTOP-228	ST5189	WA	0.06	-		
ISTOP-41	ST7252	VIC	0.12	-		
ISTOP-61	ST7252	VIC	0.06	-		
ISTOP-195	ST7260	SA	≤0.03	-		
ISTOP-229	ST7262	WA	0.12	**DETECTED**		
ISTOP-238	ST7263	WA	0.12	-		
ISTOP-246	ST7265	WA	≤0.03	-	Rif^R	RpoB S464P mutaion
ISTOP-251	ST7267	WA	0.12	-		
ISTOP-482	ST7267	QLD	0.06	-		
ISTOP-271	ST7269	NSW	0.06	-		
ISTOP-370	ST7282	NSW	0.06	-		
ISTOP-457	ST7288	NSW	0.06	-		
ISTOP-466	ST7290	QLD	0.12	-		
**Clonal Complex 6 (n-16)**						
ISTOP-1	ST6	NSW	0.06	-		
ISTOP-53	ST6	NSW	0.12	**DETECTED**		
ISTOP-87	ST6	TAS	≤0.03	-		
ISTOP-91	ST6	SA	0.12	-		
ISTOP-105	ST6	NSW	0.12	-	Rif^R	RpoB S464P mutaion
ISTOP-114	ST6	NSW	0.12	-		
ISTOP-204	ST6	SA	0.12	-		
ISTOP-244	ST6	SA	0.06	-		
ISTOP-248	ST6	WA	0.12	-		
ISTOP-257	ST6	WA	0.06	-		
ISTOP-285	ST6	NSW	0.12	-		

Isolate	ST	STATE	Vitek 2 Pen MIC mg/L	*blaZ*	Antibiogram	Resistance Genes
ISTOP-375	ST6	VIC	0.06	-		
ISTOP-402	ST6	VIC	0.06	-		
ISTOP-428	ST6	VIC	≤0.03	-		
ISTOP-429	ST6	VIC	0.06	-		
ISTOP-479	ST6	QLD	0.06	-		
**Clonal Complex 7 (n=3)**						
ISTOP-125	ST7	SA	0.12	-		
ISTOP-220	ST7	TAS	0.06	-	FA^R	
ISTOP-345	ST7	VIC	0.06	-		
**Clonal Complex 8 (n=14)**						
ISTOP-17	ST8	VIC	0.12	-		
ISTOP-78	ST8	ACT	0.06	-		
ISTOP-143	ST8	SA	≤0.03	-		
ISTOP-144	ST8	ACT	0.06	-		
ISTOP-164	ST8	NSW	0.06	**DETECTED**		
ISTOP-181	ST8	VIC	0.06	-		
ISTOP-215	ST8	VIC	0.12	-		
ISTOP-239	ST8	SA	0.06	-		
ISTOP-259	ST8	ACT	≤0.03	-		
ISTOP-286	ST8	NSW	0.06	-	Ery^R	*erm(C)*
ISTOP-331	ST8	NSW	0.06	-		
ISTOP-397	ST8	WA	0.06	-		
ISTOP-405	ST8	VIC	0.12	-		
ISTOP-6	ST5234	WA	0.06	-		
**Clonal Complex 9 (n=1)**						
ISTOP-293	ST9	VIC	0.12	**DETECTED**		
**Clonal Complex 12 (n=16)**						
ISTOP-12	ST12	WA	≤0.03	-		
ISTOP-14	ST12	WA	0.06	-		
ISTOP-29	ST12	QLD	≤0.03	-		
ISTOP-30	ST12	QLD	0.06	-		
ISTOP-40	ST12	TAS	0.12	-		
ISTOP-51	ST12	NSW	0.06	-		
ISTOP-74	ST12	NSW	0.06	-		
ISTOP-86	ST12	TAS	0.06	-		
ISTOP-117	ST12	WA	0.06	-		
ISTOP-140	ST12	VIC	0.12	-		
ISTOP-150	ST12	ACT	0.06	-		
ISTOP-254	ST12	WA	0.06	-		
ISTOP-274	ST12	TAS	0.06	-		
ISTOP-377	ST12	NSW	0.06	-		
ISTOP-406	ST12	VIC	0.06	-		
ISTOP-23	ST7251	VIC	0.06	**DETECTED**		
**Clonal Complex 15 (n=41)**						
ISTOP-7	ST15	WA	0.06	-		
ISTOP-46	ST15	NSW	0.12	-		
ISTOP-59	ST15	NSW	0.06	-		
ISTOP-79	ST15	ACT	0.06	-	SXT^R	*dfrG*

Isolate	ST	STATE	Vitek 2<br>Pen MIC mg/L	*blaZ*	Antibiogram	Resistance Genes
ISTOP-109	ST15	QLD	0.12	-		
ISTOP-119	ST15	WA	0.12	-		
ISTOP-129	ST15	NSW	0.06	-		
ISTOP-134	ST15	NSW	0.12	-	Cli^R, Ery^R	*erm(C)*
ISTOP-137	ST15	VIC	0.06	-		
ISTOP-221	ST15	TAS	≤0.03	-		
ISTOP-225	ST15	WA	0.12	-		
ISTOP-235	ST15	WA	0.06	-		
ISTOP-236	ST15	WA	0.12	-		
ISTOP-299	ST15	VIC	0.12	-		
ISTOP-309	ST15	VIC	0.12	-		
ISTOP-348	ST15	WA	0.06	-		
ISTOP-356	ST15	WA	0.06	-		
ISTOP-379	ST15	ACT	0.06	-		
ISTOP-438	ST15	NSW	0.12	**DETECTED**		
ISTOP-20	ST333	VIC	0.06	-		
ISTOP-50	ST582	NSW	0.12	**DETECTED**	Cip^R,FA^R	*fusC,* GrlA S80F and GyrA S84 mutations
ISTOP-153	ST582	TAS	0.12	**DETECTED**		
ISTOP-157	ST582	NSW	0.12	**DETECTED**		
ISTOP-223	ST582	WA	0.06	**DETECTED**		
ISTOP-306	ST582	Qld	0.12	**DETECTED**		
ISTOP-318	ST582	VIC	0.12	**DETECTED**		
ISTOP-324	ST582	VIC	0.06	**DETECTED**		
ISTOP-447	ST582	NSW	0.06	**DETECTED**		
ISTOP-462	ST582	QLD	0.12	**DETECTED**		
ISTOP-465	ST582	QLD	0.12	**DETECTED**		
ISTOP-475	ST582	QLD	0.12	**DETECTED**		
ISTOP-27	ST3911	VIC	0.06	**DETECTED**		
ISTOP-171	ST3911	WA	0.12	**DETECTED**		
ISTOP-210	ST3911	QLD	0.06	**DETECTED**		
ISTOP-276	ST3911	NSW	0.12	**DETECTED**		
ISTOP-295	ST3911	VIC	0.12	**DETECTED**		
ISTOP-115	ST5059	NSW	0.12	**DETECTED**		
ISTOP-243	ST7264	SA	0.06	-		
ISTOP-300	ST7273	QLD	0.12	**DETECTED**		
ISTOP-401	ST7283	VIC	0.12	**DETECTED**		
ISTOP-445	ST7286	NSW	≤0.03	-		
**Clonal Complex 20 (n=3)**						
ISTOP-85	ST20	TAS	0.12	-		
ISTOP-342	ST20	TAS	0.12	-		
ISTOP-427	ST20	SA	0.12	**DETECTED**		
**Clonal Complex 22 (n=8)**						
ISTOP-266	ST22	ACT	0.06	-		
ISTOP-278	ST22	NSW	0.12	-		
ISTOP-347	ST22	WA	0.12	-		
ISTOP-360	ST22	WA	0.06	**DETECTED**		
ISTOP-383	ST22	VIC	0.12	-		

Isolate	ST	STATE	Vitek 2 Pen MIC mg/L	*blaZ*	Antibiogram	Resistance Genes
ISTOP-384	ST22	VIC	≤0.03	-		
ISTOP-292	ST7272	QLD	≤0.03	-		
ISTOP-435	ST7285	TAS	0.06	-		
**Clonal Complex 25 (n=1)**						
ISTOP-325	ST7276	VIC	≤0.03	**DETECTED**		
**Clonal Complex 30 (n=7)**						
ISTOP-311	ST30	VIC	0.06	-	Cip^R,	*dfrG*, GrlA S80F and GyrA S84 mutations
ISTOP-319	ST30	VIC	0.12	**DETECTED**		
ISTOP-42	ST34	VIC	0.06	**DETECTED**		
ISTOP-101	ST34	VIC	≤0.03	-	Cli^R, Ery^R	*ermC*
ISTOP-263	ST34	ACT	0.06	**DETECTED**		
ISTOP-264	ST34	ACT	0.06	**DETECTED**		
ISTOP-296	ST39	VIC	≤0.03	-	Ery^R	*ant(9)-Ia_1, erm(A)*
**Clonal Complex 45 (n=47)**						
ISTOP-31	ST45	QLD	0.06	-		
ISTOP-68	ST45	WA	0.06	-		
ISTOP-70	ST45	NSW	0.06	-		
ISTOP-88	ST45	TAS	0.12	**DETECTED**		
ISTOP-94	ST45	SA	0.12	-		
ISTOP-122	ST45	WA	0.06	-		
ISTOP-123	ST45	SA	0.12	-		
ISTOP-139	ST45	VIC	≤0.03	-		
ISTOP-149	ST45	ACT	0.06	-		
ISTOP-160	ST45	NSW	0.06	-		
ISTOP-170	ST45	WA	≤0.03	-		
ISTOP-189	ST45	NSW	0.06	-		
ISTOP-216	ST45	TAS	0.06	-		
ISTOP-230	ST45	WA	0.06	-		
ISTOP-260	ST45	ACT	0.06	-		
ISTOP-279	ST45	NSW	0.12	-		
ISTOP-283	ST45	NSW	0.06	-		
ISTOP-337	ST45	WA	0.06	-		
ISTOP-366	ST45	NSW	0.06	-		
ISTOP-373	ST45	VIC	0.06	-		
ISTOP-376	ST45	WA	0.06	-		
ISTOP-386	ST45	QLD	0.06	-	Cli^R, Ery^R	*ant(4')-Ia, aadD1, erm(C), mph(C)*
ISTOP-426	ST45	SA	0.06	-		
ISTOP-439	ST45	NT	0.12	-		
ISTOP-454	ST45	NSW	0.06	-		
ISTOP-455	ST45	NSW	0.06	-		
ISTOP-456	ST45	NSW	≤0.03	-		
ISTOP-468	ST45	QLD	≤0.03	-		
ISTOP-470	ST45	QLD	0.06	-		
ISTOP-473	ST45	QLD	≤0.03	-		
ISTOP-26	ST508	NSW	≤0.03	-		
ISTOP-35	ST508	NSW	0.12	-		

Isolate	ST	STATE	Vitek 2 Pen MIC mg/L	*blaZ*	Antibiogram	Resistance Genes
ISTOP-154	ST508	TAS	≤0.03	-		
ISTOP-163	ST508	NSW	0.06	-		
ISTOP-169	ST508	WA	0.06	-		
ISTOP-253	ST508	WA	0.12	-		
ISTOP-269	ST508	NSW	0.125	-		
ISTOP-336	ST508	SA	0.125	-		
ISTOP-408	ST508	VIC	0.06	-		
ISTOP-75	ST7254	NSW	≤0.03	-		
ISTOP-84	ST7255	TAS	0.12	**DETECTED**		
ISTOP-147	ST7258	ACT	0.06	-		
ISTOP-203	ST7261	SA	≤0.03	-		
ISTOP-268	ST7268	NSW	0.06	-		
ISTOP-346	ST7279	VIC	0.06	-		
ISTOP-419	ST7284	NSW	0.12	-		
ISTOP-463	ST7289	QLD	0.12	-		
**Clonal Complex 59 (n=9)**						
ISTOP-67	ST59	WA	0.12	-		
ISTOP-77	ST59	ACT	0.06	-		
ISTOP-343	ST59	TAS	0.06	-		
ISTOP-368	ST59	NSW	0.12	-	Ery^R	*ant(9)-Ia_1, erm(A)*
ISTOP-403	ST59	VIC	0.12	-		
ISTOP-437	ST59	NSW	0.06	-		
ISTOP-256	ST87	WA	0.12	-		
ISTOP-186	ST1224	NSW	≤0.03	-		
ISTOP-354	ST7280	WA	0.06	-		
**Clonal Complex 80 (n=1)**						
ISTOP-199	ST80	VIC	0.12	-		
**Clonal Complex 88 (n=17)**						
ISTOP-47	ST78	NSW	0.12	-	Ery^R	*ant(9)-Ia_1, erm(A)*
ISTOP-76	ST78	ACT	0.12	-	Ery^R	*ant(9)-Ia_1, erm(A)*
ISTOP-182	ST78	VIC	0.12	-		
ISTOP-217	ST78	TAS	0.06	-		*ant(9)-Ia_1, erm(A)*
ISTOP-261	ST78	ACT	0.06	-	Ery^R	*ant(9)-Ia_1, erm(A)*
ISTOP-312	ST78	NSW	0.06	-		
ISTOP-471	ST78	QLD	0.06	-		
ISTOP-8	ST88	WA	0.12	-	FA^R	*fusA* H457Q mutation
ISTOP-71	ST88	NSW	0.06	-		
ISTOP-120	ST88	WA	0.12	-	FA^R	*fusA* H457Q mutation
ISTOP-135	ST88	NSW	0.12	-		
ISTOP-175	ST88	VIC	0.06	-		
ISTOP-308	ST88	VIC	0.12	-		
ISTOP-333	ST88	NSW	0.12	-		
ISTOP-441	ST88	NT	0.12	-		
ISTOP-444	ST88	NT	≤0.03	-		
ISTOP-355	ST7281	WA	≤0.03	-	Ery^R	*ant(9)-Ia_1, erm(A)*
**Clonal Complex 97 (n=48)**						
ISTOP-37	ST97	NSW	0.06	-		
ISTOP-54	ST97	NSW	0.12	-		

Isolate	ST	STATE	Vitek 2 Pen MIC mg/L	*blaZ*	Antibiogram	Resistance Genes
ISTOP-57	ST97	NSW	0.06	-		
ISTOP-65	ST97	NSW	0.12	-		
ISTOP-73	ST97	NSW	0.12	-		
ISTOP-80	ST97	NSW	0.12	-		
ISTOP-96	ST97	VIC	≤0.03	-		
ISTOP-138	ST97	VIC	0.06	-		
ISTOP-172	ST97	VIC	0.06	-		
ISTOP-198	ST97	SA	0.12	-		
ISTOP-211	ST97	QLD	0.06	-	FA^R	
ISTOP-242	ST97	SA	0.12	-		
ISTOP-245	ST97	WA	0.12	-	Ery^R	*ant(9)-Ia_1, erm(A)*
ISTOP-250	ST97	WA	0.12	-		
ISTOP-280	ST97	NSW	0.12	-		
ISTOP-291	ST97	QLD	0.06	-		
ISTOP-298	ST97	VIC	0.12	-		
ISTOP-307	ST97	VIC	≤0.03	-		
ISTOP-320	ST97	VIC	0.12	-		
ISTOP-335	ST97	SA	≤0.03	-		
ISTOP-352	ST97	WA	0.12	-		
ISTOP-358	ST97	WA	0.06	-		
ISTOP-359	ST97	WA	≤0.03	-		
ISTOP-367	ST97	NSW	0.12	-		
ISTOP-385	ST97	QLD	0.06	-		
ISTOP-414	ST97	NSW	0.06	-		
ISTOP-416	ST97	NSW	0.12	-		
ISTOP-420	ST97	NSW	0.12	-		
ISTOP-422	ST97	NSW	0.12	-		
ISTOP-434	ST97	TAS	0.12	-		
ISTOP-449	ST97	NSW	0.12	-		
ISTOP-469	ST97	QLD	0.06	-		
ISTOP-474	ST97	QLD	0.06	-		
ISTOP-477	ST97	QLD	0.06	-		
ISTOP-10	ST953	WA	0.12	-		
ISTOP-45	ST953	NSW	0.06	-		
ISTOP-58	ST953	NSW	0.12	-		
ISTOP-126	ST953	SA	0.12	-		
ISTOP-234	ST953	WA	≤0.03	-		
ISTOP-237	ST953	WA	0.12	-		
ISTOP-255	ST953	NSW	0.06	-		
ISTOP-273	ST953	TAS	0.12	-		*lnu(A)*
ISTOP-392	ST953	NSW	≤0.03	-		
ISTOP-443	ST953	NT	≤0.03	-		
ISTOP-5	ST1179	WA	0.12	-	Tet^R	*tet(K)*
ISTOP-404	ST1179	VIC	0.12	-		
ISTOP-99	ST7256	VIC	0.06	-		
ISTOP-344	ST7278	TAS	0.12	-		
**Clonal Complex 101 (n=16)**						
ISTOP-136	ST101	TAS	0.06	-		

Isolate	ST	STATE	Vitek 2 Pen MIC mg/L	*blaZ*	Antibiogram	Resistance Genes
ISTOP-174	ST101	VIC	0.12	**DETECTED**		
ISTOP-192	ST101	VIC	0.12	-		
ISTOP-284	ST101	NSW	0.12	-		
ISTOP-289	ST101	NSW	0.06	-		
ISTOP-294	ST101	VIC	0.06	-		
ISTOP-332	ST101	NSW	0.06	-		
ISTOP-340	ST101	NSW	0.12	-		
ISTOP-361	ST101	NSW	0.12	-		
ISTOP-393	ST101	NSW	≤0.03	-		
ISTOP-411	ST101	NSW	0.06	-		
ISTOP-484	ST101	QLD	0.12	-		
ISTOP-72	ST1155	QLD	0.12	-		
ISTOP-168	ST1155	WA	0.06	-		
ISTOP-133	ST7257	NSW	0.06	-		
ISTOP-305	ST7274	QLD	0.06	-		
**Clonal Complex 188 (n=23)**						
ISTOP-2	ST188	NSW	0.06	-		
ISTOP-24	ST188	VIC	0.12	-		
ISTOP-25	ST188	NSW	≤0.03	-		
ISTOP-44	ST188	NSW	0.12	-		
ISTOP-52	ST188	NSW	0.12	-		
ISTOP-56	ST188	NSW	0.06	-		
ISTOP-92	ST188	SA	0.06	-		
ISTOP-112	ST188	NSW	0.06	-		
ISTOP-155	ST188	TAS	0.12	-		*aph(3')-IIIa*
ISTOP-156	ST188	NSW	0.06	-		
ISTOP-165	ST188	NSW	0.12	-		
ISTOP-205	ST188	NSW	0.12	-		
ISTOP-317	ST188	VIC	0.12	-		
ISTOP-417	ST188	NSW	≤0.03	-		
ISTOP-440	ST188	NT	0.12	-		
ISTOP-446	ST188	NSW	0.06	-		
ISTOP-458	ST188	QLD	0.12	**DETECTED**		
ISTOP-460	ST188	QLD	0.06	-		
ISTOP-464	ST188	QLD	0.06	-		
ISTOP-467	ST188	QLD	0.06	-		
ISTOP-472	ST188	QLD	0.06	-		
ISTOP-185	ST7259	NSW	0.06	-		
ISTOP-287	ST7271	NSW	0.125	-		
**Clonal Complex 291 (n=4)**						
ISTOP-55	ST291	NSW	0.12	-		
ISTOP-326	ST291	VIC	≤0.03	-	Cip^R	GrlA S80F and GyrA 84 mutations
ISTOP-418	ST291	NSW	0.06	-		
ISTOP-453	ST7287	NSW	0.12	-		
**Clonal Complex 361 (n=5)**						
ISTOP-108	ST672	NSW	≤0.03	-	Cip^R	GrlA S80F and GyrA 84 mutations

Isolate	ST	STATE	Vitek 2 Pen MIC mg/L	*blaZ*	Antibiogram	Resistance Genes
ISTOP-131	ST672	ACT	0.06	-		
ISTOP-151	ST672	WA	0.12	-		
ISTOP-222	ST672	QLD	0.06	-		
ISTOP-302	ST672	NSW	0.06	-		
**Clonal Complex 398 (n=16)**						
ISTOP-11	ST398	WA	0.06	-		
ISTOP-16	ST398	VIC	0.06	-	Ery^R	*erm(T)*
ISTOP-97	ST398	VIC	≤0.03	-		*erm(T)*
ISTOP-132	ST398	NSW	0.12	-	Ery^R	*erm(T)*
ISTOP-213	ST398	VIC	0.12	-	Ery^R	*erm(T)*
ISTOP-214	ST398	VIC	0.06	-		*erm(T)*
ISTOP-226	ST398	WA	0.06	-	Ery^R	*erm(T)*
ISTOP-240	ST398	SA	0.06	-	Ery^R	*erm(T)*
ISTOP-304	ST398	QLD	0.12	-	Ery^R	*erm(T)*
ISTOP-362	ST398	NSW	≤0.03	-	Cli^R, Ery^R	*erm(C)*
ISTOP-363	ST398	NSW	0.06	-	Ery^R	*erm(T)*
ISTOP-448	ST398	NSW	≤0.03	-	Ery^R	*erm(T)*
ISTOP-98	ST3332	VIC	≤0.03	-		
ISTOP-100	ST3332	VIC	≤0.03	-		
ISTOP-323	ST7275	NSW	0.12	-	Ery^R	*erm(T)*
ISTOP-328	ST7277	NSW	0.06	-	Ery^R	*erm(T)*
**Singletons (n=7)**						
ISTOP-146	ST425	ACT	0.06	-		
ISTOP-162	ST2867	NSW	0.12	-		
ISTOP-208	ST2867	QLD	0.12	-		
ISTOP-288	ST2867	NSW	0.06	-		*ant(4')-Ia, aadD1*
ISTOP-190	ST5491	VIC	≤0.03	-		
ISTOP-277	ST7270	NSW	0.06	-		
ISTOP-483	ST573	QLD	0.12	-		
