# Supplementary material for: Molecular Epidemiology of Penicillin-Susceptible Staphylococcus aureus Bacteremia in Australia and Reliability of Diagnostic Phenotypic Susceptibility Methods to Detect Penicillin Susceptibility
Source: Microorganisms. 2022 Aug 15;10(8):1650. doi: 10.3390/microorganisms10081650 (PMC9413241; doi:10.3390/microorganisms10081650)
Supplement: Supplementary file 1 [file microorganisms-10-01650-s001.zip › Supplementary Table 3.pdf]

Table S3: Multilocus sequence type, origin, Vitek® 2 penicillin minimum inhibitory concentration, detection of *blaZ*, *agr* type, capsule type, *spa* type and virulence genes on 470 penicillin-susceptible *Staphylococcus aureus* identified in the Australian Group for Antimicrobial Resistance's 2020 Australian *Staphylococcus aureus* Sepsis Outcome Program

Isolate	ST	STATE	Vitek 2 Pen MIC mg/L	*blaZ*	*agr* type	Capsule type	*spa*	Enterotoxins	Other Virulence Genes	IEC (Type)
**Clonal Complex 1 (n=17)**										
ISTOP-48	ST1	NSW	0.12	-	III	8	t127	*seh, sek+seq, selx*	-	*sak, scn* (E)
ISTOP-127	ST1	SA	0.12	-	III	8	t177	*sea, seh, sek+seq, selx*	-	*sea, sak, scn* (D)
ISTOP-141	ST1	VIC	≤0.03	-	III	8	t2207	*seh, sek+seq, selx*	-	*sak, scn* (E)
ISTOP-152	ST1	TAS	≤0.03	-	III	8	t127	*seh, selx*	-	-
ISTOP-212	ST1	QLD	0.06	-	III	8	t127	*seh, sek+seq, selx*	-	*sak, scn* (E)
ISTOP-219	ST1	TAS	0.06	-	III	8	t127	*sea, seh, sek+seq, selx*	-	*sea, sak, scn* (D)
ISTOP-281	ST1	NSW	0.06	-	III	8	t127	*sea, seh, sek+seq, selx*	-	*sea, sak, scn* (D)
ISTOP-330	ST1	NSW	≤0.03	-	III	8	t127	*seh, sek+seq, selx*	-	*sak, scn* (E)
ISTOP-364	ST1	NSW	0.12	-	III	8	t127	*seh, sek+seq, selx*	-	*sak, scn* (E)
ISTOP-396	ST1	WA	0.12	-	III	8	t127	*sea, seb, seh, sek+seq, selx*	-	*sea, sak, scn* (D)
ISTOP-407	ST1	VIC	0.12	-	III	8	t174	*sea, seh, sek+seq, selx*	-	*sea, sak, scn* (D)
ISTOP-409	ST1	NSW	0.06	-	III	8	t127	*sea, seh, sek+seq, selx*	*lukF/S-PVL*	*sea, sak, scn* (D)
ISTOP-433	ST1	TAS	0.06	-	III	8	t1909	*sea, seh, sek+seq, selx*	--	*sea, sak, scn* (D)
ISTOP-442	ST1	NT	≤0.03	-	III	8	t127	*sea, seh, sek+sel, selx*	-	*sea, sak, scn* (D)
ISTOP-130	ST3949	NSW	0.12	-	III	8	t2207	*sea, sec+sel, seh, sek+seq, selx*	-	*sea, sak, scn* (D)
ISTOP-21	ST4100	VIC	≤0.03	-	III	8	Unknown	*selx*	*etA*	*sak, scn* (E)
ISTOP-69	ST4100	NSW	0.06		III	8	Unknown	*selx*	-	*sak, scn* (E)
**Clonal Complex 5 (n=150)**										
ISTOP-3	ST5	WA	0.06	-	II	5	t002	*egc-cluster (seg+ sei+sem+sen+seo+selw), selx*	-	*sak, chp, scn* (B)
ISTOP-9	ST5	WA	≤0.03	-	II	5	t1227	*egc-cluster (seg+ sei+sem+sen+seo+selw),*	-	*sak, scn* (E)

Isolate	ST	STATE	Vitek 2 Pen MIC mg/L	*blaZ*	*agr* type	Capsule type	*spa*	Enterotoxins	Other Virulence Genes	IEC (Type)
								selx		
ISTOP-15	ST5	VIC	0.06	-	II	5	t1560	*seb, sep, egc-cluster (seg+ sei+sem+sen+seo+selw), selx*	-	*sep, sak, chp, scn (F)*
ISTOP-18	ST5	VIC	0.06	-	II	5	t002	*egc-cluster (seg+ sei+sem+sen+seo+selw), selx*	-	*sak, chp, scn* (B)
ISTOP-19	ST5	VIC	0.12	-	II	5	t306	*egc-cluster (seg+ sei+sem+sen+seo+selw), selx*	-	*sak, chp, scn* (B)
ISTOP-22	ST5	VIC	≤0.03	-	II	5	t1265	*egc-cluster (seg+ sei+sem+sen+seo+selw), selx*	-	-
ISTOP-28	ST5	VIC	0.06	-	II	5	t002	*sep, egc-cluster (seg+ sei+sem+sen+seo+selw), selx*	-	*sep, sak, chp, scn (F)*
ISTOP-32	ST5	QLD	0.12	-	II	5	t002	*egc-cluster (seg+ sei+sem+sen+seo+selw), selx*	-	*sak, scn* (E)
ISTOP-33	ST5	NSW	0.06	-	II	5	t002	*sep, egc-cluster (seg+ sei+sem+sen+seo+selw), selx*	-	*sep, sak, chp, scn (F)*
ISTOP-36	ST5	NSW	0.06	-	II	5	t2049	*sea, egc-cluster (seg+ sei+sem+sen+seo+selw), selx*	-	*sea, sak, scn (D)*
ISTOP-38	ST5	TAS	0.12	-	II	5	t002	*sep, egc-cluster (seg+ sei+sem+sen+seo+selw), selx*	-	*sep, sak, chp, scn (F)*
ISTOP-49	ST5	NSW	≤0.03	-	II	5	t548	*sep, egc-cluster (seg+ sei+sem+sen+seo+selw), selx*	-	*sep, sak, scn* (G)
ISTOP-60	ST5	VIC	0.06	-	II	5	t002	*egc-cluster (seg+ sei+sem+sen+seo+selw), selx*	-	*sak, scn* (E)
ISTOP-63	ST5	VIC	0.06	-	II	5	t002	*sep, egc-cluster (seg+ sei+sem+sen+seo+selw), selx*	-	*sep, sak, chp, scn (F)*
ISTOP-64	ST5	NSW	0.12	-	II	5	t002	*sep, egc-cluster (seg+ sei+sem+sen+seo+selw), selx*	-	*sep, sak, chp, scn (F)*
ISTOP-66	ST5	NSW	0.06	-	II	5	t1265	*egc-cluster (seg+ sei+sem+sen+seo+selw), selx*	-	*sak, chp, scn* (B)

Isolate	ST	STATE	Vitek 2 Pen MIC mg/L	*blaZ*	*agr* type	Capsule type	*spa*	Enterotoxins	Other Virulence Genes	IEC (Type)
ISTOP-81	ST5	NSW	0.12	-	II	5	Unknown	*egc-cluster (seg+ sei+sem+sen+seo+selw), selx*	-	-
ISTOP-82	ST5	TAS	≤0.03	-	II	5	t002	*sep, egc-cluster (seg+ sei+sem+sen+seo+selw), selx*	-	*sep, sak, chp, scn (F)*
ISTOP-83	ST5	TAS	0.06	-	II	5	t1265	*egc-cluster (seg+ sei+sem+sen+seo+selw), selx*	-	*sak, chp, scn* (B)
ISTOP-89	ST5	TAS	0.06	-	II	5	t1265	*egc-cluster (seg+ sei+sem+sen+seo+selw), selx*	-	*sak, chp, scn* (B)
ISTOP-90	ST5	SA	0.12	-	II	5	t214	*sep, egc-cluster (seg+ sei+sem+sen+seo+selw), selx*	-	*sep, sak, chp, scn (F)*
ISTOP-93	ST5	SA	0.12	-	II	5	t002	*sep, egc-cluster (seg+ sei+sem+sen+seo+selw), selx*	-	*sep, sak, chp, scn (F)*
ISTOP-95	ST5	SA	0.06	-	II	5	t002	*egc-cluster (seg+ sei+sem+sen+seo+selw), selx*	-	*sak, chp, scn* (B)
ISTOP-102	ST5	VIC	≤0.03	-	II	5	t3597	*sep, egc-cluster (seg+ sei+sem+sen+seo+selw), selx*	-	*sep, sak, chp, scn (F)*
ISTOP-103	ST5	SA	0.06	-	II	5	t9394	*sep, egc-cluster (seg+ sei+sem+sen+seo+selw), selx*	-	*sep, sak, chp, scn (F)*
ISTOP-106	ST5	NSW	0.06	-	II	5	t2958	*sep, egc-cluster (seg+ sei+sem+sen+seo+selw), selx*	-	*sep, sak, scn (G)*
ISTOP-107	ST5	NSW	0.12	-	II	5	t002	*egc-cluster (seg+ sei+sem+sen+seo+selw), selx*	-	*sak, scn (E)*
ISTOP-110	ST5	QLD	≤0.03	-	II	5	t002	*sep, egc-cluster (seg+ sei+sem+sen+seo+selw), selx*	-	*sep, sak, chp, scn (F)*
ISTOP-111	ST5	QLD	0.06	-	II	5	t111	*sea, egc-cluster (seg+ sei+sem+sen+seo+selw), selx*	-	*sea, sak, chp, scn (A)*
ISTOP-113	ST5	NSW	≤0.03	-	II	5	t1265	*egc-cluster (seg+ sei+sem+sen+seo+selw), selx*	-	*sak, chp, scn* (B)
ISTOP-116	ST5	SA	0.12	-	II	5	t002	*sep, egc-cluster*	-	*sep, sak, chp, scn (F)*

Isolate	ST	STATE	Vitek 2 Pen MIC mg/L	*blaZ*	*agr* type	Capsule type	*spa*	Enterotoxins	Other Virulence Genes	IEC (Type)
								(seg+ sei+sem+sen+seo+selw), selx		
ISTOP-118	ST5	WA	0.12	-	II	5	t002	*egc-cluster (seg+ sei+sem+sen+seo+selw), selx*	-	-
ISTOP-124	ST5	SA	0.12	-	II	5	t002	*egc-cluster (seg+ sei+sem+sen+seo+selw), selx*	-	*sak, scn* (E)
ISTOP-128	ST5	SA	0.12	-	II	5	t002	*sep, egc-cluster (seg+ sei+sem+sen+seo+selw), selx*	-	*sep, sak, chp, scn (F)*
ISTOP-142	ST5	SA	0.06	-	II	5	t688	*sep, egc-cluster (seg+ sei+sem+sen+seo+selw), selx*	-	*sep, sak, scn* (G)
ISTOP-145	ST5	ACT	0.06	-	II	5	t17058	*egc-cluster (seg+ sei+sem+sen+seo+selw), selx*	-	*sak, chp, scn* (B)
ISTOP-148	ST5	ACT	0.06	-	II	5	t8241	*egc-cluster (seg+ sei+sem+sen+seo+selw), selx*	-	*sak, chp, scn* (B)
ISTOP-158	ST5	NSW	≤0.03	**DETECTED**	II	5	t1265	*sed+selj+ser, egc-cluster (seg+ sei+sem+sen+seo+selw), selx*	-	*sak, chp, scn* (B)
ISTOP-159	ST5	NSW	0.06	-	II	5	t7186	*egc-cluster (seg+ sei+sem+sen+seo+selw), selx*	-	*sak, chp, scn* (B)
ISTOP-161	ST5	NSW	0.12	-	II	5	t002	*egc-cluster (seg+ sei+sem+sen+seo+selw), selx*	-	*sak, chp, scn* (B)
ISTOP-166	ST5	NSW	0.12	-	II	5	t2595	*egc-cluster (seg+ sei+sem+sen+seo+selw), selx*	-	*sak, chp, scn* (B)
ISTOP-167	ST5	NSW	0.12	-	II	5	t002	*egc-cluster (seg+ sei+sem+sen+seo+selw), selx*	-	*sak, scn* (E)
ISTOP-173	ST5	VIC	0.12	**DETECTED**	II	5	t002	*egc-cluster (seg+ sei+sem+sen+seo+selw), selx*	-	*sak, chp, scn* (B)
ISTOP-176	ST5	VIC	0.12	-	II	5	t002	*sed+selj+ser, egc-cluster (seg+ sei+sem+sen+seo+selw), selx*	-	*sak, scn* (E)
ISTOP-178	ST5	VIC	0.06	-	II	5	t442	*egc-cluster (seg+ sei+sem+sen+seo+selw), selx*	-	*sak, chp, scn* (B)

Isolate	ST	STATE	Vitek 2 Pen MIC mg/L	*blaZ*	*agr* type	Capsule type	*spa*	Enterotoxins	Other Virulence Genes	IEC (Type)
ISTOP-179	ST5	VIC	0.12	-	II	5	t002	*egc-cluster (seg+ sei+sem+sen+seo+selw), selx*	-	*sak, chp, scn* (B)
ISTOP-180	ST5	VIC	0.12	-	II	5	t062	*sep, egc-cluster (seg+ sei+sem+sen+seo+selw), selx*	-	*sep, sak, chp, scn (F)*
ISTOP-184	ST5	NSW	≤0.03	-	II	5	t1265	*egc-cluster (seg+ sei+sem+sen+seo+selw), selx*	-	*sak, chp, scn* (B)
ISTOP-187	ST5	NSW	0.12	**DETECTED**	II	5	t010	*sed+selj+ser, sep, egc-cluster (seg+ sei+sem+sen+seo+selw), selx*	-	*sep, sak, chp, scn (F)*
ISTOP-188	ST5	NSW	0.06	**DETECTED**	II	5	t1265	*sed+selj+ser, egc-cluster (seg+ sei+sem+sen+seo+selw), selx*	-	*sak, chp, scn* (B)
ISTOP-191	ST5	VIC	0.12	-	II	5	t002	*sep, egc-cluster (seg+ sei+sem+sen+seo+selw), selx*	-	*sep, sak, chp, scn (F)*
ISTOP-193	ST5	SA	0.06	-	II	5	t002	*sea, egc-cluster (seg+ sei+sem+sen+seo+selw), selx*	-	*sea, sak, scn (D)*
ISTOP-200	ST5	VIC	0.12	-	II	5	t002	*sep, egc-cluster (seg+ sei+sem+sen+seo+selw), selx*	*tst*	*sep, sak, chp, scn (F)*
ISTOP-201	ST5	VIC	0.06	-	II	5	t442	*egc-cluster (seg+ sei+sem+sen+seo+selw), selx*	-	*sak, scn* (E)
ISTOP-206	ST5	NSW	0.06	-	II	5	t002	*sed+selj+ser, sep, egc-cluster (seg+ sei+sem+sen+seo+selw), selx*	-	*sep, sak, chp, scn (F)*
ISTOP-207	ST5	QLD	0.12	**DETECTED**	II	5	Unknown	*sed+selj+ser, egc-cluster (seg+ sei+sem+sen+seo+selw), selx*	-	*sak, chp, scn* (B)
ISTOP-209	ST5	QLD	0.06	-	II	5	t002	*sep, egc-cluster (seg+ sei+sem+sen+seo+selw), selx*	-	*sep, sak, chp, scn (F)*
ISTOP-218	ST5	TAS	0.12	-	II	5	t010	*egc-cluster (seg+ sei+sem+sen+seo+selw), selx*	-	*sak, chp, scn* (B)
ISTOP-224	ST5	WA	0.12	-	II	5	t002	*sea, sed+selj+ser, egc-cluster (seg+ sei+sem+sen+seo+selw), selx*	-	*sea, sak, scn (D)*
ISTOP-227	ST5	WA	0.06	-	II	5	t002	*egc-cluster (seg+ sei+sem+sen+seo+selw),*	-	*sak, chp, scn* (B)

Isolate	ST	STATE	Vitek 2 Pen MIC mg/L	*blaZ*	*agr* type	Capsule type	*spa*	Enterotoxins	Other Virulence Genes	IEC (Type)
								selx		
ISTOP-231	ST5	WA	0.12	-	II	5	t002	*egc-cluster (seg+ sei+sem+sen+seo+selw), selx*	-	*sak, chp, scn* (B)
ISTOP-232	ST5	WA	0.12	-	II	5	t5081	*sed+selj+ser, sep, egc-cluster (seg+ sei+sem+sen+seo+selw), selx*	-	*sep, sak, chp, scn (F)*
ISTOP-233	ST5	WA	0.12	**DETECTED**	II	5	t179	*sed+selj+ser, egc-cluster (seg+ sei+sem+sen+seo+selw), selx*	-	*sak, chp, scn* (B)
ISTOP-241	ST5	SA	≤0.03	-	II	5	t002	*sep, egc-cluster (seg+ sei+sem+sen+seo+selw), selx*	-	*sep, sak, chp, scn (F)*
ISTOP-249	ST5	WA	0.06	-	II	5	t088	*egc-cluster (seg+ sei+sem+sen+seo+selw), selx*	-	*sak, scn* (E)
ISTOP-252	ST5	WA	0.12	-	II	5	t045	*sep, egc-cluster (seg+ sei+sem+sen+seo+selw), selx*	-	*sep, sak, chp, scn (F)*
ISTOP-262	ST5	ACT	≤0.03	-	II	5	t002	*sep, egc-cluster (seg+ sei+sem+sen+seo+selw), selx*	-	*sep, sak, chp, scn (F)*
ISTOP-265	ST5	ACT	0.12	-	II	5	t570	*egc-cluster (seg+ sei+sem+sen+seo+selw), selx*	-	*sak, scn* (E)
ISTOP-267	ST5	ACT	0.06	-	II	5	t3660	*egc-cluster (seg+ sei+sem+sen+seo+selw), selx*	-	*sak, chp, scn* (B)
ISTOP-270	ST5	NSW	0.06	-	II	5	t1265	*egc-cluster (seg+ sei+sem+sen+seo+selw), selx*	-	*sak, chp, scn* (B)
ISTOP-272	ST5	TAS	≤0.03	-	II	5	t002	*sep, egc-cluster (seg+ sei+sem+sen+seo+selw), selx*	*tst*	*sep, sak, chp, scn (F)*
ISTOP-275	ST5	TAS	≤0.03	-	II	5	t3065	*egc-cluster (seg+ sei+sem+sen+seo+selw), selx*	-	*sak, chp, scn* (B)
ISTOP-282	ST5	NSW	0.12	-	II	5	t1265	*egc-cluster (seg+ sei+sem+sen+seo+selw), selx*	-	*sak, chp, scn* (B)
ISTOP-290	ST5	NSW	0.06	-	II	5	t002	*egc-cluster (seg+ sei+sem+sen+seo+selw), selx*	-	*sak, chp, scn* (B)

Isolate	ST	STATE	Vitek 2 Pen MIC mg/L	*blaZ*	*agr* type	Capsule type	*spa*	Enterotoxins	Other Virulence Genes	IEC (Type)
ISTOP-301	ST5	QLD	0.06	-	II	5	t010	*sep, egc-cluster (seg+ sei+sem+sen+seo+selw), selx*	-	*sep, sak, chp, scn (F)*
ISTOP-303	ST5	QLD	0.06	-	II	5	t002	*sep, egc-cluster (seg+ sei+sem+sen+seo+selw), selx*	-	*sep, sak, chp, scn (F)*
ISTOP-310	ST5	VIC	0.06	-	II	5	t1265	*egc-cluster (seg+ sei+sem+sen+seo+selw), selx*	-	*sak, chp, scn* (B)
ISTOP-313	ST5	NSW	0.06	-	II	5	t179	*sep, egc-cluster (seg+ sei+sem+sen+seo+selw), selx*	-	*sep, sak, scn* (G)
ISTOP-315	ST5	VIC	0.06	-	II	5	t045	*egc-cluster (seg+ sei+sem+sen+seo+selw), selx*	-	*sak, chp, scn* (B)
ISTOP-316	ST5	VIC	0.12	-	II	5	t179	*sed+selj+ser, egc-cluster (seg+ sei+sem+sen+seo+selw), selx*	-	*sak, chp, scn* (B)
ISTOP-321	ST5	NSW	0.06	-	II	5	t1265	*egc-cluster (seg+ sei+sem+sen+seo+selw), selx*	-	*sak, chp, scn* (B)
ISTOP-322	ST5	NSW	0.06	-	II	5	t1265	*egc-cluster (seg+ sei+sem+sen+seo+selw), selx*	-	*sak, chp, scn* (B)
ISTOP-327	ST5	VIC	≤0.03	-	II	5	t010	*egc-cluster (seg+ sei+sem+sen+seo+selw), selx*	-	*sak, chp, scn* (B)
ISTOP-329	ST5	NSW	0.06	-	II	5	t1265	*egc-cluster (seg+ sei+sem+sen+seo+selw), selx*	-	*sak, chp, scn* (B)
ISTOP-334	ST5	SA	0.06	-	II	5	t1265	*sep, egc-cluster (seg+ sei+sem+sen+seo+selw), selx*	-	*sep, sak, scn* (G)
ISTOP-339	ST5	NSW	0.06	-	II	5	t1265	*sed+selj+ser, egc-cluster (seg+ sei+sem+sen+seo+selw), selx*	-	*sak, chp, scn* (B)
ISTOP-350	ST5	WA	0.12	-	II	5	t002	*egc-cluster (seg+ sei+sem+sen+seo+selw), selx*	-	*sak, scn* (E)
ISTOP-351	ST5	WA	0.12	-	II	5	t179	*sep, egc-cluster (seg+ sei+sem+sen+seo+selw), selx*	-	*sep, sak, scn* (G)
ISTOP-353	ST5	WA	≤0.03	-	II	5	t002	*sea, egc-cluster*	-	*sea, sak, chp, scn (A)*

Isolate	ST	STATE	Vitek 2 Pen MIC mg/L	*blaZ*	*agr* type	Capsule type	*spa*	Enterotoxins	Other Virulence Genes	IEC (Type)
								(seg+ sei+sem+sen+seo+selw), selx		
ISTOP-357	ST5	WA	0.06	-	II	5	t10218	*sed+selj+ser, egc-cluster (seg+ sei+sem+sen+seo+selw), selx*	-	*sak, chp, scn* (B)
ISTOP-365	ST5	NSW	0.12	-	II	5	t17484	*egc-cluster (seg+ sei+sem+sen+seo+selw), selx*	-	*sak, chp, scn* (B)
ISTOP-369	ST5	NSW	0.12	-	II	5	t088	*egc-cluster (seg+ sei+sem+sen+seo+selw), selx*	-	*sak, chp, scn* (B)
ISTOP-372	ST5	QLD	0.06	-	II	5	t7186	*egc-cluster (seg+ sei+sem+sen+seo+selw), selx*	-	*sak, chp, scn* (B)
ISTOP-374	ST5	VIC	0.06	-	II	5	t002	*egc-cluster (seg+ sei+sem+sen+seo+selw), selx*	-	*sak, scn* (E)
ISTOP-378	ST5	ACT	0.12	-	II	5	t494	*egc-cluster (seg+ sei+sem+sen+seo+selw), selx*	-	*sak, chp, scn* (B)
ISTOP-380	ST5	ACT	0.06	-	II	5	t179	*egc-cluster (seg+ sei+sem+sen+seo+selw), selx*	-	*sak, chp, scn* (B)
ISTOP-381	ST5	ACT	0.06	-	II	5	t002	*sep, egc-cluster (seg+ sei+sem+sen+seo+selw), selx*	-	*sep, sak, scn* (G)
ISTOP-382	ST5	VIC	≤0.03	-	II	5	t002	*sep, egc-cluster (seg+ sei+sem+sen+seo+selw), selx*	-	*sep, sak, chp, scn (F)*
ISTOP-388	ST5	VIC	0.06	-	II	5	t045	*egc-cluster (seg+ sei+sem+sen+seo+selw), selx*	-	*sak, chp, scn* (B)
ISTOP-389	ST5	VIC	0.06	-	II	5	t002	*sep, egc-cluster (seg+ sei+sem+sen+seo+selw), selx*	-	*sep, sak, chp, scn (F)*
ISTOP-390	ST5	VIC	0.06	-	II	5	t105	*egc-cluster (seg+ sei+sem+sen+seo+selw), selx*	-	*sak, chp, scn* (B)
ISTOP-391	ST5	VIC	0.06	-	II	5	t062	*sep, egc-cluster (seg+ sei+sem+sen+seo+selw), selx*	-	*sep, sak, chp, scn (F)*
ISTOP-394	ST5	WA	≤0.03	-	II	5	t242	*sep, egc-cluster (seg+ sei+sem+sen+seo+selw), selx*	*tst*	*sep, sak, chp, scn (F)*

Isolate	ST	STATE	Vitek 2 Pen MIC mg/L	***blaZ***	***agr*** **type**	Capsule type	***spa***	Enterotoxins	Other Virulence Genes	IEC (Type)
ISTOP-395	ST5	WA	0.06	-	II	5	t002	*egc-cluster (seg+ sei+sem+sen+seo+selw), selx*	-	*sak, chp, scn* (B)
ISTOP-398	ST5	WA	0.06	-	II	5	t1265	*sep, egc-cluster (seg+ sei+sem+sen+seo+selw), selx*	-	*sep, sak, scn* (G)
ISTOP-399	ST5	WA	0.12	-	II	5	t1265	*egc-cluster (seg+ sei+sem+sen+seo+selw), selx*	-	*sak, chp, scn* (B)
ISTOP-400	ST5	WA	0.12	-	II	5	t6071	*sea, egc-cluster (seg+ sei+sem+sen+seo+selw), selx*	-	*sea, sak, chp, scn (A)*
ISTOP-410	ST5	NSW	0.06	-	II	5	t1265	*egc-cluster (seg+ sei+sem+sen+seo+selw), selx*	-	*sak, chp, scn* (B)
ISTOP-412	ST5	NSW	0.06	-	II	5	t2595	*egc-cluster (seg+ sei+sem+sen+seo+selw), selx*	-	-
ISTOP-413	ST5	NSW	0.06	-	II	5	t1265	*egc-cluster (seg+ sei+sem+sen+seo+selw), selx*	-	*sak, chp, scn* (B)
ISTOP-415	ST5	NSW	0.06	-	II	5	t179	*egc-cluster (seg+ sei+sem+sen+seo+selw), selx*	-	*sak, chp, scn* (B)
ISTOP-421	ST5	NSW	0.06	-	II	5	t002	*egc-cluster (seg+ sei+sem+sen+seo+selw), selx*	-	*sak, chp, scn* (B)
ISTOP-423	ST5	NSW	0.12	-	II	5	t5349	*egc-cluster (seg+ sei+sem+sen+seo+selw), selx*	-	*sak, chp, scn* (B)
ISTOP-424	ST5	NSW	0.06	-	II	5	t002	*sep, egc-cluster (seg+ sei+sem+sen+seo+selw), selx*	-	*sep, sak, scn* (G)
ISTOP-425	ST5	NSW	0.12	-	II	5	t1265	*egc-cluster (seg+ sei+sem+sen+seo+selw), selx*	-	*sak, chp, scn* (B)
ISTOP-430	ST5	TAS	0.06	-	II	5	t1265	*egc-cluster (seg+ sei+sem+sen+seo+selw), selx*	-	*sak, chp, scn* (B)
ISTOP-431	ST5	TAS	≤0.03	-	II	5	t548	*egc-cluster (seg+ sei+sem+sen+seo+selw), selx*	-	-
ISTOP-432	ST5	TAS	0.06	-	II	5	t6181	*egc-cluster (seg+ sei+sem+sen+seo+selw),*	-	*sak, chp, scn* (B)

Isolate	ST	STATE	Vitek 2 Pen MIC mg/L	*blaZ*	*agr* type	Capsule type	*spa*	Enterotoxins	Other Virulence Genes	IEC (Type)
								selx		
ISTOP-436	ST5	NSW	0.12	-	II	5	t002	*sed+selj+ser, sep, egc-cluster (seg+ sei+sem+sen+seo+selw), selx*	-	*sep, sak, chp, scn (F)*
ISTOP-450	ST5	NSW	≤0.03	-	II	5	t002	*egc-cluster (seg+ sei+sem+sen+seo+selw), selx*	-	-
ISTOP-451	ST5	NSW	0.12	-	II	5	t002	*egc-cluster (seg+ sei+sem+sen+seo+selw), selx*	-	-
ISTOP-452	ST5	NSW	0.06	**DETECTED**	II	5	Unknown	*sec2+sel, sep, egc-cluster (seg+ sei+sem+sen+seo+selw), selx, sel31, sel32*	-	*sep, sak, chp, scn (F)*
ISTOP-459	ST5	QLD	0.12	**DETECTED**	II	5	t579	*sed+selj+ser, sep, egc-cluster (seg+sen+seo+sev+selw), selx*	-	*sep, sak, chp, scn (F)*
ISTOP-461	ST5	QLD	0.06	-	II	5	t1531	*egc-cluster (seg+ sei+sem+sen+seo+selw), selx*	-	*sak, scn (E)*
ISTOP-476	ST5	QLD	≤0.03	-	II	5	t5150	*sep, egc-cluster (seg+ sei+sem+sen+seo+selw), selx*	-	*sep, sak, scn (G)*
ISTOP-478	ST5	QLD	0.06	-	II	5	t045	*egc-cluster (seg+ sei+sem+sen+seo+selw), selx*	-	*sak, chp, scn (B)*
ISTOP-480	ST5	QLD	0.06	-	II	5	t1265	*egc-cluster (seg+ sei+sem+sen+seo+selw), selx*	-	*sak, chp, scn (B)*
ISTOP-481	ST5	QLD	0.12	-	II	5	t2666	*sep, egc-cluster (seg+ sei+sem+sen+seo+selw), selx*	-	*sep, sak, chp, scn (F)*
ISTOP-4	ST2967	WA	≤0.03	-	II	5	t1345	*egc-cluster (seg+ sei+sem+sen+seo+selw), selx*	-	*sak, chp, scn (B)*
ISTOP-13	ST3628	WA	0.06	-	II	5	t5259	*sed+selj+ser, egc-cluster (seg+ sei+sem+sen+seo+selw), selx*	-	*sak, chp, scn (B)*
ISTOP-34	ST3628	NSW	≤0.03	-	II	5	t1265	*egc-cluster (seg+ sei+sem+sen+seo+selw), selx*	-	*sak, chp, scn (B)*
ISTOP-39	ST3628	TAS	0.12	-	II	5	t7026	*egc-cluster (seg+ sei+sem+sen+seo+selw),*	-	*sak, chp, scn (B)*

Isolate	ST	STATE	Vitek 2 Pen MIC mg/L	*blaZ*	*agr* type	Capsule type	*spa*	Enterotoxins	Other Virulence Genes	IEC (Type)
								selx		
ISTOP-121	ST3628	WA	0.12	**DETECTED**	II	5	t179	*sed+selj+ser, egc-cluster (seg+ sei+sem+sen+seo+selw), selx*	-	*sak, chp, scn* (B)
ISTOP-338	ST3628	WA	0.06	-	II	5	t1265	*egc-cluster (seg+ sei+sem+sen+seo+selw), selx*	-	*sak, chp, scn* (B)
ISTOP-349	ST3628	WA	0.12	-	II	5	t7026	*egc-cluster (seg+ sei+sem+sen+seo+selw), selx*	-	*sak, chp, scn* (B)
ISTOP-104	ST3724	SA	0.06	-	II	5	t688	*sea, egc-cluster (seg+ sei+sem+sen+seo+selw), selx*		*sea, sak, scn* (D)
ISTOP-62	ST5189	VIC	0.06	-	II	5	t1265	*sed+selj+ser, egc-cluster (seg+ sei+sem+sen+seo+selw), selx*	-	*sak, chp, scn* (B)
ISTOP-228	ST5189	WA	0.06	-	II	5	t1265	*egc-cluster (seg+ sei+sem+sen+seo+selw), selx*	-	*sak, scn* (E)
ISTOP-41	ST7252	VIC	0.12	-	II	5	t686	*sep, egc-cluster (seg+ sei+sem+sen+seo+selw), selx*	-	*sep, sak, chp, scn (F)*
ISTOP-61	ST7252	VIC	0.06	-	II	5	t686	*sep, egc-cluster (seg+ sei+sem+sen+seo+selw), selx*	-	*sep, sak, chp, scn (F)*
ISTOP-195	ST7260	SA	≤0.03	-	II	5	t002	*egc-cluster (seg+ sei+sem+sen+seo+selw), selx*	-	*sak, chp, scn* (B)
ISTOP-229	ST7262	WA	0.12	**DETECTED**	II	5	t002	*sec2+sel, sep, egc-cluster (seg+ sei+sem+sen+seo+selw), selx*	-	*sep, sak, chp, scn (F)*
ISTOP-238	ST7263	WA	0.12	-	II	5	t045	*sep, egc-cluster (seg+ sei+sem+sen+seo+selw), selx*	-	*sep, sak, chp, scn (F)*
ISTOP-246	ST7265	WA	≤0.03	-	II	5	t002	*egc-cluster (seg+ sei+sem+sen+seo+selw), selx*	-	*sak, scn* (E)
ISTOP-251	ST7267	WA	0.12	-	II	5	t002	*egc-cluster (seg+ sei+sem+sen+seo+selw), selx*	-	*sak, chp, scn* (B)
ISTOP-482	ST7267	QLD	0.06	-	II	5	t570	*egc-cluster (seg+ sei+sem+sen+seo+selw), selx*	-	*sak, chp, scn* (B)

Isolate	ST	STATE	Vitek 2 Pen MIC mg/L	*blaZ*	*agr* type	Capsule type	*spa*	Enterotoxins	Other Virulence Genes	IEC (Type)
ISTOP-271	ST7269	NSW	0.06	-	II	5	t002	*egc-cluster (seg+ sei+sem+sen+seo+selw), selx*	-	*sak, chp, scn* (B)
ISTOP-370	ST7282	NSW	0.06	-	II	5	t1265	*egc-cluster (seg+ sei+sem+sen+seo+selw), selx*	-	*sak, scn* (E)
ISTOP-457	ST7288	NSW	0.06	-	II	5	t688	*egc-cluster (seg+ sei+sem+sen+seo+selw), selx*	-	*sak, chp, scn* (B)
ISTOP-466	ST7290	QLD	0.12	-	II	5	t002	*sea, egc-cluster (seg+ sei+sem+sen+seo+selw), selx*	-	*sea, sak, scn (D)*
**Clonal Complex 6 (n-16)**										
ISTOP-1	ST6	NSW	0.06	-	I	8	t304	*sea, selx*	-	*sea, sak, scn (D)*
ISTOP-53	ST6	NSW	0.12	**DETECTED**	I	8	t701	*sea, seb, selx*	-	*sea, sak, scn (D)*
ISTOP-87	ST6	TAS	≤0.03	-	I	8	t701	*sea, selx*	-	*sea, sak, scn (D)*
ISTOP-91	ST6	SA	0.12	-	I	8	t304	*sea, selx*	-	*sea, sak, scn (D)*
ISTOP-105	ST6	NSW	0.12	-	I	8	t701	*sea, selx*	-	*sea, sak, scn (D)*
ISTOP-114	ST6	NSW	0.12	-	I	8	t701	*selx*	-	*sak, scn (E)*
ISTOP-204	ST6	SA	0.12	-	I	8	t701	*sea, selx*	-	*sea, sak, scn (D)*
ISTOP-244	ST6	SA	0.06	-	I	8	t701	*sea, selx*	-	*sea, sak, scn (D)*
ISTOP-248	ST6	WA	0.12	-	I	8	t304	*sea, selx*	-	*sea, sak, scn (D)*
ISTOP-257	ST6	WA	0.06	-	I	8	t304	*sea, selx*	-	*sea, sak, scn (D)*
ISTOP-285	ST6	NSW	0.12	-	I	8	t4407	*sea, selx*	-	*sea, sak, scn (D)*
ISTOP-375	ST6	VIC	0.06	-	I	8	t304	*sea, selx*	-	*sea, sak, scn (D)*
ISTOP-402	ST6	VIC	0.06	-	I	8	t701	*sea, selx*	-	*sea, sak, scn (D)*
ISTOP-428	ST6	VIC	≤0.03	-	I	8	t701	*sea, seb, selx*	-	*sea, sak, scn (D)*
ISTOP-429	ST6	VIC	0.06	-	I	8	t4298	*sea, selx*	-	*sea, sak, scn (D)*
ISTOP-479	ST6	QLD	0.06	-	I	8	t701	*sea, seb, selx*	-	*sea, sak, scn (D)*
**Clonal Complex 7 (n=3)**										
ISTOP-125	ST7	SA	0.12	-	I	8	t7234	*selx*	-	*sak, chp, scn* (B)
ISTOP-220	ST7	TAS	0.06	-	I	8	t7234	*selx*	-	*sak, chp, scn* (B)
ISTOP-345	ST7	VIC	0.06	-	I	8	t091	*sep, selx*		*sep, sak, scn (G)*

Isolate	ST	STATE	Vitek 2 Pen MIC mg/L	*blaZ*	*agr* type	Capsule type	*spa*	Enterotoxins	Other Virulence Genes	IEC (Type)
**Clonal Complex 8 (n=14)**										
ISTOP-17	ST8	VIC	0.12	-	I	5	t024	*sea, sec2+ sel, sek+selq, selx*	-	*sea, sak, scn (D)*
ISTOP-78	ST8	ACT	0.06	-	I	5	t008	*sed+selj+ser, selx*	-	*sak, scn* (E)
ISTOP-143	ST8	SA	≤0.03	-	I	5	t024	*selx*	-	*sak, chp, scn* (B)
ISTOP-144	ST8	ACT	0.06	-	I	5	Unknown	*selx*	-	*sak, chp, scn* (B)
ISTOP-164	ST8	NSW	0.06	**DETECTED**	I	5	t008	*sek+seq, selx*	-	*sak, chp, scn* (B)
ISTOP-181	ST8	VIC	0.06	-	I	5	t008	*selx*	-	*sak, scn* (E)
ISTOP-215	ST8	VIC	0.12	-	I	5	t10888	*selx*	-	*sak, chp, scn* (B)
ISTOP-239	ST8	SA	0.06	-	I	5	Unknown	*selx*	-	*sak, scn* (E)
ISTOP-259	ST8	ACT	≤0.03	-	I	5	t008	*sek+seq, selx*	-	*sak, chp, scn* (B)
ISTOP-286	ST8	NSW	0.06	-	I	5	t008	*sea, selx*	-	*sea, sak, scn (D)*
ISTOP-331	ST8	NSW	0.06	-	I	5	t1171	*selx*	-	*sak, scn* (E)
ISTOP-397	ST8	WA	0.06	-	I	5	t008	*selx*	-	*sak, chp, scn* (B)
ISTOP-405	ST8	VIC	0.12	-	I	5	t008	*selx*	-	*sak, chp, scn* (B)
ISTOP-6	ST5234	WA	0.06	-	I	5	t955	*seb, sek+seq, selx*	-	*sak, scn* (E)
**Clonal Complex 9 (n=1)**										
ISTOP-293	ST9	VIC	0.12	**DETECTED**	II	5	t4812	*egc-cluster (seg+ sei+sem+sen+seo+selw), selx, sely, sel27, sel28*	*etA*	*sak, chp, scn* (B)
**Clonal Complex 12 (n=16)**										
ISTOP-12	ST12	WA	≤0.03	-	II	8	Unknown	*seb, selx, selz*	-	*sak, chp, scn* (B)
ISTOP-14	ST12	WA	0.06	-	II	8	t160	*sep, selx, selz*	-	*sep, sak, scn* (G)
ISTOP-29	ST12	QLD	≤0.03	-	II	8	t336	*sep, selx, selz*	-	*sep, sak, scn* (G)
ISTOP-30	ST12	QLD	0.06	-	II	8	Unknown	*seb, selx, selz*	-	*sak, chp, scn* (B)
ISTOP-40	ST12	TAS	0.12	-	II	8	t909	*sec2+sel, sep, selx, selz*	-	*sep, sak, scn* (G)
ISTOP-51	ST12	NSW	0.06	-	II	8	Unknown	*seb, selx, selz*	-	*sak, chp, scn* (B)
ISTOP-74	ST12	NSW	0.06	-	II	8	t771	*seb, selx, selz*	-	*sak, chp, scn* (B)
ISTOP-86	ST12	TAS	0.06	-	II	8	t771	*seb, selx, selz*	-	*sak, chp, scn* (B)
ISTOP-117	ST12	WA	0.06	-	II	8	t160	*seb, sep, selx, selz*	-	*sep, sak, scn* (G)
ISTOP-140	ST12	VIC	0.12	-	II	8	t160	*sep, selx, selz*	-	*sep, sak, scn* (G)

Isolate	ST	STATE	Vitek 2 Pen MIC mg/L	*blaZ*	*agr* type	Capsule type	*spa*	Enterotoxins	Other Virulence Genes	IEC (Type)
ISTOP-150	ST12	ACT	0.06	-	II	8	t160	*seb, sep, selx, selz*	-	*sep, sak, scn* (G)
ISTOP-254	ST12	WA	0.06	-	II	8	t160	*sep, selx, selz*	-	*sep, sak, scn* (G)
ISTOP-274	ST12	TAS	0.06	-	II	8	t160	*seb, sep, selx, selz*	-	*sep, sak, scn* (G)
ISTOP-377	ST12	NSW	0.06	-	II	8	t771	*seb, selx, selz*	-	*sak, chp, scn* (B)
ISTOP-406	ST12	VIC	0.06	-	II	8	t160	*seb, sep, selx, selz*	-	*sep, sak, scn* (G)
ISTOP-23	ST7251	VIC	0.06	**DETECTED**	II	8	t160	*seb, sep, selx, selz*	-	*sep, sak, scn* (G)
**Clonal Complex 15 (n=41)**										
ISTOP-7	ST15	WA	0.06	-	II	8	t605	*selx*	-	*chp, scn* (C)
ISTOP-46	ST15	NSW	0.12	-	II	8	t803	*selx*	-	*chp, scn* (C)
ISTOP-59	ST15	NSW	0.06	-	II	8	t085	*selx*	-	*chp, scn* (C)
ISTOP-79	ST15	ACT	0.06	-	II	8	t084	*selx*	-	*chp, scn* (C)
ISTOP-109	ST15	QLD	0.12	-	II	8	t803	*selx*	-	*chp, scn* (C)
ISTOP-119	ST15	WA	0.12	-	II	8	t084	*selx*	-	*chp, scn* (C)
ISTOP-129	ST15	NSW	0.06	-	II	8	t2216	*selx*	-	*chp, scn* (C)
ISTOP-134	ST15	NSW	0.12	-	II	8	t14014	*selx*	-	*chp, scn* (C)
ISTOP-137	ST15	VIC	0.06	-	II	8	t877	*selx*	-	*chp, scn* (C)
ISTOP-221	ST15	TAS	≤0.03	-	II	8	t346	*selx*	-	*chp, scn* (C)
ISTOP-225	ST15	WA	0.12	-	II	8	t084	*selx*	-	*chp, scn* (C)
ISTOP-235	ST15	WA	0.06	-	II	8	t346		-	*chp, scn* (C)
ISTOP-236	ST15	WA	0.12	-	II	8	t085	*selx*	-	*chp, scn* (C)
ISTOP-299	ST15	VIC	0.12	-	II	8	t084	*seb, selx*	-	*chp, scn* (C)
ISTOP-309	ST15	VIC	0.12	-	II	8	t084	*seb, selx*	-	*chp, scn* (C)
ISTOP-348	ST15	WA	0.06	-	II	8	t491	*selx*	-	*chp, scn* (C)
ISTOP-356	ST15	WA	0.06	-	II	8	t605	*selx*	-	*chp, scn* (C)
ISTOP-379	ST15	ACT	0.06	-	II	8	t5393	*selx*	-	*chp, scn* (C)
ISTOP-438	ST15	NSW	0.12	**DETECTED**	II	8	t11928	*selx*	-	*chp, scn* (C)
ISTOP-20	ST333	VIC	0.06	-	II	8	Unknown	*selx*	-	*chp, scn* (C)
ISTOP-50	ST582	NSW	0.12	**DETECTED**	II	8	t085	*selx*	-	*chp, scn* (C)
ISTOP-153	ST582	TAS	0.12	**DETECTED**	II	8	t084	*selx*	-	*chp, scn* (C)

Isolate	ST	STATE	Vitek 2 Pen MIC mg/L	*blaZ*	*agr* type	Capsule type	*spa*	Enterotoxins	Other Virulence Genes	IEC (Type)
ISTOP-157	ST582	NSW	0.12	**DETECTED**	II	8	t393	*selx*	-	*chp, scn* (C)
ISTOP-223	ST582	WA	0.06	**DETECTED**	II	8	t085	*selx*	*etA*	*chp, scn* (C)
ISTOP-306	ST582	Qld	0.12	**DETECTED**	II	8	t084	*selx*	-	*chp, scn* (C)
ISTOP-318	ST582	VIC	0.12	**DETECTED**	II	8	t084	*selx*	-	*chp, scn* (C)
ISTOP-324	ST582	VIC	0.06	**DETECTED**	II	8	t084	*selx*	-	*chp, scn* (C)
ISTOP-447	ST582	NSW	0.06	**DETECTED**	II	8	t1509	*selx*	-	*chp, scn* (C)
ISTOP-462	ST582	QLD	0.12	**DETECTED**	II	8	t393	*selx*	-	*chp, scn* (C)
ISTOP-465	ST582	QLD	0.12	**DETECTED**	II	8	t393	*selx*	*etA*	*chp, scn* (C)
ISTOP-475	ST582	QLD	0.12	**DETECTED**	II	8	t084	*selx*	-	*chp, scn* (C)
ISTOP-27	ST3911	VIC	0.06	**DETECTED**	II	8	t2859	*selx*	-	*chp, scn* (C)
ISTOP-171	ST3911	WA	0.12	**DETECTED**	II	8	t084	*selx*	-	*chp, scn* (C)
ISTOP-210	ST3911	QLD	0.06	**DETECTED**	II	8	t084	*selx*	-	*chp, scn* (C)
ISTOP-276	ST3911	NSW	0.12	**DETECTED**	II	8	t279	*selx*	-	*chp, scn* (C)
ISTOP-295	ST3911	VIC	0.12	**DETECTED**	II	8	t084	*selx*	-	*chp, scn* (C)
ISTOP-115	ST5059	NSW	0.12	**DETECTED**	II	8	t4714	*selx*	-	*chp, scn* (C)
ISTOP-243	ST7264	SA	0.06	-	II	8	t085	*selx*	-	*chp, scn* (C)
ISTOP-300	ST7273	QLD	0.12	**DETECTED**	II	8	t084	*selx*	-	*chp, scn* (C)
ISTOP-401	ST7283	VIC	0.12	**DETECTED**	II	8	t547	*selx*	-	*chp, scn* (C)
ISTOP-445	ST7286	NSW	≤0.03	-	II	8	t144	*selx*	-	*chp, scn* (C)
**Clonal Complex 20 (n=3)**										
ISTOP-85	ST20	TAS	0.12	-	I	5	t3277	*egc-cluster (seg+ sei+sem+sen+seo+selw), selx, sely*	-	*sak, chp, scn (*B)
ISTOP-342	ST20	TAS	0.12	-	I	5	t2919	*egc-cluster (seg+ sei+sem+sen+seo+selw), selx, sely*	-	-
ISTOP-427	ST20	SA	0.12	**DETECTED**	I	5	t164	*seb, egc-cluster (seg+ sei+sem+sen+seo+selw), selx, sely*	-	*sak, chp, scn (*B)
**Clonal Complex 22 (n=8)**										
ISTOP-266	ST22	ACT	0.06	-	I	5	t474	*egc-cluster (seg+ sei+sem+sen+seo+selw), selx*	-	*sak, chp, scn (*B)

Isolate	ST	STATE	Vitek 2 Pen MIC mg/L	*blaZ*	*agr* type	Capsule type	*spa*	Enterotoxins	Other Virulence Genes	IEC (Type)
ISTOP-278	ST22	NSW	0.12	-	I	5	t852	*egc-cluster (seg+ sei+sem+sen+seo+selw), selx*	-	*sak, chp, scn* (B)
ISTOP-347	ST22	WA	0.12	-	I	5	t3243	*egc-cluster (seg+ sei+sem+sen+seo+selw), selx*	*tst*	*sak, chp, scn* (B)
ISTOP-360	ST22	WA	0.06	**DETECTED**	I	5	Unknown	*sec1+sel, egc-cluster (seg+ sei+sem+sen+seo+selw), selx*	-	*sak, chp, scn* (B)
ISTOP-383	ST22	VIC	0.12	-	I	5	t2933	*egc-cluster (seg+ sei+sem+sen+seo+selw), selx*	*tst*	*sak, chp, scn* (B)
ISTOP-384	ST22	VIC	≤0.03	-	I	5	t1328	*egc-cluster (seg+ sei+sem+sen+seo+selw), selx*	-	*sak, chp, scn* (B)
ISTOP-292	ST7272	QLD	≤0.03	-	I	5	t005	*egc-cluster (seg+ sei+sem+sen+seo+selw), selx*	-	*sak, chp, scn* (B)
ISTOP-435	ST7285	TAS	0.06	-	I	5	Unknown	*egc-cluster (seg+ sei+sem+sen+seo+selw), selx*	-	*sak, chp, scn* (B)
**Clonal Complex 25 (n=1)**										
ISTOP-325	ST7276	VIC	≤0.03	**DETECTED**	I	5	-	*egc-cluster (seg+ sei+sem+sen+seo+selw), selx*	*edinB*	*sak, chp, scn* (B)
**Clonal Complex 30 (n=7)**										
ISTOP-311	ST30	VIC	0.06	-	III	8	t3037	*egc-cluster (seg+ sei+sem+sen+seo+selw)*	*lukF/S-PVL*	*sak, chp, scn* (B)
ISTOP-319	ST30	VIC	0.12	**DETECTED**	III	8	t012	*egc-cluster (seg+ sei+sem+sen+seo+selw))*	*tst*	-
ISTOP-42	ST34	VIC	0.06	**DETECTED**	III	8	Unknown	*seh, egc-cluster (seg+ sei+sem+sen+seo+selw)*	*tst*	*sak, chp, scn* (B)
ISTOP-101	ST34	VIC	≤0.03	-	III	5	t1670	*seb, seh, sel, egc-cluster (seg+ sei+sem+sen+seo+selw)*	*tst*	*sak, chp, scn* (B)
ISTOP-263	ST34	ACT	0.06	**DETECTED**	III	8	t089	*seh, egc-cluster (seg+ sei+sem+sen+seo+selw)*	*tst*	*sak, chp, scn* (B)
ISTOP-264	ST34	ACT	0.06	**DETECTED**	III	8	t089	*seh, egc-cluster*	*tst*	*sak, chp, scn* (B)

Isolate	ST	STATE	Vitek 2 Pen MIC mg/L	*blaZ*	*agr* type	Capsule type	*spa*	Enterotoxins	Other Virulence Genes	IEC (Type)
								(seg+ sei+sem+sen+seo+selw)		
ISTOP-296	ST39	VIC	≤0.03	-	III	8	t1504	*sec3+sel, selo*	*tst*	*sak, scn* (E)
**Clonal Complex 45 (n=47)**										
ISTOP-31	ST45	QLD	0.06	-	I	8	t026	*sec2+sel, egc-cluster (seg+ sei+sem+sen+seo+selw), selx*	-	*sak, chp, scn* (B)
ISTOP-68	ST45	WA	0.06	-	I	8	t230	*sec2+sel, egc-cluster (seg+ sei+sem+sen+seo+selw), selx*	-	*sak, chp, scn* (B)
ISTOP-70	ST45	NSW	0.06	-	I	8	t563	*seb, egc-cluster (seg+ sei+sem+sen+seo+selw), selx*	-	*sak, chp, scn* (B)
ISTOP-88	ST45	TAS	0.12	**DETECTED**	I	8	t015	*egc-cluster (seg+ sei+sem+sen+seo+selw), selx*	-	*sak, chp, scn* (B)
ISTOP-94	ST45	SA	0.12	-	I	8	t371	*egc-cluster (seg+ sei+sem+sen+seo+selw), selx*	-	*sak, chp, scn* (B)
ISTOP-122	ST45	WA	0.06	-	I	8	t130	*egc-cluster (seg+ sei+sem+sen+seo+selw), selx*	-	*chp, scn* (C)
ISTOP-123	ST45	SA	0.12	-	I	8	t302	*sec2+sel, selx*	-	*sak, chp, scn* (B)
ISTOP-139	ST45	VIC	≤0.03	-	I	8	t10588	*sec2+sel, egc-cluster (seg+ sei+sem+sen+seo+selw), selx*	-	*sak, chp, scn* (B)
ISTOP-149	ST45	ACT	0.06	-	I	8	t015	*sec2+sel, egc-cluster (seg+ sei+sem+sen+seo+selw), selx*	-	*sak, chp, scn* (B)
ISTOP-160	ST45	NSW	0.06	-	I	8	t728	*sec2+sel, egc-cluster (seg+ sei+sem+sen+seo+selw), selx*	-	*sak, chp, scn* (B)
ISTOP-170	ST45	WA	≤0.03	-	I	8	t8453	*sec2+sel, egc-cluster (seg+ sei+sem+sen+seo+selw), selx*	-	*sak, chp, scn* (B)
ISTOP-189	ST45	NSW	0.06	-	I	8	t10421	*sec2+sel, egc-cluster (seg+ sei+sem+sen+seo+selw), selx*	-	*sak, chp, scn* (B)
ISTOP-216	ST45	TAS	0.06	-	I	8	t362	*egc-cluster (seg+ sei+sem+sen+seo+selw), selx*	-	*sak, chp, scn* (B)
ISTOP-230	ST45	WA	0.06	-	I	8	t230	*sec2+sel, egc-cluster*	-	*sak, chp, scn* (B)

Isolate	ST	STATE	Vitek 2 Pen MIC mg/L	***blaZ***	***agr* type**	Capsule type	***spa***	Enterotoxins	Other Virulence Genes	IEC (Type)
								(seg+ sei+sem+sen+seo+selw), selx		
ISTOP-260	ST45	ACT	0.06	-	I	8	t706	*egc-cluster (seg+ sei+sem+sen+seo+selw), selx*	-	*sak, chp, scn* (B)
ISTOP-279	ST45	NSW	0.12	-	I	8	t015	*egc-cluster (seg+ sei+sem+sen+seo+selw), selx*	-	*sak, chp, scn* (B)
ISTOP-283	ST45	NSW	0.06	-	I	8	t10771	*egc-cluster (seg+ sei+sem+sen+seo+selw), selx*	-	*sak, chp, scn* (B)
ISTOP-337	ST45	WA	0.06	-	I	8	Unknown	*sec3+sel, egc-cluster (seg+ sei+sem+sen+seo+selw), selx*	*tst*	*sak, chp, scn* (B)
ISTOP-366	ST45	NSW	0.06	-	I	8	t362	*egc-cluster (seg+ sei+sem+sen+seo+selw), selx*	-	*sak, chp, scn* (B)
ISTOP-373	ST45	VIC	0.06	-	I	8	t015	*sec2+sel, egc-cluster (seg+ sei+sem+sen+seo+selw), selx*	-	*sak, chp, scn* (B)
ISTOP-376	ST45	WA	0.06	-	I	8	t015	*sec2+sel, egc-cluster (seg+ sei+sem+sen+seo+selw), selx*	-	*sak, scn* (E)
ISTOP-386	ST45	QLD	0.06	-	I	8	t302	*sec2+sel, egc-cluster (seg+ sei+sem+sen+seo+selw), selx*	-	*sak, chp, scn* (B)
ISTOP-426	ST45	SA	0.06	-	I	8	t371	*egc-cluster (seg+ sei+sem+sen+seo+selw), selx*	-	*sak, chp, scn* (B)
ISTOP-439	ST45	NT	0.12	-	I	8	t371	*egc-cluster (seg+ sei+sem+sen+seo+selw), selx*	-	*sak, chp, scn* (B)
ISTOP-454	ST45	NSW	0.06	-	I	8	t1510	*sec2+sel, egc-cluster (seg+ sei+sem+sen+seo+selw), selx*	-	*sak, chp, scn* (B)
ISTOP-455	ST45	NSW	0.06	-	I	8	t1510	*sec2+sel, egc-cluster (seg+ sei+sem+sen+seo+selw), selx*	-	*sak, chp, scn* (B)
ISTOP-456	ST45	NSW	≤0.03	-	I	8	t026	*sec2+sel, egc-cluster (seg+ sei+sem+sen+seo+selw), selx*	*tst*	*sak, chp, scn* (B)
ISTOP-468	ST45	QLD	≤0.03	-	I	8	t589	*sec2+sel, egc-cluster (seg+ sei+sem+sen+seo+selw), selx*	-	*sak, chp, scn* (B)

Isolate	ST	STATE	Vitek 2 Pen MIC mg/L	*blaZ*	*agr* type	Capsule type	*spa*	Enterotoxins	Other Virulence Genes	IEC (Type)
ISTOP-470	ST45	QLD	0.06	-	I	8	t1078	*sec2+sel, egc-cluster (seg+ sei+sem+sen+seo+selw), selx*	-	*sak, chp, scn* (B)
ISTOP-473	ST45	QLD	≤0.03	-	I	8	t026	*sec2+sel, egc-cluster (seg+ sei+sem+sen+seo+selw), selx*	-	*sak, chp, scn* (B)
ISTOP-26	ST508	NSW	≤0.03	-	I	8	Unknown	*sec3+sel, egc-cluster (seg+ sei+sem+sen+seo+selw), selx*	*tst*	*sak, chp, scn* (B)
ISTOP-35	ST508	NSW	0.12	-	I	8	t015	*sec3+sel, egc-cluster (seg+ sei+sem+sen+seo+selw), selx*	*tst*	*sak, chp, scn* (B)
ISTOP-154	ST508	TAS	≤0.03	-	I	8	t073	*egc-cluster (seg+ sei+sem+sen+seo+selw), selx*	-	*sak, chp, scn* (B)
ISTOP-163	ST508	NSW	0.06	-	I	8	t015	*sec3+sel, egc-cluster (seg+ sei+sem+sen+seo+selw), selx*	*tst*	*sak, chp, scn* (B)
ISTOP-169	ST508	WA	0.06	-	I	8	t015	*sec3+sel, egc-cluster (seg+ sei+sem+sen+seo+selw), selx*	*tst*	*sak, chp, scn* (B)
ISTOP-253	ST508	WA	0.12	-	I	8	t015	*sec3+sel, egc-cluster (seg+ sei+sem+sen+seo+selw), selx*	*tst*	*sak, chp, scn* (B)
ISTOP-269	ST508	NSW	0.125	-	I	8	t026	*egc-cluster (seg+ sei+sem+sen+seo+selw), selx*	-	*sak, chp, scn* (B)
ISTOP-336	ST508	SA	0.125	-	I	8	t050	*egc-cluster (seg+ sei+sem+sen+seo+selw), selx*	-	*sak, chp, scn* (B)
ISTOP-408	ST508	VIC	0.06	-	I	8	t015	*sec3+sel, egc-cluster (seg+ sei+sem+sen+seo+selw), selx*	*tst*	*sak, chp, scn* (B)
ISTOP-75	ST7254	NSW	≤0.03	-	I	8	t132	*egc-cluster (seg+ sei+sem+sen+seo+selw), selx*	-	*sak, chp, scn* (B)
ISTOP-84	ST7255	TAS	0.12	**DETECTED**	I	8	t230	*sec2+sel, egc-cluster (seg+ sei+sem+sen+seo+selw), selx*	-	*sak, chp, scn* (B)
ISTOP-147	ST7258	ACT	0.06	-	I	8	Unknown	*egc-cluster (seg+ sei+sem+sen+seo+selw), selx*	-	*sak, chp, scn* (B)
ISTOP-203	ST7261	SA	≤0.03	-	I	8	t2726	*sec3+sel, egc-cluster*	*tst*	*sak, chp, scn* (B)

Isolate	ST	STATE	Vitek 2 Pen MIC mg/L	*blaZ*	*agr* type	Capsule type	*spa*	Enterotoxins	Other Virulence Genes	IEC (Type)
								(seg+ sei+sem+sen+seo+selw), selx		
ISTOP-268	ST7268	NSW	0.06	-	I	8	t015	*egc-cluster (seg+ sei+sem+sen+seo+selw), selx*	-	*sak, chp, scn* (B)
ISTOP-346	ST7279	VIC	0.06	-	I	8	t050	*egc-cluster (seg+ sei+sem+sen+seo+selw), selx*	-	*sak, chp, scn* (B)
ISTOP-419	ST7284	NSW	0.12	-	I	8	t715	*egc-cluster (seg+ sei+sem+sen+seo+selw), selx*	-	*sak, chp, scn* (B)
ISTOP-463	ST7289	QLD	0.12	-	I	8	t026	*sec2+sel, egc-cluster (seg+ sei+sem+sen+seo+selw), selx*	*tst*	*sak, chp, scn* (B)
**Clonal Complex 59 (n=9)**										
ISTOP-67	ST59	WA	0.12	-	I	8	t437	*sea, seb, sek+seq, selx, sely*	-	*sea, sak, scn* (D)
ISTOP-77	ST59	ACT	0.06	-	I	8	t216	*seb, sek+seq, selx, sely*	-	*scn, sak, chp* (B)
ISTOP-343	ST59	TAS	0.06	-	I	8	t471	*selx, sely*	-	*chp, scn* (C)
ISTOP-368	ST59	NSW	0.12	-	I	8	t316	*selx, sely*	-	*sak, chp, scn* (B)
ISTOP-403	ST59	VIC	0.12	-	I	8	t437	*selx, sely*	-	*chp, scn* (C)
ISTOP-437	ST59	NSW	0.06	-	I	8	t1293	*seb, sek+seq, selx, sely*	-	*chp, scn* (C)
ISTOP-256	ST87	WA	0.12	-	I	8	t316	*seb, sek+seq, selx, sely*	-	*sak, chp, scn* (B)
ISTOP-186	ST1224	NSW	≤0.03	-	I	8	t471	*selx, sely*		*chp, scn* (C)
ISTOP-354	ST7280	WA	0.06	-	I	8	t216	*sea, seb, sek+seq, selx, sely*	-	*sea, sak, chp, scn* (A)
**Clonal Complex 80 (n=1)**										
ISTOP-199	ST80	VIC	0.12	-	III	8	t042	*seb, seh, sel+ seq, selx, sely*	*edinB*	*sak, chp, scn* (B)
**Clonal Complex 88 (n=17)**										
ISTOP-47	ST78	NSW	0.12	-	III	8	t1814	*selx*	-	*sak, scn* (E)
ISTOP-76	ST78	ACT	0.12	-	III	8	Unknown	*selx*	-	*sak, scn* (E)
ISTOP-182	ST78	VIC	0.12	-	III	8	t786	*Selx*	-	*sak, scn* (E)
ISTOP-217	ST78	TAS	0.06	-	III	8	t2311	*Selx*	-	*sak, scn* (E)
ISTOP-261	ST78	ACT	0.06	-	III	8	t2177	*sec2+sel, selx*	-	*sak, scn* (E)
ISTOP-312	ST78	NSW	0.06	-	III	8	t2191	*sec2+sel, selx*	-	-
ISTOP-471	ST78	QLD	0.06	-	III	8	t186	*selx*	-	*sak, scn* (E)

Isolate	ST	STATE	Vitek 2 Pen MIC mg/L	*blaZ*	*agr* type	Capsule type	*spa*	Enterotoxins	Other Virulence Genes	IEC (Type)
ISTOP-8	ST88	WA	0.12	-	III	8	Unknown	*selx*	-	*sak, scn* (E)
ISTOP-71	ST88	NSW	0.06	-	III	8	t4013	*selx*	-	*sak, scn* (E)
ISTOP-120	ST88	WA	0.12	-	III	8	Unknown	*selx*	-	*sak, scn* (E)
ISTOP-135	ST88	NSW	0.12	-	III	8	t2649	*selx*	-	*sak, scn* (E)
ISTOP-175	ST88	VIC	0.06	-	III	8	Unknown	*selx*	-	*sak, scn* (E)
ISTOP-308	ST88	VIC	0.12	-	III	8	t3341	*selx*	*lukF/S-PVL*	*sak, scn* (E)
ISTOP-333	ST88	NSW	0.12	-	III	8	t6928	*selx*	-	*sak, scn* (E)
ISTOP-441	ST88	NT	0.12	-	III	8	t4013	*selx*	-	*sak, scn* (E)
ISTOP-444	ST88	NT	≤0.03	-	III	8	Unknown	*selx*	-	*sak, scn* (E)
ISTOP-355	ST7281	WA	≤0.03	-	III	8	t730	*sec2+sel, selx*	-	*sak, scn* (E)
**Clonal Complex 97 (n=48)**										
ISTOP-37	ST97	NSW	0.06	-	I	5	t9432	*selx*	-	*sak, scn* (E)
ISTOP-54	ST97	NSW	0.12	-	I	5	t267	*selx*	-	*sak, scn* (E)
ISTOP-57	ST97	NSW	0.06	-	I	5	t359	*selx*	-	*sak, scn* (E)
ISTOP-65	ST97	NSW	0.12	-	I	5	t14122	*selx*	-	*sak, scn* (E)
ISTOP-73	ST97	NSW	0.12	-	I	5	t267	*selx*	-	*sak, scn* (E)
ISTOP-80	ST97	NSW	0.12	-	I	5	t359	*selx*	-	*sak, scn* (E)
ISTOP-96	ST97	VIC	≤0.03	-	I	5	t267	*selx*	-	*sak, scn* (E)
ISTOP-138	ST97	VIC	0.06	-	I	5	t267	*selx*	-	*sak, scn* (E)
ISTOP-172	ST97	VIC	0.06	-	I	5	t1028	*selx*	-	*sak, scn* (E)
ISTOP-198	ST97	SA	0.12	-	I	5	t1236	*selx*	-	*sak, scn* (E)
ISTOP-211	ST97	QLD	0.06	-	I	5	t231	*selx*	-	*sak, scn* (E)
ISTOP-242	ST97	SA	0.12	-	I	5	t267	*selx*	-	*sak, scn* (E)
ISTOP-245	ST97	WA	0.12	-	I	5	t359	*selx*	-	*sak, scn* (E)
ISTOP-250	ST97	WA	0.12	-	I	5	t3380	*selx*	-	*sak, scn* (E)
ISTOP-280	ST97	NSW	0.12	-	I	5	t7753	*selx*	-	*sak, scn* (E)
ISTOP-291	ST97	QLD	0.06	-	I	5	t267	*selx*	-	*sak, scn* (E)
ISTOP-298	ST97	VIC	0.12	-	I	5	t224	*sec3+sel, selx*	*tst*	*sak, scn* (E)
ISTOP-307	ST97	VIC	≤0.03	-	I	5	t224	*sec3+sel, selx*	*tst*	*sak, scn* (E)

Isolate	ST	STATE	Vitek 2 Pen MIC mg/L	*blaZ*	*agr* type	Capsule type	*spa*	Enterotoxins	Other Virulence Genes	IEC (Type)
ISTOP-320	ST97	VIC	0.12	-	I	5	t2734	*selx*	-	*sak, scn* (E)
ISTOP-335	ST97	SA	≤0.03	-	I	5	t267	*selx*	-	*sak, scn* (E)
ISTOP-352	ST97	WA	0.12	-	I	5	t693	*selx*	-	*sak, scn* (E)
ISTOP-358	ST97	WA	0.06	-	I	5	t10212	*selx*	-	*sak, scn* (E)
ISTOP-359	ST97	WA	≤0.03	-	I	5	Unknown	*selx*	-	*sak, scn* (E)
ISTOP-367	ST97	NSW	0.12	-	I	5	t267	*selx*	-	*sak, scn* (E)
ISTOP-385	ST97	QLD	0.06	-	I	5	t267	*selx*	-	*sak, scn* (E)
ISTOP-414	ST97	NSW	0.06	-	I	5	t359	*selx*	-	*sak, scn* (E)
ISTOP-416	ST97	NSW	0.12	-	I	5	t1028	*selx*	-	*sak, scn* (E)
ISTOP-420	ST97	NSW	0.12	-	I	5	t267	*selx*	-	*sak, scn* (E)
ISTOP-422	ST97	NSW	0.12	-	I	5	t267	*selx*	-	*sak, scn* (E)
ISTOP-434	ST97	TAS	0.12	-	I	5	t231	*selx*	-	*sak, scn* (E)
ISTOP-449	ST97	NSW	0.12	-	I	5	t267	*selx*	-	*sak, scn* (E)
ISTOP-469	ST97	QLD	0.06	-	I	5	t267	*selx*	-	*sak, scn* (E)
ISTOP-474	ST97	QLD	0.06	-	I	5	t267	*selx*	-	*sak, scn* (E)
ISTOP-477	ST97	QLD	0.06	-	I	5	Unknown	*selx*	-	*sak, scn* (E)
ISTOP-10	ST953	WA	0.12	-	I	5	t267	*selx*	-	*sak, scn* (E)
ISTOP-45	ST953	NSW	0.06	-	1	5	t359	*selx*	-	*sak, scn* (E)
ISTOP-58	ST953	NSW	0.12	-	1	5	t1109	*selx*	-	*sak, scn* (E)
ISTOP-126	ST953	SA	0.12	-	1	5	t267	*selx*	-	*sak, scn* (E)
ISTOP-234	ST953	WA	≤0.03	-	1	5	t267	*selx*	-	*sak, scn* (E)
ISTOP-237	ST953	WA	0.12	-	1	5	t2802	*selx*	-	*sak, scn* (E)
ISTOP-255	ST953	NSW	0.06	-	1	5	t359	*selx*	-	*sak, scn* (E)
ISTOP-273	ST953	TAS	0.12	-	1	5	t267	*selx*	-	*sak, scn* (E)
ISTOP-392	ST953	NSW	≤0.03	-	1	5	t267	*selx*	-	*sak, scn* (E)
ISTOP-443	ST953	NT	≤0.03	-	1	5	t267	*selx*	-	*sak, scn* (E)
ISTOP-5	ST1179	WA	0.12	-	1	5	t237	*selx*	-	*sak, scn* (E)
ISTOP-404	ST1179	VIC	0.12	-	1	5	t267	*selx*	-	*sak, scn* (E)
ISTOP-99	ST7256	VIC	0.06	-	1	5	t359	*selx*	-	*sak, scn* (E)

**Isolate**	**ST**	**STATE**	**Vitek 2 Pen MIC mg/L**	***blaZ***	***agr* type**	**Capsule type**	***spa***	**Enterotoxins**	**Other Virulence Genes**	**IEC (Type)**
ISTOP-344	ST7278	TAS	0.12	-	1	5	t359	*selx*	-	*sak, scn* (E)
**Clonal Complex 101 (n=16)**										
ISTOP-136	ST101	TAS	0.06	-	I	8	t528	*selx*	-	*sak, scn* (E)
ISTOP-174	ST101	VIC	0.12	**DETECTED**	I	8	t2078	*selx*	-	*sak, scn* (E)
ISTOP-192	ST101	VIC	0.12	-	I	8	t643	*selx*	-	*sak, scn* (E)
ISTOP-284	ST101	NSW	0.12	-	I	8	t528	*selx*	-	*sak, scn* (E)
ISTOP-289	ST101	NSW	0.06	-	I	8	t528	*selx*	-	*sak, scn* (E)
ISTOP-294	ST101	VIC	0.06	-	I	8	t528	*selx*	-	*sak, scn* (E)
ISTOP-332	ST101	NSW	0.06	-	I	8	t528	*selx*	-	*sak, scn* (E)
ISTOP-340	ST101	NSW	0.12	-	I	8	t528	*selx*	-	*sak, scn* (E)
ISTOP-361	ST101	NSW	0.12	-	I	8	t528	*selx*	-	*sak, scn* (E)
ISTOP-393	ST101	NSW	≤0.03	-	I	8	t528	*selx*	-	*sak, scn* (E)
ISTOP-411	ST101	NSW	0.06	-	I	8	t2078	*selx*	-	*sak, scn* (E)
ISTOP-484	ST101	QLD	0.12	-	I	8	t528	*selx*	-	*sak, scn* (E)
ISTOP-72	ST1155	QLD	0.12	-	I	8	t4171	*sec2+sel, selx*	-	*sak, scn* (E)
ISTOP-168	ST1155	WA	0.06	-	I	8	Unknown	*sec2+sel, selx*	-	*sak, scn* (E)
ISTOP-133	ST7257	NSW	0.06	-	I	8	Unknown	*selx*	-	*sak, scn* (E)
ISTOP-305	ST7274	QLD	0.06	-	I	8	t528	*selx*	-	*sak, scn* (E)
**Clonal Complex 188 (n=23)**										
ISTOP-2	ST188	NSW	0.06	-	I	8	t189	*selx*	-	*sak, scn (E)*
ISTOP-24	ST188	VIC	0.12	-	I	8	t189	*sep, selx*	-	*sep, sak, scn* (G)
ISTOP-25	ST188	NSW	≤0.03	-	I	8	t189	*selx*	-	*sak, chp, scn* (B)
ISTOP-44	ST188	NSW	0.12	-	I	8	t189	*selx*	-	*sak, chp, scn* (B)
ISTOP-52	ST188	NSW	0.12	-	I	8	t2883	*selx*	-	*sak, chp, scn* (B)
ISTOP-56	ST188	NSW	0.06	-	I	8	t189	*selx*	-	*sak, scn* (E)
ISTOP-92	ST188	SA	0.06	-	I	8	t189	*selx*	-	*sak, scn (E)*
ISTOP-112	ST188	NSW	0.06	-	I	8	t189	*selx*	-	*sak, scn (E)*
ISTOP-155	ST188	TAS	0.12	-	I	8	t189	*selx*	-	*sak, scn (E)*
ISTOP-156	ST188	NSW	0.06	-	I	8	t189	*selx*	-	*sak, chp, scn* (B)

Isolate	ST	STATE	Vitek 2 Pen MIC mg/L	*blaZ*	*agr* type	Capsule type	*spa*	Enterotoxins	Other Virulence Genes	IEC (Type)
ISTOP-165	ST188	NSW	0.12	-	I	8	t189	*selx*	-	*sak, chp, scn* (B)
ISTOP-205	ST188	NSW	0.12	-	I	8	t189	*selx*	-	*sak, chp, scn* (B)
ISTOP-317	ST188	VIC	0.12	-	I	8	t189	*selx*	-	*sak, scn* (E)
ISTOP-417	ST188	NSW	≤0.03	-	I	8	t189	*selx*	-	*sak, chp, scn* (B)
ISTOP-440	ST188	NT	0.12	-	I	8	t189	*seb, selx*	-	*sak, scn* (E)
ISTOP-446	ST188	NSW	0.06	-	I	8	Unknown	*sep, selx*	-	*sep, sak, scn* (G)
ISTOP-458	ST188	QLD	0.12	**DETECTED**	I	8	t189	*selx*	-	*sak, scn* (E)
ISTOP-460	ST188	QLD	0.06	-	I	8	t189	*selx*	-	*sak, chp, scn* (B)
ISTOP-464	ST188	QLD	0.06	-	I	8	T416	*selx*	-	*sak, chp, scn* (B)
ISTOP-467	ST188	QLD	0.06	-	I	8	Unknown	*sep, selx*	-	*sep, sak, scn* (G)
ISTOP-472	ST188	QLD	0.06	-	I	8	t189	*selx*	-	*sak, chp, scn* (B)
ISTOP-185	ST7259	NSW	0.06	-	I	ND	t189	*selx*	-	*sak, chp, scn* (B)
ISTOP-287	ST7271	NSW	0.125	-	I	8	t189	*sep, selx*	-	*sep, sak, scn* (G)
**Clonal Complex 291 (n=4)**										
ISTOP-55	ST291	NSW	0.12	-	I	5	t3096	*sea*	*edinB*	*sea, sak, chp, scn* (A)
ISTOP-326	ST291	VIC	≤0.03	-	I	5	t937		*edinB*	*sak, chp, scn* (B)
ISTOP-418	ST291	NSW	0.06	-	I	5	t16392		*edinB*	*sak, chp, scn* (B)
ISTOP-453	ST7287	NSW	0.12	-	I	5	t937		*edinB*	*sak, chp, scn* (B)
**Clonal Complex 361 (n=5)**										
ISTOP-108	ST672	NSW	≤0.03	-	I	8	t3841	*egc-cluster (seg+ sei+sem+sen+seo+selw), selx*	-	*sak, scn* (E)
ISTOP-131	ST672	ACT	0.06	-	I	8	t2379	*egc-cluster (seg+ sei+sem+sen+seo+selw), selx*	-	*sak, scn* (E)
ISTOP-151	ST672	WA	0.12	-	I	8	t14090	*egc-cluster (seg+ sei+sem+sen+seo+selw), selx*	-	*sak, chp, scn* (B)
ISTOP-222	ST672	QLD	0.06	-	I	8	t1309	*egc-cluster (seg+ sei+sem+sen+seo+selw), selx*	-	*sak, scn* (E)
ISTOP-302	ST672	NSW	0.06	-	I	8	t1309	*see, egc-cluster (seg+ sei+sem+sen+seo+selw), selx*	-	*sak, scn* (E)

Isolate	ST	STATE	Vitek 2 Pen MIC mg/L	*blaZ*	*agr* type	Capsule type	*spa*	Enterotoxins	Other Virulence Genes	IEC (Type)
**Clonal Complex 398 (n=16)**										
ISTOP-11	ST398	WA	0.06	-	I	5	t2928		-	*sak, scn (*E)
ISTOP-16	ST398	VIC	0.06	-	I	5	t1451		-	*chp, scn* (C)
ISTOP-97	ST398	VIC	≤0.03	-	UD	5	t1451		-	*chp, scn* (C)
ISTOP-132	ST398	NSW	0.12	-	I	5	t1130		-	*chp, scn* (C)
ISTOP-213	ST398	VIC	0.12	-	I	5	t1451		-	*chp, scn* (C)
ISTOP-214	ST398	VIC	0.06	-	I	5	Unknown		-	*chp, scn* (C)
ISTOP-226	ST398	WA	0.06	-	I	5	t1451		-	*chp, scn* (C)
ISTOP-240	ST398	SA	0.06	-	I	5	t6605		-	*chp, scn* (C)
ISTOP-304	ST398	QLD	0.12	-	I	5	t1451		-	*chp, scn* (C)
ISTOP-362	ST398	NSW	≤0.03	-	I	5	t1451		-	*chp, scn* (C)
ISTOP-363	ST398	NSW	0.06	-	I	5	t1451		-	*chp, scn* (C)
ISTOP-448	ST398	NSW	≤0.03	-	I	5	t1451		-	*chp, scn* (C)
ISTOP-98	ST3332	VIC	≤0.03	-	I	5	t011		-	*sak, chp, scn* (B)
ISTOP-100	ST3332	VIC	≤0.03	-	I	5	t011		-	*sak, chp, scn* (B)
ISTOP-323	ST7275	NSW	0.12	-	I	5	Unknown		-	*chp, scn* (C)
ISTOP-328	ST7277	NSW	0.06	-	I	5	t1451		-	*chp, scn* (C)
**Singletons (n=7)**										
ISTOP-146	ST425	ACT	0.06	-	II	5	Unknown	*selx*	-	*sak, chp, scn* (B)
ISTOP-162	ST2867	NSW	0.12	-	II	5	t2016	*selx*	*edinB*	*sak, scn* (E)
ISTOP-208	ST2867	QLD	0.12	-	II	5	Unknown	*selx*	*edinB*	-
ISTOP-288	ST2867	NSW	0.06	-	II	5	t148	*selx*	*edinB*	*sak, scn* (E)
ISTOP-190	ST5491	VIC	≤0.03	-	III	8	t5925	*selx*	-	*sak, scn* (E)
ISTOP-277	ST7270	NSW	0.06	-	I	5	Unknown	*seb, sep, selx, selz*	-	*sep, sak, scn* (G)
ISTOP-483	ST573	QLD	0.12	-	II	5	t1839	*Sec2+sel, egc-cluster (seg+ sei+sem+sen+seo+selw), selx, swl27, sel28*	-	*scn*
