# Supplementary material for: Molecular Epidemiology of Penicillin-Susceptible Staphylococcus aureus Bacteremia in Australia and Reliability of Diagnostic Phenotypic Susceptibility Methods to Detect Penicillin Susceptibility
Source: Microorganisms. 2022 Aug 15;10(8):1650. doi: 10.3390/microorganisms10081650 (PMC9413241; doi:10.3390/microorganisms10081650)
Supplement: Supplementary file 1 [file microorganisms-10-01650-s001.zip › Supplementary Table 4.pdf]

Table S4: BlaZ allotypes and BlaZ, BlaR1 and BlaI mutations identified in 45 *blaZ*- positive PSSA

Isolate	ST	CC	BlaZ allotype	BlaZ mutations	BlaR1 mutations	BlaI mutations
ATCC® 29213 (Reference)	ST5	CC5	A	-	-	-
ISTOP-23	ST7251	CC12	A	E26K, V77I, G145E	truncated (A466del)	-
ISTOP-27	ST3911	CC15	A	C220Y	truncated (A466del)	N110S
ISTOP-42	ST34	CC30	-	truncated (A92del)	-	D21G
ISTOP-50	ST582	CC15	A	K189N, C220Y	truncated (A466del)	N110S
ISTOP-53	ST6	CC6	C	A9V, I10M, P22S, I86V, G145E, K196N, M203F, S207N, S217P, C220Y, deletion (182-193)	truncated (A221ins)	-
ISTOP-84	ST7255	CC45	C	A9V, I10M, P22S, I86V, G145E, E193K, K196N, M203F, S207N, S217P, C220Y	-	-
ISTOP-88	ST45	CC45	-	truncated (A92del)	-	-
ISTOP-115	ST5059	CC15	A	C220Y	truncated (A466del)	N110S
ISTOP-121	ST3628	CC5	A	E26K, G145E	-	-
ISTOP-153	ST582	CC15	A	C220Y	truncated (A466del)	N110S
ISTOP-157	ST582	CC15	A	C220Y	truncated (A466del)	N110S
ISTOP-158	ST5	CC5	-	truncated (A92del)	-	-
ISTOP-164	ST8	CC8	-	truncated (A574del)	-	D21N, Y37N, S63H, E64N
ISTOP-171	ST3911	CC15	A	C220Y	truncated (A466del)	N110S

ISTOP-173	ST5	CC5	C	A9V, I10M, P22S, I86V, G145E, E193K, K196N, M203F, S207N, S217P, C220Y	truncated (A466del)	-
ISTOP-174	ST101	CC101	-	truncated (A92del)	-	-
ISTOP-187	ST5	CC5	A	E26K, V77I, G145E	truncated (A466del)	-
ISTOP-188	ST5	CC5	A	E26K, V77I, G145E	-	-
ISTOP-207	ST5	CC5	A	E26K, V77I, G145E	truncated (A466del)	-
ISTOP-210	ST3911	CC15	A	C220Y	truncated (A466del)	N110S
ISTOP-223	ST582	CC15	A	C220Y	truncated (A466del)	N110S
ISTOP-229	ST7262	CC5	B	F6L, N20T, P22S, D29N, H37N, S55A, I86V, T109A, T119K, K131N, V139I, Q141K, G145N, E193K, K196N, S207N, S217P, C220Y, V241I, G245N, V251I, S276N, M278I, K279N, E280K	-	D21N, Y37N, S63Y, E64N
ISTOP-233	ST5	CC5	C	A9V, I10M, P22S, I86V, G145E, E193K, K196N, M203F, S207N, S217P, C220Y	-	-
ISTOP-263	ST34	CC30	-	truncated (A92del)	-	D21G
ISTOP-264	ST34	CC30	-	truncated (A92del)	-	D21G
ISTOP-276	ST3911	CC15	A	C220Y	truncated (A466del)	N110S

ISTOP-293	ST9	CC9	-	truncated (A250ins)	-	D21N, Y37N, S63Y, E64N, N72I
ISTOP-295	ST3911	CC15	A	C220Y	truncated (A466del)	N110S
ISTOP-300	ST7273	CC15	A	C220Y	truncated (A466del)	N110S
ISTOP-306	ST582	CC15	A	C220Y	truncated (A466del)	N110S
ISTOP-318	ST582	CC15	A	C220Y	truncated (A466del)	N110S
ISTOP-319	ST30	CC30	A	A9V, P22S, I86V, K112A, G145E, C220Y	-	D21G
ISTOP-324	ST582	CC15	A	C220Y	truncated (A466del)	N110S
ISTOP-325	ST7276	CC25	-	truncated (A92del)	truncated (A466del)	D21G
ISTOP-360	ST22	CC22	B	F6L, N20T, P22S, D29N, H37N, S55A, I86V, T119K, K131N, V139I, Q141K, G145K, E193K, K196N, S207N, S217P, C220Y, V241I, G245N, V251I, S276N, M278I, K279N, E280K	-	D21N, Y37N, S63Y, E64N, N72I
ISTOP-401	ST7283	CC15	C	A9V, I10M, P22S, I86V, G145E, E193K, K196N, M203F, S207N, S217P, C220Y	truncated (E244STOP)	-
ISTOP-427	ST20	CC20	A	C220Y	truncated (A466del)	N110S
ISTOP-438	ST15	CC15	C	A9V, I10M, P22S, I86V, G145E, E193K,	-	-

				K196N, M203F, C220Y, G245V		
ISTOP-447	ST582	CC15	A	C220Y	truncated (A466del)	N110S
ISTOP-452	ST45	CC5	C	A9V, I10M, P22S, I86V, G145E, E193K, K196N, M203F, S207N, S217P, C220Y	-	-
ISTOP-458	ST188	CC188	C	A9V, P22S, L44N, I86V, G145E, E193K, K196N, M203F, C220Y	-	-
ISTOP-459	ST5	CC5	-	truncated (A92del)	-	-
ISTOP-462	ST582	CC15	A	C220Y	truncated (A466del)	N110S
ISTOP-465	ST582	CC15	A	C220Y	truncated (A466del)	N110S
ISTOP-475	ST582	CC15	A	C220Y	truncated (A466del)	N110S

>BlaZ_ATCC29213_UOH59618.1
MKKLIFLIAIALVLSACNSNSPHAKELNDLEKKYNAHIGVYALDTKSGKEVKFNSDKRFAYASTSKAINSAILLEQVPYNKLNKKIHINKDDIVAYSPILEKYVGKDITLKELIE
ASMTYSDNTANNKIIKEIGGIKKVKQRLKGLGDKVTNPVRYEIELNYYSPKSKKDTSTPAAFGKTLNKLIANGKLSKENKKFLLDLMLNNKSGDTLIKDGVSKDCKVADKS
GQAITYASRNDVAFVYPKGQSEPIVLVIFTNKDNKSDKPNDKLISETAKSVMKE

>BlaR1_ATCC29213_UOH59617.1
MAKLLITSVVSFCFIFLLLVFFKYILKRYFNYSLNYKVWYLTVLAGLIPFIPIKFSFIKFNNVNNQAPTVESKSHDLNHNINTTKPIQEFTTDIHKFNWDSIDNICTVVWIVLVII
LSFKFLKSLLYLKYLKKQSLYLNENEKNKVDTILFNHQYKKNIVIRKAETIQSPITFWYGKYIILIPSSYFKSVIDKRLKYIILHEYAHAKNRDTLHLIIFNIFSIVMSYNPLIHIVKR
KIIHDNEVEADRFVLNNINKNEFKTYAESIMDSVLNIPFFNKNILSHSFNGKKSLLKRRLINIKEANLKKQSKLIPIFICIFTFLLIVIQSQFLMGQSITDYNYKKPLQNDHQILD
ESKNFGSNSGSFVMYSMKKDKYYIYNEKESRKRYSPDSTYKIYLAMFGLDRHIISDKNSRMSWNHKHYPFESWNKEQDLNTAMQNSVNWYFERISNQIPKNYTAAQ
LKQLNYGNENLGSYKNYWMEDSLKISNLEQVIVFKNMMEQNNHFSKKAKNQLSSSLLIKKNEKYELYGKTGTGIVNGKYNNGWFVGYVITNHDKYYFATHLSDGNPS
GKNAELISEKILKEMGVLNGQ

>BlaI_ATCC29213_UOH59616.1
MANKQVEISMAEWDVMNIIWDKKSVSANEIVVEIQKYKEVSDKTIRTLITRLYKKEIIKRYKSENIYFYSSNIKEDDIKMTAKTFLNKLYGGDMKSLVLNFAKNEELNNK
EIEELRDILNDISKK
